# The wild bees (Hymenoptera, Apoidea) of the island of Cyprus

**DOI:** 10.3897/zookeys.924.38328

**Published:** 2020-04-06

**Authors:** Androulla I. Varnava, Stuart P.M. Roberts, Denis Michez, John S. Ascher, Theodora Petanidou, Stavroula Dimitriou, Jelle Devalez, Marilena Pittara, Menelaos C. Stavrinides

**Affiliations:** 1 Department of Agricultural Sciences, Biotechnology and Food Science, Cyprus University of Technology, Arch. Kyprianos 30, Limassol, 3036, Cyprus Cyprus University of Technology Limassol Cyprus; 2 CAER, School of Agriculture, Policy and Development, The University of Reading, Reading, UK The University of Reading Reading United Kingdom; 3 Research Institute of Bioscience, Laboratory of Zoology, University of Mons, Place du parc 23, 7000 Mons, Belgium University of Mons Mons Belgium; 4 Department of Biological Sciences, National University of Singapore, 14 Science Drive 4, Singapore 117543, Singapore National University of Singapore Singapore Singapore; 5 Laboratory of Biogeography & Ecology, Department of Geography, University of the Aegean, 81100 Mytilene, Greece University of the Aegean Mytilene Greece

**Keywords:** Bee species richness, biodiversity conservation, pollination, wild bees

## Abstract

Cyprus, the third largest island in the Mediterranean, constitutes a biodiversity hotspot with high rates of plant endemism. The wild bees of the island were studied extensively by the native George Mavromoustakis, a world-renowned bee taxonomist, who collected extensively on the island from 1916 to 1957 and summarised his results in a series of eight Cyprus-specific papers published from 1949 [“1948”] to 1957. The current work represents the first modern checklist of the wild bees of Cyprus, based on a compilation of previous publications, museum specimens and authors’ recent collections. Overall, 369 verified wild bee species have been recorded on the island, with eleven species reported from Cyprus for the first time. The island hosts all six of the globally widespread bee families, with Apidae represented by 110 species, Megachilidae with 91, Andrenidae with 76, Halictidae with 72, Colletidae with 19, and Melittidae with 1. Twenty-one of the recorded bee species are endemic (i.e., 5.7 % endemism rate) and Cyprus ranks third after Lesvos and Sicily in known bee species richness among the Mediterranean islands. Previously unpublished records from various locations on Cyprus for 156 previously reported bee species are also provided in the study. The current work provides a baseline for future studies of wild bee diversity on the island of Cyprus and neighbouring regions.

## Introduction

Cyprus, the third largest island in the Mediterranean, is situated in the eastern part of the basin, in the active tectonic zone between the African and the Eurasian plates. The island is a global biodiversity hotspot (Myers et al. 2000) characterised by high rates of plant endemism (Christofides 2017). Cyprus is an ideal place for the study of wild bees, because there is a wealth of past information on the diversity of species, a result of the considerable work of George Mavromoustakis, a world-renowned bee taxonomist native to the island.

The first list of the bee fauna of Cyprus was published by Pittioni (1950) who reported the results of a 1939 general scientific expedition on the island by Harald Lindberg, one of the most distinguished botanists in Finland in the 20^th^ century, and his two sons, Hakan and Par Harald. The most detailed records on the bee fauna of the island were compiled by Mavromoustakis in a series of eight papers that summarised his Cyprus records which included 237 currently valid species (Mavromoustakis 1949 [“1948”], 1951, 1952, 1953, 1954, 1955, 1957a, 1958 [“1957”]). The first paper in the series is commonly cited according to its imprint date of 1948, but its date of publication as established for nomenclatural purposes by Evenhuis (2003) is 14 January 1949. Some other Mavromoustakis papers also have ICZN dates postdating their imprint dates as notes. Mavromoustakis’ data included in his eight Cyprus-specific papers are currently available in an accessible online database (Varnava and Stavrinides 2015), which includes data on the location, month and plant species on which bees were collected. The wild bee species had been reported by Mavromoustakis to visit 177 different species of plants, of which 13 are endemic (Varnava and Stavrinides 2015). Georghiou (1977) listed all the bees reported on the island by Pittioni (1950), as well as some species in the collections of the Department of Agriculture, virtually all of which were collected and identified by Mavromoustakis.

Recent work on selected genera of wild bees of Cyprus was carried out by Ebmer (2014). Ascher and Pickering’s (2018) online compilation lists 335 species of wild bees on the island, while Kuhlmann et al. (2015) report 305 species. The aim of the current work is to provide the first comprehensive checklist of the bees of Cyprus, including all past information on authors’ reports for each species, enriched with lists of new records and species collected by the authors of the current study.

## Materials and methods

### Study area

The geological history of Cyprus comprises a series of complex geological processes that started approximately 90 million years ago (mya), when the subduction of the African plate beneath the Eurasian created new oceanic crust; this was later cut off and uplifted to create the Troodos range, in the centre of the island, with a peak at 1,951 m (Wagstaffe 2016). The uplifting of limestone depositions created the Pendadaktylos range in the north of the country, with a maximum elevation of 1,024 m. The current form of the island took shape approximately two mya.

Pollen evidence suggests that early Holocene Cyprus was covered by dense stands of typical Mediterranean trees and shrubs, such as *Ceratonia siliqua*, *Quercus coccifera*, *Quercus infectoria*, *Laurus nobilis*, *Olea europaea, Cupressus sempervirens* and species in the genus *Juniperus* (Delipetrou et al. 2008). Noteworthy extinct species of animals that lived on the island during the Pleistocene until around 11,000 years BP include the Cyprus dwarf elephant (*Elephas cypriotes*) and Cyprus pygmy hippopotamus (*Phanourios minutus*), which probably arrived on the island by swimming from the mainland (Nicolaou et al. 2016).

The current land surface area of Cyprus is 9,251 km^2^, of which 42 % is covered with forest/shrubland, and 23 % is devoted to agriculture (Vogiatzakis et al. 2016). There are 52 different habitat types listed in the EU Habitats Directive (92/43/EC), with five of them being exclusive to the island. The island is very rich in plant diversity with more than 1,900 plant species, of which 130 are endemic (Christofides 2017). Most of the endemic species of plants occur on the Troodos and Pentadaktylos mountain ranges.

There are 29 species of terrestrial mammals recorded on Cyprus, with bats represented by 19 species (Nicolaou et al. 2016). There are nine species of snakes, 11 species of lizards (one endemic species) (Sparrow and Baier 2016a), three species of native turtles (Sparrow and Baier 2016a), and three species of amphibians (one endemic) (Sparrow and Baier 2016b). Among the 412 bird species that have been recorded on Cyprus, 57 are resident breeders with two endemic species that breed nowhere else: the Cyprus wheatear (*Oenanthe cypriaca*) and the Cyprus warbler (*Sylvia melanothorax*), with an additional of four endemic subspecies (Charalambidou et al. 2016).

Insects in Cyprus represent a highly diverse group with more than 5,000 recorded species according to Fauna Europaea (2012), with the number of species per order varying in more recent accounts. Coleoptera is the most species-rich order, followed by Hymenoptera and Lepidoptera. Well-studied groups include Odonata with 37 species (Sparrow et al. 2016), Orthoptera with 71 species of which 12 are endemic (Siedle et al. 2016), and butterflies with 49 species of which three are endemic (John 2016).

### Methodology

The updated checklist was based on published records of wild bees and species present in authors’ personal collections and in those of numerous correspondents. The earliest published descriptions based on Cyprus specimens date to 1870 (Dours, 1870) and 1910 (Cockerell, 1910), however these include specimens collected earlier, as it was very common for ornithologists or sometimes palaeontologists working for European museums to collect different taxa during their expeditions and provide them to experts for identification.

We used as a starting point for this survey a compilation of species occurrence records available for the island by J.S Ascher, accessible online through the website Discover Life (Ascher and Pickering 2018) some of which had associated specimens records captured through the project on the collaborative databasing of North American bee collections within a global informatics network (Ascher 2016). Each species in this initial list was evaluated by the experts, and species were retained in the checklist only if a primary paper based on examined specimens confirmed the presence of the species on the island. In addition, we retained in the checklist species for which a reference to a specific museum specimen was available.

Furthermore, a review of the literature known to the authors to cover Cyprus wild bee species was made to identify additional species recorded from the island. In general, we avoided redundant citation of distributional listings in global or regional compilations (e.g., Ascher and Pickering 2018, Kuhlmann et al. 2015) or other secondary sources, such as Gusenleitner and Schwarz (2002) and Müller (2018). However, for some species for which no records for Cyprus existed in primary sources and which the authors of the study considered valid for Cyprus, we cited secondary sources.

Published works covering the wild bees of Cyprus as known to the authors were listed in chronological order under each species referenced. Georghiou (1977) was used as a reference only for species that were collected/examined in the Mavromoustakis collection during the Georghiou survey, but not for species whose presence in Cyprus was based on other published works (notably Pittioni, 1950).

**Figure 1.**
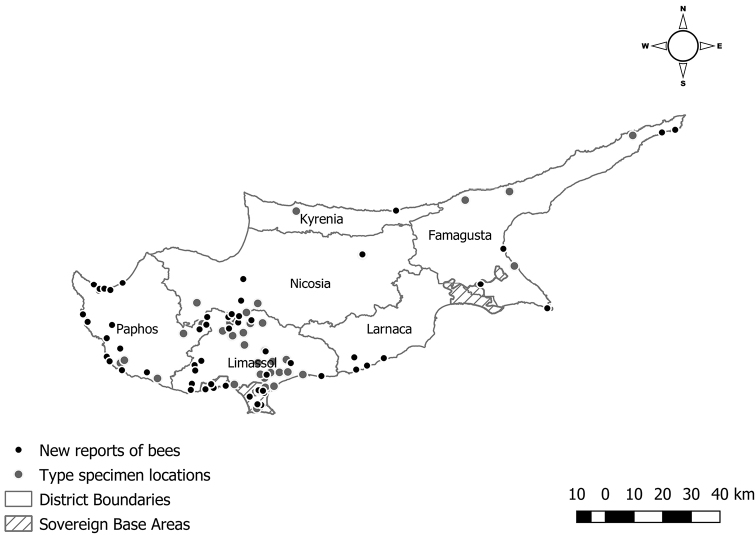
Map of Cyprus indicating locations of type specimens (for both valid and synonymised taxa) and unpublished records. See Supplem. material 1, Table S3, S4 for the geographic coordinates. The new reports of bees are shown with black dots and type specimen locations with grey dots.

**Figure 2.**
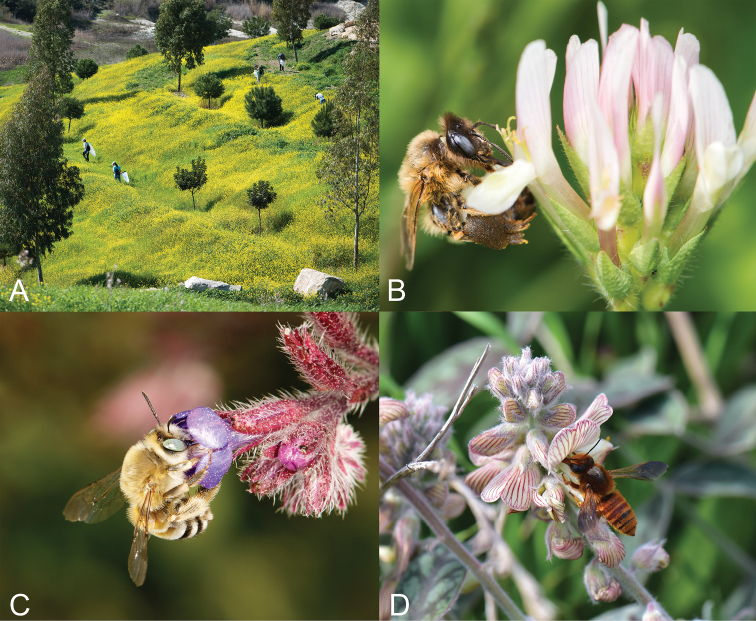
**A** Landscape with diverse bee fauna in Cyprus (Yermasoyia Dam area) **B***Melitturga syriaca* foraging on *Trifolium clypeatum***C***Eucera dimidiata* foraging on Anchusa undulata subsp. hybrida**D***Megachile cypricola* foraging on *Onobrychis venosa*. Pictures provided by Nicolas J. Vereecken (**A, B, C**) and Androulla Varnava (**D**).

Experts’ samplings were carried out at different sites on the island (Fig. 1) by hand-netting and pan trapping. Sampling locations were concentrated on the southern part of the island, where the Republic of Cyprus exercises effective control (see Fig. 2A for an example of a sampling location). Hand-netting focused on collecting specimens during their visits to flowering plants (e.g., Fig. 2B-D). For each species from the personal collection of authors we provide information on the collector (leg.), expert who identified the specimen (det.), locality, collection day, number of individuals of each sex and the collection specific number (museum collections/authors collections) when available. A list of museum acronyms is available in Suppl. material 1, Table S1.

For species for which Cyprus is the type locality, we provide details on the type locality, status, repository, and collection event information (i.e., collector and date of collection), when available, extracted from a compilation of type data for world bees (J. S. Ascher, unpublished). For taxa described from Cyprus, we note the name under which the specimen was described from the island, to highlight the potentially interesting variation within the taxon. In addition, collection localities are provided for all specimens from the Mavromoustakis collection. Virtually all GPS coordinates for type specimens and specimens from the Mavromoustakis collection represent the centre of the administrative region of the village / city from where samples were collected, as no GPS data were available at the time of collection. For the unpublished records, the horizontal distance (30 m) from the given decimal latitude and decimal longitude describe the smallest circle containing the whole of the location.

The global distribution of each species is reported based on IUCN (2019) with additional data from Ascher and Pickering (2018) in cases where IUCN data did not list all continents from which a species was reported. Ascher and Pickering (2018) data were used to include additional continents in the distribution but not to amend the distribution data at country level for continents reported in IUCN (2019). We recognise that species distribution is dynamic, and therefore there might be differences between the actual distribution and the one described in the current study. Countries are grouped into geographic regions based on a United Nations categorisation (United Nations 2017 - ST/ESA/SER.A/408 – see Suppl. material 1, Table S2). Specific countries are listed when the species is present in less than four countries within the geographic region. The nomenclature in the present work follows Nieto et al. (2014) for species names, Michener (2007) for most supra-specific taxa, and Dorchin et al. (2018) for the tribe Eucerini.

## Results

The present checklist for the Cyprus wild bee fauna includes a total of 369 species with confirmed records, 21 of which are endemic to Cyprus. Eleven species are reported for the first time from the island: Andrena (Taeniandrena) leucopsis Warncke, 1967, A. (Chlorandrena) gordia Warncke, 1975, Anthophora (Pyganthophora) dalmatica Pérez, 1902, *Colletes creticus* Noskiewicz, 1936, Eucera (Hetereucera) aequata Vachal, 1907, E. (Eucera) palaestinae Friese, 1922, E. (Eucera) sulamita Vachal, 1907, Megachile (Eutricharaea) inexspectata Rebmann, 1968, *Thyreus picaron* Lieftinck, 1968, *Sphecodes ephippius* (Linnaeus, 1767) and *S. pseudofasciatus* Blüthgen, 1925.

Additionally, unpublished records for 156 species of bees previously reported on the island are provided. The 369 wild bee species are classified into six families as follows: Apidae 110, Megachilidae 91, Andrenidae 76, Halictidae 72, Colletidae 19, and Melittidae 1 species. Most of the 21 endemic species belong to the Andrenidae and Apidae families. We also provide a list of 29 additional species (see relevant section of Supplementary Material) for which there is high uncertainty for their presence on the island, even though they had been reported as present in Cyprus in the past.

A total of 101 species or subspecies were described from Cyprus (80 of which by G. Mavromoustakis) from 44 distinct locations (Fig. 1). Currently, 46 are valid at the species, and 18 at the subspecies level (Suppl. material 1, Table S3). Most type locations are concentrated on the southern part of the island, in the District of Limassol, where Mavromoustakis resided and recent collections were carried out. Most of the type insects from Cyprus (52) are located at the Department of Agriculture collection in Nicosia (Cyprus Department of Agriculture, 1989).

### Updated checklist of the bees of Cyprus

**MELITTIDAE**

**DASYPODAINI**

**Genus *Dasypoda* LATREILLE, 1802**

1 species.

**Dasypoda (Megadasypoda) suripes (Christ, 1791)**

**References.**Michez et al. (2004).

**Distribution.** Cyprus, Western Europe (Austria), Southern Europe (Albania, Italy), Northern Europe (Lithuania), Eastern Europe, Western Asia (Turkey, Armenia).

**Notes.***D. suripes* has been considered by some authors to be a nomen dubium and therefore the species has been cited in some sources as *D. aurata* Rudow, 1881.

**ANDRENIDAE**

**ANDRENINI**

**Genus *Andrena* FABRICIUS, 1775**

73 species.

**Andrena (Aciandrena) aciculata Morawitz, 1886**

**References.**Scheuchl and Willner (2016).

**Material examined.** Limassol District: Polemidia, 34.71178°N, 33.004775°E, 8.III.2017, (1♀); Sovereign Base Area, Akrotiri, Bishop's Pool, 34.597305°N, 32.984521°E, 10.III.2017, (1♀); Sovereign Base Area, Akrotiri, 34.600657°N, 32.971419°E, 10.III.2017, (2♀); Anogyra to Pachna Road km 4, 34.764269°N, 32.757736°E, 5.V.2015, (1♀), all records S.P.M Roberts leg., B. Tomozei det.

**Distribution.** Cyprus, Western Europe (Austria), Southern Europe (North Macedonia, Greece), Eastern Europe, Western Asia (Turkey), Southern Asia (Iran).

**Andrena (Suandrena) aegypticola Friese, 1922**

**References.**Mavromoustakis (1954); Warncke (1967).

**Mavromoustakis localities.** Famagusta, Larnaca.

**Distribution.** Cyprus, Northern Africa (Libya), Western Asia.

**Notes.** Described from Cyprus as *Andrena larnacensis*Mavromoustakis 1954, from Famagusta: 35.125°N, 33.941667°E, 15.II.1951, G.A. Mavromoustakis leg., G.A. Mavromoustakis det. ♀, (DAAN).

**Andrena (Aenandrena) aeneiventris Morawitz, 1872**

**References.** Mavromoustakis (1949 [“1948”]); Pittioni (1950); Mavromoustakis (1951, 1952, 1957a, b); Georghiou (1977); Scheuchl and Willner (2016).

**Mavromoustakis localities.** Limassol, Polemedia Hills, Zakaki, Asomatos, Fassouri, Yermasoyia river, Salamis, Near Pano Kivides, Kaloiri Hills near Yermasoyia River, Morphou.

**Distribution.** Cyprus, Western Europe (France, Switzerland, Austria), Southern Europe, Eastern Europe, Northern Africa, Central Asia, Western Asia.

**Andrena (Melandrena) albopunctata (Rossi, 1792)**

**References.**Gusenleitner and Schwarz (2002).

**Distribution.** Cyprus, Western Europe (France), Southern Europe, Eastern Europe, Northern Africa, Western Asia, Central Asia, Southern Asia (Iran, Afghanistan, Pakistan).

**Andrena (Micrandrena) alfkenelloides Warncke, 1965**

**References.**Gusenleitner and Schwarz (2002).

**Material examined.** Limassol District: 1 km E of Pissouri, 34.677579°N, 32.722066°E, 27.IV.2015, (3♀, 1♂), collecting pollen on Brassicaceae; 0.5 km E of Vasa, 34.831384°N, 32.79744°E, 29.IV.2015, (1♀), visiting Apiaceae; Anogyra to Avdimou Road km 2, 34.723986°N 32.736892°E, 3.V.2015, (21♀), collecting pollen and visiting Apiaceae; 0.7 km N of Anogyra, 34.745537°N, 32.73385°E, 3.V.2015, (14♀, 1♂), collecting pollen and visiting Apiaceae; Anogyra to Pachna Road km 4, 34.764269°N, 32.757736°E, 5.V.2015, (3♀), collecting pollen at Apiaceae, all records S.P.M. Roberts leg., B. Tomozei det.

**Distribution.** Cyprus, Southern Europe (Greece), Eastern Europe (Bulgaria), Western Asia (Turkey).

**Andrena (Chlorandrena) astica Warncke, 1967**

**References.**Gusenleitner and Schwarz (2002); Schwenninger (2015).

**Distribution.** Cyprus, Southern Europe (Greece), Western Asia (Turkey).

**Andrena (Euandrena) bicolor Fabricius, 1775**

**References.**Warncke (1965, 1975); Scheuchl & Willner (2016).

**Material examined.** Limassol District: Chionistra, 34.9317°N, 32.8664°E, 14.V.2012, 16.V.2012, S. Dimitriou leg., E. Scheuchl det. (1♂, 1♀), pan trap (UAEG).

**Distribution.** Cyprus, Widespread in Europe, Northern Africa (Morocco, Algeria, Tunisia), Western Asia (Turkey, Israel), Central Asia (Kazakhstan), Southern Asia (Iran), Eastern Asia (China).

**Notes.** Described from Cyprus as Andrena bicolor ssp. apricariaWarncke 1975, from Limassol: 34.66839°N, 33.03252°E, II.1939, K. Warncke leg./det. ♀, (OLML).

**Andrena (Cryptandrena) brumanensis Friese, 1899**

**References.** Mavromoustakis (1949 [“1948”]); Pittioni (1950); Mavromoustakis (1951, 1952).

**Material examined.** Limassol District: 0.7 km N of Anogyra, 34.745537°N, 32.73385°E, 3.V.2015, (1♀), collecting pollen on Apiaceae; Yermasoyia Dam, 34.755799°N, 33.096194°E, 7.III.2017, all records S.P.M. Roberts leg., B. Tomozei det. (10♀).

**Distribution.** Cyprus, Western Europe (France), Southern Europe, Eastern Europe (Hungary, Romania, Slovakia), Western Asia (Turkey).

**Andrena (Truncandrena) caneae Strand, 1915**

**References.**Pittioni (1950); Mavromoustakis (1953, 1957a); Warncke (1967).

**Mavromoustakis localities.** Limassol, Amathus, Yerasa, Polemedia, Famagusta.

**Distribution.** Cyprus, Southern Europe (Greece), Western Asia (Turkey).

**Notes.** Described from Cyprus as *Andrena mavromoustakisi* Pittioni, 1950, from Geroskipou, E of Paphos: 34.76666°N, 32.46666°E, 8.V.1946, G.A. Mavromoustakis leg., B. Pittioni det., (MZHF).

**Andrena (Micrandrena) cervina Warncke, 1975**

**Type locality–country.** Cyprus, Limassol: 34.66839°N, 33.03252°E, 10.IV.1967, G.A. Mavromoustakis leg., K. Warncke det. ♀, (OLML).

**References.**Warncke (1975).

**Material examined.** Limassol District: 2 km N of Anogyra, 34.748126°N, 32.732248°E, 1.V.2015, (1♀); Famagusta District: Cape Greco, 34.963264°N, 34.066211°E, 15.III.2017, (3♂), all records S.P.M. Roberts leg., B. Tomozei det.

**Distribution. Cyprus. ENDEMIC.**

**Andrena (Aenandrena) chaetogastra Pittioni, 1950**

**Type locality–country.** Cyprus, Mt. Troodos, Chionistra: 34.9364°N, 32.8636°E, 17.VI.1939, H. Lindberg leg., B. Pittioni det. ♀, (MZHF).

**References.**Pittioni (1950).

**Distribution.** Cyprus, Western Asia (Israel).

**Andrena (Chlorandrena) cinereophila Warncke, 1965**

**References.**Warncke (1965).

**Distribution.** Cyprus, Southern Europe (North Macedonia, Greece), Eastern Europe (Romania, Bulgaria), Western Asia (Israel), Southern Asia (Afghanistan).

**Andrena (Brachyandrena) colletiformis Morawitz, 1874**

**References.** Mavromoustakis (1949 [“1948”]); Pittioni (1950); Mavromoustakis (1952, 1957a); Warncke (1967).

**Mavromoustakis localities.** Limassol, Cherkes, Yermasoyia River, Amathus, Salamis, Larnaca.

**Distribution.** Cyprus, Western Europe (France), Southern Europe, Eastern Europe, Northern Africa (Morocco, Tunisia, Algeria), Western Asia (Turkey, Israel), Central Asia (Turkmenistan, Kazakhstan), Southern Asia (Iran).

**Notes.** Described from Cyprus as *Andrena colletiformis insulana* Pittioni, 1950, from Kouklia: 34.6978°N, 32.592°E, 26.VI.1939, H. Lindberg leg., B. Pittioni det., (MZHF).

**Andrena (Simandrena) combinata (Christ, 1791)**

**References.** Mavromoustakis (1949 [“1948”], 1951, 1952, 1954, 1958 ["1957"]); Scheuchl and Willner (2016).

**Mavromoustakis localities.** Limassol, Cherkes, Yermasoyia River, Amathus, Yerasa, Lania, Near Paramytha, Northern Mountains Kantara, Kellaki, Morphou (West Mesaoria plains), Larnaca.

**Material examined.** Limassol District: Makria Kontarka, 34.9095°N, 32.8971°E, 31.V.2012, 2.VI.2012 (1♀), pan trap (UAEG), all records S. Dimitriou leg., E. Scheuchl det.

**Distribution.** Cyprus, Widespread in Europe, Northern Africa (Algeria), Western Asia, Central Asia, Southern Asia (Iran), Eastern Asia (China).

**Andrena (Chlorandrena) crepidis Schwenninger, 2015**

**Type locality–country.** Cyprus, Limassol District, 4 km SW Kaminaria: 34.904°N, 32.761°E, 24.III.2013, H.R. Schwenninger leg., H.R. Schwenninger det. ♂, (SMNS).

**References.**Schwenninger (2015).

**Material examined.** Limassol District: Kaminaria, 34.93°N, 32.78°E, 22.III.2013, (1♂); Kaminaria, 34.93°N, 32.78°E, 24.III.2013, (1♂); 4 km SW Kaminaria, 24.III.2013, (1♂); 5 km SW Kaminaria, 24.III.2013, (1♀, 1♂); Kaminaria, 34.93°N, 32.78°E, 2.IV.2009 (2♂); Amiantos, 34.918°N, 32.9472°E, 5-7.IV.2012 (1♂), pan trap (UAEG); Nicosia District: Kakopetria, 34.992°N, 32.9082°E, 5-7.IV.2012, (3♂), pan trap (UAEG); Kakopetria, 34.992°N, 32.9082°E, 25-27.IV.2012, (1♀), pan trap (UAEG), all Kaminaria records H.R. Schwenninger leg., H.R. Schwenninger det., all Amiantos records S. Dimitriou leg., E. Scheuchl det., all Kakopetria records S. Dimitriou leg., E. Scheuchl and J. Devalez det.

**Distribution. Cyprus. ENDEMIC.**

**Andrena (Notandrena) curvana Warncke, 1965**

**References.**Scheuchl and Willner (2016).

**Distribution.** Cyprus, Western Europe (Austria), Southern Europe, Eastern Europe.

**Andrena (Cordandrena) cypria Pittioni, 1950**

**Type locality–country.** Cyprus, Mt. Troodos, Chionistra: 34.9364°N, 32.8636°E, 17.VI.1939, H. Lindberg leg., B. Pittioni det. ♀, (MZHF).

**References.**Pittioni (1950); Mavromoustakis (1953).

**Mavromoustakis localities.** Mt. Troodos.

**Distribution.** Cyprus, Western Asia, Southern Asia (Iran).

**Andrena (Plastandrena) cypricola Mavromoustakis, 1952**

**Type locality–country.** Cyprus, Kato Amiandos, 762–914m: 34.9059°N, 32.9431°E, 4.IV.1946, G.A. Mavromoustakis leg., G.A. Mavromoustakis det. ♀, (DAAN).

**References.**Pittioni (1950); Mavromoustakis (1952, 1953, 1954); Warncke (1967).

**Mavromoustakis localities.** Pera Pedi, Yermasoyia River, Trimiklini, Yerasa, Saettas, Ayia Phyla, Amyrou Monastery-near Apsiou, Amathus, Rotsou spring-near Paramytha, Kato Amiandos, Mt. Troodos.

**Material examined.** Nicosia District: Linou, 35.0755°N, 32.9164°E, 5-7.IV.2012, (1♀), pan trap (UAEG); Kakopetria, 34.992°N, 32.9082°E, 5-7.IV.2012, (1♀), pan trap (UAEG), all records S. Dimitriou leg., E. Scheuchl det.

**Distribution. Cyprus. ENDEMIC.**

**Andrena (Micrandrena) dargia Warncke, 1965**

**References.**Gusenleitner and Schwarz (2002).

**Distribution.** Cyprus, Southern Europe (Greece), Western Asia (Turkey).

**Andrena (Holandrena) decipiens Schenck, 1861**

**References.** Mavromoustakis (1949 [“1948”]); Warncke (1965); Scheuchl and Willner (2016).

**Mavromoustakis localities.** Limassol, Fassouri, Amathus, Akrounda, Nicosia.

**Distribution.** Cyprus, Western Europe, Southern Europe, Eastern Europe, Northern Africa, Western Asia (Turkey), Southern Asia (Iran).

**Andrena (Melandrena) elmaria Gusenleitner, 1998**

**References.** Gusenleitner (1998).

**Material examined.** Limassol District: Yermasoyia Dam, 34.755799°N, 33.096194°E, 7.III.2017, (1♂); Sovereign Base Area Akrotiri, Bishop's Pool, 34.597305°N, 32.984521°E, 10.III.2017, (1♀, 1♂); Famagusta District: Cape Greco, 34.963264°N, 34.066211°E, 15.III.2017, (1♀), all records S.P.M. Roberts leg., B. Tomozei det.

**Distribution.** Cyprus, Western Asia, Southern Asia (Iran).

**Andrena (Parandrenella) figurata Morawitz, 1866**

**References.**Gusenleitner and Schwarz (2002).

**Distribution.** Cyprus, Southern Europe, Eastern Europe, Western Asia (Turkey, Georgia, Azerbaijan), Southern Asia (Iran).

***Andrena flavilabris* Schenck, 1874**

**References.**Scheuchl and Willner (2016).

**Distribution.** Cyprus, Western Europe (France, Switzerland, Germany), Southern Europe (Spain), Eastern Europe (Ukraine), Western Asia.

**Andrena (Zonandrena) flavipes Panzer, 1799**

**References.** Mavromoustakis (1949 [“1948”]); Pittioni (1950); Mavromoustakis (1951, 1952, 1953, 1954, 1957a); Georghiou (1977); Scheuchl and Willner (2016).

**Mavromoustakis localities.** Limassol, Polemedia Hills, Cherkes, Zakaki, Fassouri, Trimiklini, Eftagonia, Pissouri, Yerasa, Lania, Platania Forest Station, Fasoula, Northern Mountains Kantara, Larnaca, sand dunes near Amathus.

**Material examined.** Nicosia District: Kakopetria, 34.992°N, 32.9082°E, 5-7.IV.2012, (3♂, 2♀), pan trap (UAEG); Kakopetria, 34.992°N, 32.9082°E, 14-16.V.2012, (2♀), pan trap (UAEG); Kakopetria, 34.992°N, 32.9082°E, 31.V.2012, 2.VI.2012, (1♀), pan trap (UAEG); Limassol District: Amiantos, 34.918°N, 32.9472°E, 5-7.IV.2012, (12♂, 3♀), pan trap (UAEG); Amiantos, 34.918°N, 32.9472°E, 14-16.V.2012, (1♂, 1♀), pan trap (UAEG); Amiantos, 34.918°N, 32.9472°E, 31.V.2012, 2.VI.2012, (1♀), pan trap (UAEG); Makria Kontarka, 34.9095°N, 32.8971°E, 14-16.V.2012, (31♂, 3♀), pan trap (UAEG); Makria Kontarka, 34.9095°N, 32.8971°E, 31.V.2012, 2.VI.2012 (4♂, 17♀), pan trap (UAEG); Almirolivado, 34.9333°N, 32.9004°E, 14-16.V.2012, (58♂, 13♀), pan trap (UAEG); Almirolivado, 34.9333°N, 32.9004°E, 31.V.2012, 2.VI.2012, (1♂, 12♀), pan trap (UAEG); Troodos, Chionistra, 34.9317°N, 32.8664°E, 14-16.V.2012, (77♂, 1♀), pan trap (UAEG); Troodos, Chionistra, 34.9317°N, 32.8664°E, 31.V.2012, 2.VI.2012, (8♂, 40♀), pan trap (UAEG); Anogyra to Pachna Road km 4, 34.764269°N, 32.757736°E, 5.V.2015, (1♀); Yermasoyia Dam, 34.755799°N, 33.096194°E, 7.III.2017, (1♀, 1♂); Paphos District: Nr Arminou Reservoir, 34.883435°N, 32.750988°E, 29.IV.2015, (3♀), collecting pollen on Brassicaceae; 2.7 km SW of Acheleia, Potamos tis Ezouzas, 34.729004°N, 32.457544°E, 30.IV.2015, (1♀, 1♂), all Kakopetria, Amiantos, Makria Kontarka, Almirolivado and Troodos-Chionistra records S. Dimitriou leg., J. Devalez det., all Anogyra, Yermasoyia Dam and Paphos district records S.P.M. Roberts leg., B. Tomozei det.

**Distribution.** Cyprus, Widespread in Europe, Northern Africa, Western Asia, Central Asia, Southern Asia (India, Nepal), Eastern Asia (China).

**Andrena (Holandrena) forsterella Osytshnjuk, 1978**

**References.**Schönitzer et al (1995); Gusenleitner and Schwarz (2002).

**Distribution.** Cyprus, Southern Europe, Eastern Europe (Bulgaria), Western Asia, Southern Asia (Iran).

**Notes.** In older literature often not separated from *A. variabilis*.

**Andrena (Melanapis) fuscosa Erichson, 1835**

**References.**Cockerell (1910); Mavromoustakis (1951, 1952, 1954); Warncke (1967); Scheuchl and Willner (2016).

**Mavromoustakis localities.** Episkopi, Zakaki, Cherkes, Fassouri, Famagusta, Larnaca.

**Material examined.** Famagusta District: Cape Greco, 34.963264°N, 34.066211°E, 15.III.2017, S.P.M. Roberts leg., B. Tomozei det. (7♂).

**Distribution.** Cyprus, Widespread in Europe, Northern Africa, Western Asia, Central Asia Southern Asia (India).

**Notes.** Described from Cyprus as *Andrena cyprica*Cockerell 1910, from Nicosia: 35.166667°N, 33.366667°E, 17.III, C. Glaszner leg., T.D.A. Cockerell det. ♂, (NHMUK).

**Andrena (Ptilandrena) glidia Warncke, 1965**

**References.**Warncke (1974); Gusenleitner and Schwarz (2002).

**Material examined.** Nicosia District: Kakopetria, 34.992°N, 32.9082°E, 5-7.IV.2012, (1♀), pan trap (UAEG); Limassol District: Almirolivado, 34.9333°N, 32.9004°E, 14-16.V.2012, (2♀), pan trap (UAEG); Troodos, Chionistra, 34.9317°N, 32.8664°E, 14-16.V.2012, (3♀), pan trap (UAEG); Troodos, Chionistra, 34.9317°N, 32.8664°E, 31.V.2012, 2.VI.2012, (3♀), pan trap (UAEG), all records S. Dimitriou leg., E. Scheuchl det.

**Distribution.** Cyprus, Southern Europe (Greece), Western Asia (Turkey, Israel).

**Andrena (Chlorandrena) gordia Warncke, 1975**

**Material examined.** Paphos District: Nr Arminou Reservoir, 34.883435°N, 32.750988°E, 29.IV.2015, (1♂), S.P.M. Roberts leg., B. Tomozei det.

**Distribution.** Cyprus, Western Asia (Turkey).

**Andrena (Melandrena) grandilabris Pérez, 1903**

**References.**Warncke (1969, 1974).

**Distribution.** Cyprus, Western Asia.

**Andrena (Chrysandrena) hesperia Smith, 1853**

**References.** Mavromoustakis (1949 [“1948”], 1951, 1952, 1953, 1957a); Scheuchl and Willner (2016).

**Mavromoustakis localities.** Limassol, Akrotiri Forest, Fassouri, Amathus, Yermasoyia River, Yerasa, Omodos, Trimiklini, Mesayitonia-Fasoula, Amyrou Monastery (near Apsiou), Nicosia, Larnaca, Famagusta.

**Material examined.** Limassol District: Amiantos, 34.918°N, 32.9472°E, 25-27.IV.2012, (1♀), pan trap (UAEG); Sovereign Base Area, Akrotiri, 34.600657°N, 32.971419°E, 17.III.2017, (2♀), collecting pollen on *Crepis sancta* (Asteraceae); Sovereign Base Area, Avdimou Bay Cliffs, 34.656698°N, 32.773339°E, 13.III.2017, (1♀), all Amiantos records S. Dimitriou leg., E. Scheuchl det., all Akrotiri and Avdimou Bay records S.P.M. Roberts leg., B. Tomozei det.

**Distribution.** Cyprus, Widespread in Europe, Northern Africa, Western Asia (Turkey, Israel), Central Asia.

**Andrena (Margandrena) hyacinthina Mavromoustakis, 1958**

**Type locality–country.** Cyprus, Yermasoyia River plain: 34.7182°N, 33.08788°E, 3.III.1952, G.A. Mavromoustakis leg., G.A. Mavromoustakis det. ♀, (DAAN).

**References.**Pittioni (1950); Mavromoustakis (1958); Warncke (1967).

**Material examined.** Limassol District: Yermasoyia Dam, 34.755799°N, 33.096194°E, 7.III.2017, S.P.M. Roberts leg., B. Tomozei det. (1♀).

**Distribution.** Cyprus, Western Asia (Turkey, Israel, Lebanon).

**Andrena (Ptilandrena) kornosica Mavromoustakis, 1954**

**Type locality–country.** Cyprus, Mt. Kornos (Northern Mountains), 762m: 35.1379°N, 33.1379°E, 3.III.1936, G.A. Mavromoustakis leg., G.A. Mavromoustakis det. ♀, (NHMUK).

**References.**Pittioni (1950); Mavromoustakis (1954, 1957a); Warncke (1967).

**Mavromoustakis localities.** Mt. Kornos, Lania, Near Trooditissa.

**Distribution. Cyprus. ENDEMIC.**

**Andrena (Aciandrena) lamiana Warncke, 1965**

**References.**Warncke (1965).

**Material examined.** Limassol District: Anogyra, 34.741952°N, 32.734845°E, 3.V.2015, S.P.M. Roberts leg., B. Tomozei det. (1♀), collecting pollen on Brassicaceae.

**Distribution.** Cyprus, Southern Europe (North Macedonia, Greece), Eastern Europe (Bulgaria), Western Asia (Turkey, Syria), Southern Asia (Iran).

**Andrena (Simandrena) lepida Schenck, 1861**

**References.**Warncke (1965); Scheuchl and Willner (2016).

**Distribution.** Cyprus, Widespread in Europe, Northern Africa, Western Asia (Turkey, Israel, Syria), Southern Asia (Iran).

**Andrena (Taeniandrena) leucopsis Warncke, 1967**

**Material examined.** Limassol District: Almirolivado, 34.9333°N, 32.9004°E, 14-16.V.2012, S. Dimitriou leg., E. Scheuchl det. (2♀), pan trap (UAEG).

**Distribution.** Cyprus, Southern Europe (North Macedonia, Greece), Eastern Europe (Romania, Bulgaria), Western Asia (Turkey, Lebanon), Southern Asia (Iran).

**Andrena (Poecilandrena) limassolica Mavromoustakis, 1949**

**Type locality–country.** Cyprus, Limassol: 34.66839°N, 33.03252°E, 19.I.1936, G.A. Mavromoustakis leg., G.A. Mavromoustakis det. ♀, (DAAN).

**References.** Mavromoustakis (1949 [“1948”], 1952); Warncke (1967).

**Mavromoustakis localities.** Limassol, Polemedia Hills.

**Distribution.** Cyprus, Western Asia (Jordan).

**Andrena (Melandrena) limata Smith, 1853**

**References.**Cockerell (1910, 1914); Mavromoustakis (1953, 1954, 1957a); Warncke (1967); Scheuchl and Willner (2016).

**Mavromoustakis localities.** Limassol, Zakaki, Pera Pedi.

**Material examined.** Limassol District: Sovereign Base Area Akrotiri, Bishop's Pool, 34.597305°N, 32.984521°E, 10.III.2017, (2♀, 1♂); Sovereign Base Area, Akrotiri, 34.600657°N, 32.971419°E, 10.III.2017, (1♀), collecting pollen on Asteraceae, all records S.P.M. Roberts leg., B. Tomozei det.

**Distribution.** Cyprus, Western Europe, Southern Europe, Eastern Europe, Northern Africa, Western Asia, Central Asia, Southern Asia (Iran).

**Notes.** Described from Cyprus as *Andrena batesiae* Cockerell, 1910, from Nicosia: 35.166667°N, 33.366667°E, D.M.A. Bate leg., T.D.A. Cockerell det. ♀, (NHMUK).

**Andrena (Micrandrena) lindbergella Pittioni, 1950**

**Type locality–country.** Cyprus, Mt. Troodos, Chionistra: 34.9364°N, 32.8636°E, 17.VI.1939, H. Lindberg leg., B. Pittioni det. ♀, (MZHF).

**References.**Pittioni (1950); Warncke (1967).

**Distribution.** Cyprus, Western Asia (Israel, Lebanon).

**Andrena (Truncandrena) medeninensis Pérez, 1895**

**References.**Warncke (1967, 1974).

**Distribution.** Cyprus, Southern Europe (Spain, Greece), Northern Africa, Western Europe (Turkey).

**Notes.** Records pertain to subspecies usura Warncke, 1967.

**Andrena (Simandrena) mehelyi Alfken, 1936**

**References.**Scheuchl and Willner (2016).

**Distribution.** Cyprus, Western Europe (Austria), Southern Europe, Eastern Europe, Western Asia (Turkey), Southern Asia (Iran).

**Andrena (Chrysandrena) merula Warncke, 1969**

**Type locality–country.** Cyprus, Ayia Eirini: 34.9816°N, 32.9712°E, 20.IV.1939, G.A. Mavromoustakis leg., K. Warncke det. ♂, (OLML).

**References.**Warncke (1969).

**Distribution.** Cyprus, Southern Europe (Greece), Western Asia (Turkey, Israel), Southern Asia (Afghanistan).

**Andrena (Cryptandrena) monacha Warncke, 1965**

**References.**Warncke (1965).

**Material examined.** Limassol District: Yermasoyia Dam, 34.755799°N, 33.096194°E, 7.III.2017, S.P.M. Roberts leg., B. Tomozei det. (1♂).

**Distribution.** Cyprus, Southern Europe (Greece), Western Europe (Turkey, Lebanon, Syria).

**Andrena (Melandrena) morio Brullé, 1832**

**References.** Mavromoustakis (1949 [“1948”]); Pittioni (1950); [Bibr B50][Bibr B52], 1954, 1957a); Warncke (1967); Georghiou (1977); Scheuchl and Willner (2016).

**Mavromoustakis localities.** Limassol, Pera Pedi, Trimiklini, Lania, Moni, Polemedia, Zakaki, Cherkes, Fasoula, Amathus, Potamitissa, Famagusta, Ayia Varvara (Stavrovouni).

**Material examined.** Nicosia District: Kakopetria, 34.992°N, 32.9082°E, 31.V.2012, 2.VI.2012, (1♀), pan trap (UAEG); Polemidia, 34.71178°N, 33.004775°E, 8.III.2017, (1♀); Famagusta District: Cape Greco, 34.963264°N, 34.066211°E, 15.III.2017, (7♂), all Kakopetria records S. Dimitriou leg., J. Devalez det., all Polemidia and Cape Greco records S.P.M. Roberts leg., B. Tomozei det.

**Distribution.** Cyprus, Western Europe (France, Austria), Southern Europe, Eastern Europe, Northern Africa, Western Asia, Central Asia.

**Notes.** Described from Cyprus as *Andrena morio athalassae* Pittioni, 1950, from Limassol: 34.66839°N, 33.03252°E, 4-6.VI.1939, H. Lindberg leg., B. Pittioni det., (MZHF).

**Andrena (Ulandrena) neocypriaca Mavromoustakis, 1956**

**Type locality–country.** Cyprus, Limassol: 34.66839°N, 33.03252°E, 16.IV.1949, G.A. Mavromoustakis leg., G.A. Mavromoustakis det. ♀, (DAAN).

**References.**Mavromoustakis (1956, 1957b); Warncke (1967).

**Mavromoustakis localities.** Near Limassol, Yermasoyia Hills.

**Material examined.** Limassol District: 2 km N of Anogyra, 34.748126°N, 32.732248°E, 1.V.2015, S.P.M. Roberts leg., B. Tomozei det. (1♀).

**Distribution.** Cyprus, Southern Europe (Greece), Western Asia (Turkey).

**Andrena (Melandrena) nigroaenea (Kirby, 1802)**

**References.** Mavromoustakis (1949 [“1948”], 1951, 1952, 1954, 1956); Georghiou (1977); Scheuchl and Willner (2016).

**Mavromoustakis localities.** Limassol, Polemedia Hills, Zakaki, Mesayitonia, Apsiou, Yermasoyia River, Amathus, Pissouri, Potamitissa, Trimiklini, Lania, Kitromili near Polemedia, Famagusta, Near Kyperounta, Larnaca.

**Material examined.** Nicosia District: Kakopetria, 34.992°N, 32.9082°E, 5-7.IV.2012, (1♀), pan trap (UAEG); Kakopetria, 34.992°N, 32.9082°E, 14-16.V.2012, (8♀), pan trap (UAEG); Limassol District: Amiantos, 34.918°N, 32.9472°E, 14-16.V.2012, (3♀), pan trap (UAEG); Almirolivado, 34.9333°N, 32.9004°E, 14-16.V.2012, (3♀), pan trap (UAEG); Makria Kontarka, 34.9095°N, 32.8971°E, 14-16.V.2012, (5♀), pan trap (UAEG); Troodos, Chionistra, 34.9317°N, 32.8664°E, 14-16.V.2012, (3♀), pan trap (UAEG); Almirolivado, 34.9333°N, 32.9004°E, 31.V.2012, 2.VI.2012, (5♀), pan trap (UAEG), all records S. Dimitriou leg., J. Devalez det.

**Distribution.** Cyprus, Widespread in Europe, Northern Africa, Western Asia, Southern Asia (Afghanistan, Iran).

**Andrena (Parandrenella) nisoria Warncke, 1969**

**References.**Warncke (1974).

**Material examined.** Limassol District: Yermasoyia Dam, 34.755799°N, 33.096194°E, 7.III.2017, S.P.M. Roberts leg., B. Tomozei det. (1♂).

**Distribution.** Cyprus, Western Asia, Southern Asia (Iran).

**Andrena (Plastandrena) oligotricha Mavromoustakis, 1952**

**Type locality–country.** Cyprus, Pera Pedi, 609m: 34.859444°N, 32.876111°E, 22.V.1929, G.A. Mavromoustakis leg., G.A. Mavromoustakis det. ♀, (DAAN).

**References.** Mavromoustakis (1949 [“1948”], 1952, 1953, 1954, 1957a); Warncke (1967).

**Mavromoustakis localities.** Limassol, Cherkes, Zakaki, Pera Pedi, Amathus, Lania, Kellaki, Ayia Varvara, Saettas, Potamitissa, Kato Amiantos, Ayios Kostantinos, Yerasa, Apsiou, Fasoula, Ayia Phyla, Amathus, Kykkos, Yermasoyia River.

**Material examined.** Paphos District: Nr Arminou Reservoir, 34.883435°N, 32.750988°E, 29.IV.2015, (2♀, 1♂), collecting pollen on Brassicaceae; Limassol District: 2 km N of Anogyra, 34.748126°N, 32.732248°E, 1.V.2015, (1♀), collecting pollen on Brassicaceae, all records S.P.M. Roberts leg., B. Tomozei det.

**Distribution.** Cyprus, Western Europe, Southern Europe, Northern Africa (Morocco, Algeria, Tunisia).

**Notes.** There has been considerable confusion over the true identity of this taxon. E. Scheuchl (2017, in litt.) regards *Andrena oligotricha* Mavromoustakis as a good species and that it replaces *Andrena bimaculata* in Cyprus. All previous records attributed to *A. bimaculata* are here treated as *A. oligotricha*.

**Andrena (Chlorandrena) orientana Warncke, 1965**

**References.**Schwenninger (2015).

**Material examined.** Limassol District: 2 km N of Anogyra, 34.748126°N, 32.732248°E, 1.V.2015, (1♀), collecting pollen on Asteraceae; Sovereign Base Area, Akrotiri, 34.600657°N, 32.971419°E, 10.III.2017, (5♀), collecting pollen on Asteraceae, all records S.P.M. Roberts leg., B. Tomozei det.

**Distribution.** Cyprus, Southern Europe (North Macedonia, Greece), Eastern Europe (Bulgaria, Ukraine), Western Asia (Turkey, Israel).

**Andrena (Taeniandrena) ovatula (Kirby, 1802)**

**References.** Mavromoustakis (1949 [“1948”], 1957b); Scheuchl and Willner (2016).

**Mavromoustakis localities.** Limassol, Amathus, Yermasoyia River.

**Material examined.** Limassol District: Yermasoyia Dam, 34.755799°N, 33.096194°E, 7.III.2017, S.P.M. Roberts leg., B. Tomozei det. (1♀, 1♂).

**Distribution.** Cyprus, Widespread in Europe, Northern Africa, Western Asia (Israel), Central Asia, Southern Asia (Iran, Afghanistan).

**Andrena (Chlorandrena) panurgimorpha Mavromoustakis, 1957**

**Type locality–country.** Cyprus, Limassol: 34.66839°N, 33.03252°E, 16.IV.1949, G.A. Mavromoustakis leg., G.A. Mavromoustakis det. ♀, (DAAN).

**References.**Mavromoustakis (1957b, 1958).

**Mavromoustakis localities.** Yermasoyia Hills.

**Material examined.** Limassol District: Anogyra to Pachna Road km 4, 34.764269°N, 32.757736°E, 5.V.2015, S.P.M. Roberts leg., B. Tomozei det. (1♀, 2♂).

**Distribution.** Cyprus, Southern Europe (Italy, Greece), Eastern Europe (Ukraine), Western Asia (Turkey, Israel), Southern Asia (Iran).

**Andrena (Truncandrena) pareklisiae Mavromoustakis, 1957**

**Type locality–country.** Cyprus, Lania: 34.82444°N, 32.920833°E, 11.IV.1953, G.A. Mavromoustakis leg., G.A. Mavromoustakis det. ♀, (DAAN).

**References.**Mavromoustakis (1957b); Warncke (1967).

**Distribution. Cyprus. ENDEMIC.**

**Andrena (Ulandrena) polemediana Mavromoustakis, 1956**

**Type locality–country.** Cyprus, Ayia Eirini (near Paramytha): 34.9816°N, 32.9712°E, 12.IV.1939, G.A. Mavromoustakis leg., G.A. Mavromoustakis det. ♀, (DAAN).

**References.**Mavromoustakis (1956, 1957b).

**Mavromoustakis localities.** Near Limassol.

**Material examined.** Limassol District: Anogyra, 34.741952°N, 32.734845°E, 3.V.2015, S.P.M. Roberts leg., B. Tomozei det. (1♀), visiting Asteraceae.

**Distribution. Cyprus. ENDEMIC.**

**Andrena (Melandrena) pyropygia Kriechbaumer, 1873**

**References.** Mavromoustakis (1949 [“1948”], 1951, 1952).

**Mavromoustakis localities.** Limassol, Polemedia Hills, Cherkes, Episkopi, Pano Kivides, Eleousa Monastery, Karpasian Peninsula, Apostolos Varnavas (near Famagusta).

**Distribution.** Cyprus, Southern Europe (Greece), Eastern Europe (Ukraine), Western Asia (Turkey, Israel), Southern Asia (Iran).

**Andrena (Poliandrena) pyrozonata Friese, 1921**

**References.**Mavromoustakis (1958); Warncke (1967).

**Distribution.** Cyprus, Western Asia (Turkey).

**Notes.** Described from Cyprus as *Andrena perapedica* Mavromoustakis, 1958, from Pera Pedi, 609 m: 34.859444°N, 32.876111°E, G.A. Mavromoustakis leg., G.A. Mavromoustakis det. ♀, (DAAN).

**Andrena (Truncandrena) paramythensis Mavromoustakis, 1957**

**References.**Mavromoustakis (1957a); Warncke (1967).

**Mavromoustakis localities.** Near Paramytha, Yerasa, Apsiou, Fasoula, Lania, Pera Pedi, Kellaki, Trimiklini.

**Distribution.** Cyprus, Western Asia (Turkey).

**Notes.** The subspecies described from Cyprus is *Andrena rufomaculata paramythensis* Mavromoustakis, 1957, from Paramytha vicinity (Vrisi tou Rotsou): 34.757°N, 32.972°E, 11.III.1944, G.A. Mavromoustakis leg., G.A. Mavromoustakis det. ♀, (DAAN).

**Andrena (Troandrena) saettana Warncke, 1975**

**Type locality–country.** Cyprus, Saittas: 34.8708333°N, 32.9166667°E, 2.V.1961, G.A. Mavromoustakis leg., K. Warncke det. ♀, (OLML).

**References.**Warncke (1975).

**Distribution.** Cyprus, Southern Europe (Greece), Western Asia (Turkey, Jordan).

**Andrena (Margandrena) sibthorpi Mavromoustakis, 1952**

**Type locality–country.** Cyprus, Polemedia Hills: 34.7134°N, 32.9812°E, 9.XII.1949, G.A. Mavromoustakis leg., G.A. Mavromoustakis det. ♀, (DAAN).

**References.**Mavromoustakis (1952); Warncke (1967).

**Mavromoustakis localities.** Polemedia Hills.

**Distribution. Cyprus. ENDEMIC.**

**Andrena (Taeniandrena) similis Smith, 1849**

**References.**Warncke (1975); Scheuchl and Willner (2016).

**Material examined.** Paphos District: Nr Arminou Reservoir, 34.883435°N, 32.750988°E, 29.IV.2015, (1♀); Limassol District: 0.5 km E of Vasa, 34.831384°N, 32.79744°E, 29.IV.2015, (1♀), visiting Asteraceae; Yermasoyia Dam, 34.755799°N, 33.096194°E, 7.III.2017, (1♀), all records S.P.M. Roberts leg., B. Tomozei det.

**Distribution.** Cyprus, Widespread in Europe, Western Asia, Central Asia, Southern Asia (Iran, Afghanistan), Eastern Asia (China).

**Notes.** Described from Cyprus as Andrena ocreata ssp. cyprisinaWarncke 1975, from Limassol: 34.66839°N, 33.03252°E, 8.III.1959, G.A. Mavromoustakis leg., K. Warncke det. ♀, (KW).

**Andrena (Micrandrena) spreta Pérez, 1895**

**References.**Warncke (1974, 1975).

**Distribution.** Cyprus, Western Europe, Southern Europe, Northern Africa, Western Asia, Southern Asia (Iran, Afghanistan).

**Notes.** Cyprus records pertain to subspecies scirpacea Warncke, 1975 (type locality: Turkey).

**Andrena (Aciandrena) tenuiformis Pittioni, 1950**

**Type locality–country.** Cyprus, Mt. Troodos, 1200–1952 m: 34.9364°N, 32.8636°E, 16-22.VI.1939, H. Lindberg leg., B. Pittioni det. ♀, (MZHF).

**References.**Pittioni (1950); Warncke (1967).

**Distribution.** Cyprus, Western Asia (Turkey, Israel).

**Andrena (Simandrena) thomsonii Ducke, 1898**

**References.**Gusenleitner and Schwarz (2002).

**Distribution.** Cyprus, Western Europe (France), Southern Europe, Western Asia (Turkey), Southern Asia (Iran).

**Andrena (Melandrena) thoracica (Fabricius, 1775)**

**References.** Mavromoustakis (1949 [“1948”], 1953, 1954, 1957a, b); Georghiou (1977); Scheuchl and Willner (2016).

**Mavromoustakis localities.** Limassol, Zakaki, Episkopi, Yermasoyia River, Famagusta.

**Distribution.** Cyprus, Widespread in Europe, Northern Africa (Morocco, Algeria, Tunisia), Western Asia, Central Asia, Southern Asia (Iran, Afghanistan), Eastern Asia (China, Korea).

**Notes.** Described from Cyprus as Andrena thoracica ssp. kotschyiMavromoustakis 1953, from Limassol: 34.66839°N, 33.03252°E, 12.V.1930, G.A. Mavromoustakis leg./det. ♀, (DAAN).

**Andrena (Cordandrena) torda Warncke, 1965**

**References.**Warncke (1965).

**Material examined.** Famagusta District: Cape Greco, 34.963264°N, 34.066211°E, 15.III.2017, S.P.M. Roberts leg., B. Tomozei det. (1♀).

**Distribution.** Cyprus, Southern Europe (Greece), Western Asia.

**Andrena (Simandrena) transitoria Morawitz, 1871**

**References.** Mavromoustakis (1949 [“1948”], 1951, 1952, 1953, 1954, 1957a).

**Mavromoustakis localities.** Limassol, Cherkes, Episkopi, Yermasoyia River, Ayia Phyla, Apsiou, Amathus, Pissouri, Potamitissa, Kato Amiandos, Fasoula, Yerasa, Moni, Trimiklini, Kellaki, Phinikaria River, Platania Forest.

**Distribution.** Cyprus, Southern Europe (North Macedonia, Greece), Eastern Europe, Western Asia, Central Asia (Kyrgyzstan, Tajikistan, Turkmenistan), Southern Asia (Afghanistan).

**Andrena (Troandrena) troodica Warncke, 1975**

**References.**Pittioni (1950).

**Distribution.** Cyprus, Western Asia (Turkey).

**Andrena (Truncandrena) truncatilabris Morawitz, 1877**

**References.** Mavromoustakis (1949 [“1948”], 1951, 1952, 1953, 1957a, b).

**Mavromoustakis localities.** Limassol, Cherkes, Apsiou, Fassouri, Yermasoyia River, Amathus, Pissouri, Yerasa, Near Paramytha, Trimiklini, Morphou, Near Famagusta.

**Material examined.** Limassol District: Yermasoyia Dam, 34.755799°N, 33.096194°E, 7.III.2017, (1♂, 2♀); Polemidia, 34.71178°N, 33.004775°E, 8.III.2017, (1♂, 1♀); Sovereign Base Area, Akrotiri, Bishop's Pool, 34.597305°N, 32.984521°E, 10.III.2017, (1♀); Paphos District: Nr Arminou Reservoir, 34.883435°N, 32.750988°E, 29.IV.2015, (1♂, 2♀), collecting pollen on *Sinapis* (Brassicaceae), all records S.P.M. Roberts leg., B. Tomozei det.

**Distribution.** Cyprus, Western Europe (France), Southern Europe, Eastern Europe, Northern Africa (Algeria, Libya), Western Asia (Israel, Lebanon, Syria), Central Asia (Kazakhstan, Turkmenistan), Southern Asia (Iran).

**Andrena (Notandrena) ungeri Mavromoustakis, 1952**

**Type locality–country.** Cyprus, Zakaki: 34.663951°N, 32.999785°E, 18.I.1949 [“1948”], G.A. Mavromoustakis leg., G.A. Mavromoustakis det. ♂, (DAAN).

**References.**Pittioni (1950); Mavromoustakis (1952).

**Distribution.** Cyprus, Southern Europe (North Macedonia, Greece), Eastern Europe (Hungary, Romania, Bulgaria), Northern Africa (Morocco), Western Asia (Turkey).

**Andrena (Notandrena) urdula Warncke, 1965**

**References.**Gusenleitner and Schwarz (2002).

**Distribution.** Cyprus, Southern Europe (Spain - only in the west, Greece), Eastern Europe (Romania, Bulgaria), Northern Africa (South Morocco), Western Asia.

**Andrena (Holandrena) variabilis Smith, 1853**

**References.** Mavromoustakis (1949 [“1948”], 1951).

**Mavromoustakis localities.** Limassol, Cherkes, Yermasoyia River, Near Zakaki, Near Enkomi of Famagusta.

**Material examined.** Limassol District: Almirolivado, 34.9333°N, 32.9004°E, 31.V.2012, 2.VI.2012, S. Dimitriou leg., E. Scheuchl det. (1♂), pan trap (UAEG); Anogyra to Pachna Road km 4, 34.764269°N, 32.757736°E, 5.V.2015, S.P.M. Roberts leg., B. Tomozei det. (1♀).

**Distribution.** Cyprus, Widespread in Europe, Northern Africa (Morocco, Algeria, Tunisia), Western Asia, Central Asia (Kazakhstan, west Turkmenistan), Southern Asia (Iran).

**Andrena (Cryptandrena) ventricosa Dours, 1873**

**References.** Mavromoustakis (1949 [“1948”], 1951, 1957a); Warncke (1975).

**Mavromoustakis localities.** Cherkes, Amathus, Pissouri, Pyrgos, Ayia Phyla, Paramytha.

**Distribution.** Cyprus, Western Europe (France, Austria), Southern Europe, Eastern Europe, Northern Africa (Morocco, Tunisia), Western Asia, Central Asia.

**Notes.** Described from Cyprus as *Andrena ventricosa ridibundus*Warncke 1975, from Cherkes: 34.65°N, 32.975°E, 11.III.1951, G.A. Mavromoustakis leg., K. Warncke det. ♀, (KW).

**Andrena (Ptilandrena) vetula Lepeletier, 1841**

**References.** Mavromoustakis (1949 [“1948”], 1951, 1954, 1958 ["1957"]); Georghiou (1977).

**Mavromoustakis localities.** Limassol, Fassouri, Yermasoyia River, Pera Pedi, Amathus, Near Famagusta.

**Material examined.** Limassol District: Anogyra, 34.73663°N, 32.732715°E, 26.IV.2015, (1♂, 1♀), collecting pollen on *Sinapis* (Brassicaceae); Anogyra, 34.741952°N, 32.734845°E, 3.V.2015, (3♀), collecting pollen on *Sinapis* (Brassicaceae); Anogyra to Avdimou Road km 2, 34.723986°N, 32.736892°E, 3.V.2015, (1♀), collecting pollen on *Sinapis* (Brassicaceae); Anogyra to Pachna Road km 4, 34.764269°N, 32.757736°E, 5.V.2015, (1♀), collecting pollen on Brassicaceae; Sovereign Base Area, Paramali Bay, 34.661805°N, 32.804261°E, 10.III.2017, (1♂), visiting *Sinapis alba* (Brassicaceae); Paphos District: Nr Arminou Reservoir, 34.883435°N, 32.750988°E, 29.IV.2015, (1♂, 5♀), collecting pollen on *Sinapis* (Brassicaceae); Famagusta District: Cape Greco, 34.963264°N, 34.066211°E, 15.III.2017, (2♂), all records S.P.M. Roberts leg., B. Tomozei det.

**Distribution.** Cyprus, Western Europe (France), Southern Europe (Portugal, Spain, Italy), Northern Africa, Western Asia, Central Asia (Turkmenistan), Southern Asia (Iran).

**Andrena (Holandrena) wilhelmi Schuberth, 1995**

**References.**Schuberth (1995).

**Distribution.** Cyprus, Southern Europe (North Macedonia, Italy, Greece), Eastern Europe (Ukraine), Western Asia (Turkey, Israel).

The only known Cyprus records are listed as Paratypes in Schuberth (1995): Limassol, III.1932, 1m J. Schuberth det.; Salamis (north of Famagusta), 23.III.1971, K.M. Guichard leg., J. Schuberth det.

**Andrena (Taeniandrena) wilkella (Kirby, 1802)**

**References.**Warncke (1965); Scheuchl and Willner (2016).

**Distribution.** Cyprus, Widespread in Europe, Western Asia, Central Asia, Southern Asia (India), Eastern Asia (China).

**MELITTURGINI**

**Genus *Melitturga* LATREILLE, 1809**

1 species.

**Melitturga (Melitturga) syriaca Friese, 1899**

**References.** Mavromoustakis (1949 [“1948”], 1951, 1957a).

**Mavromoustakis localities.** Near Limassol, Mesayitonia, Near Palodia.

**Material examined.** Limassol District: Yermasoyia Dam, 34.755799°N, 33.096194°E, 7.III.2017, (5♀); Paphos District: N of Elia Bridge, 34.900977°N, 32.776759°E, 29.IV.2015, (3♂), all records S.P.M. Roberts leg./det.

**Distribution.** Cyprus, Eastern Europe (Bulgaria), Western Asia.

**PANURGINI**

**Genus *Panurginus* NYLANDER, 1848**

2 species.

***Panurginus lactipennis* Friese, 1897**

**References.**Pittioni (1950); Mavromoustakis (1952).

**Mavromoustakis localities.** Mt. Troodos.

**Distribution.** Cyprus, Southern Europe (Greece), Eastern Europe (Romania), Western Asia (Turkey).

***Panurginus turcomanicus* Popov, 1936**

**References.**Warncke (1972b).

**Distribution.** Cyprus, Eastern Europe (Ukraine), Western Asia (Turkey, Israel, Azerbaijan), Central Asia.

**HALICTIDAE**

**HALICTINI**

**Genus *Halictus* LATREILLE, 1804**

14 species.

**Halictus (Halictus) asperulus Pérez, 1895**

**References.** Mavromoustakis (1949 [“1948”]); Pittioni (1950); Mavromoustakis (1951, 1952, 1953, 1957a); Ebmer (2014).

**Mavromoustakis localities.** Pera Pedi, Mt. Troodos, Platania Forest Station, Ayia Varvara (Stavrovouni), Eftagonia, Trimiklini, Pyrga, Xerokolimbi Stream near Trooditissa, Platres.

**Material examined.** Paphos District: 15 km SE Paphos, Kouklia, 34.72°N, 32.55°E, 20.VI.2013, C. Schmid-Egger leg., A.W. Ebmer det. (1♀).

**Distribution.** Cyprus, Western Europe (France, Austria), Southern Europe (Spain, Italy, Albania), Eastern Europe, Western Asia (Israel), Southern Asia (Iran).

**Halictus (Halictus) brunnescens (Eversmann, 1852)**

**References.** Mavromoustakis (1949 [“1948”]); Pittioni (1950); Mavromoustakis (1951, 1952, 1957a,b); Ebmer (2014).

**Mavromoustakis localities.** Limassol, Polemedia Hills, Akrotiri village, Cherkes, Episkopi, Yermasoyia River, Fassouri, Near Zakaki, Ayios Athanasios, Amathus, Eftagonia, Trimiklini, Mt. Troodos Kannoures Springs, Younaros of Zakaki, Pyrga (Larnaca), Near Ayios Theodoros (Pitsillia), Larnaca.

**Material examined.** Nicosia District: Linou, 35.0755°N, 32.9164°E, 5-7.IV.2012, (1♀), pan trap (UAEG); Kakopetria, 34.992°N, 32.9082°E, 5-7.IV.2012, (3♀), pan trap (UAEG); Kakopetria, 34.992°N, 32.9082°E, 25-27.IV.2012, (1♀), pan trap (UAEG); Limassol District: Amiantos, 34.918°N, 32.9472°E, 31.V.2012, 2.VI.2012, (2♀), pan trap (UAEG); Almirolivado, 34.9333°N, 32.9004°E, 16-18.IX.2011, (1♀), pan trap (UAEG); SBA, 8 km S Limassol, Akrotiri (near Airbase), 34.60°N, 32.97°E, 20.VI.2013, (4♂); Paphos District: 15 km SE Paphos, Kouklia, 34.72°N, 32.55°E, 20.VI.2013, (10♂); 20 km NNW Paphos, Lara Beach, 34.94°N, 32.31°E, 20.VI.2013, (1♀, 5♂), all Linou, Kakopetria, Amiantos and Almirolivado records S. Dimitriou leg., J. Devalez det., all Akrotiri and Paphos district records C. Schmid-Egger leg., A.W. Ebmer det.

**Distribution.** Cyprus, Western Europe (Austria), Southern Europe (Spain, Greece), Eastern Europe, Northern Africa, Western Asia (Turkey), Central Asia, Southern Asia.

**Halictus (Seladonia) cephalicus Morawitz, 1874**

**References.** Mavromoustakis (1949 [“1948”]); Mavromoustakis (1951, 1952, 1957a); Ebmer (2014).

**Mavromoustakis localities.** Limassol, Polemedia Hills, Cherkes, Akrotiri Bay, Moni, Yermasoyia River, Kathikas, Amathus, Near Eftagonia.

**Material examined.** Paphos District: 15 km SE Paphos, Kouklia, 34.72°N, 32.55°E, 20.VI.2013, (20♀, 1♂); 20 km N Paphos, Kathikas, 34.90°N, 32.42°E, 20.VI.20153, (3♀); 6 km NE Polis, beach, 35.06°N, 32.46°E, 20.VI.2013, (2♀); 6 km W Polis, botanical garden, 35.03°N, 32.37°E, 20.VI.2013, (1♀); Polis, 35.053539°N, 32.351197°E, 30.X.2016, (9♂), visiting *Dittrichia viscosa* (Asteraceae); 8 km N Paphos, Mavrokolympos Reservoir, 34.85°N, 32.40°E, 20.VI.2013, (5♀, 1♂); Limassol District: Troodos, Mt. Olympos, 34.93°N, 32.86°E, 20.VI.2013, (1♀); 1 km E of Pissouri, 34.677579°N, 32.722066°E, 27.IV.2015, (1♀), visiting Brassicaceae; Anogyra to Pachna Road km 4, 34.764269°N, 32.757736°E, 5.V.2015, (2♀), collecting pollen on Brassicaceae; Sovereign Base Area, Akrotiri, 34.628771°N, 32.941031°E, 29.X.2016, (1♂, 1♀), visiting *Dittrichia viscosa* (Asteraceae), all Kouklia, Kathikas, NE Polis, Polis botanical garden, Mavrokolympos Reservoir and Troodos, Mt. Olympos records C. Schmid-Egger leg., A.W. Ebmer det., all Polis, Pissouri, Anogyra, and Akrotiri records S.P.M. Roberts leg., A. Pauly det.

**Distribution.** Cyprus, Southern Europe, Eastern Europe, Western Asia, Southern Asia (Iran).

**Halictus (Mucoreohalictus) cypricus Blüthgen, 1937**

**Type locality–country.** Cyprus, Limassol: 34.66839°N, 33.03252°E, 7.III.1931, G.A. Mavromoustakis leg., P.A.V. Blüthgen det. ♀, (MFNB).

**References.**Blüthgen (1937); Mavromoustakis (1949 [“1948”]); Pittioni (1950); Mavromoustakis (1951, 1952); Georghiou (1977); Ebmer (2014).

**Mavromoustakis localities.** Limassol, Polemedia Hills, Cherkes, Zakaki, Episkopi, Pera Pedi, Akrotiri Bay, Pissouri, Near Zakaki, Famagusta.

**Material examined.** Paphos District: 15 km SE Paphos, Kouklia, 34.72°N, 32.55°E, 20.VI.2013, C. Schmid-Egger leg., A.W. Ebmer det. (1♂).

**Distribution.** Cyprus, Western Asia (Turkey, Israel), Southern Asia (Iran).

**Halictus (Halictus) fatsensis Blüthgen, 1936**

**References.**Ebmer (2014).

**Distribution.** Cyprus, Northern Africa (Egypt), Western Asia (Turkey, Israel, Syria).

**Halictus (Halictus) graecus Blüthgen, 1933**

**References.**Pesenko (2005).

**Distribution.** Cyprus, Southern Europe (Greece, Croatia), Eastern Europe (Bulgaria, Ukraine), Western Asia (Turkey).

**Halictus (Halictus) nicosiae Blüthgen, 1923**

**Type locality–country.** Cyprus, Nicosia: 35.166667°N, 33.366667°E, Staudinger leg., P. A.V. Blüthgen det.

**References.**Blüthgen (1923); Mavromoustakis (1949 [“1948”]); Pittioni (1950); Mavromoustakis (1951, 1952, 1953, 1954); Ebmer (2014).

**Mavromoustakis localities.** Polemedia Hills, Apsiou, Pera Pedi, Near Pano Kivides, Near Amathus, Trimiklini, Saettas, Platres, Fasoula, Yerasa, Lania, Livadin of Cedars (Paphos Forest), Platania Forest Station, Karpasian Peninsula, Eleousa Monastery, Xerokolimbi Stream near Trooditissa, Kykkou Monastery.

Ebmer localities: Platres, Mochi, Lefkara, Coral Bay, Agios Nikolaos, Troodos-Pano Platres road, Moni Trooditissa, Caledonia Waterfall, Tripylos Cedar Valley, south of Mt. Olympus, Kannaviou, Arminou to Filousa, Agios Nikolaos to Mandria, Pano Panagia, Moni Trooditissa, north of Mt. Olympus, South of Kakopetria, East of Platania, 3 km north east Troodos, north of Platres.

**Material examined.** Paphos District: 15 km SE Paphos, Kouklia, 34.72°N, 32.55°E, 20.VI.2013, (2♀); 20 km N Paphos, Kathikas, 34.90°N, 32.42°E, 20.VI.2013, (1♀); 8 km N Paphos, Mavrokolympos Reservoir, 34.85°N, 32.40°E, 20.VI.2013, (2♀); Limassol District: Troodos, Mt. Olympos, 34.93°N, 32.86°E, 20.VI.2013, C. (2♀); 2 km N of Anogyra, 34.748126°N, 32.732248°E, 1.V.2015, (1♀); Anogyra to Avdimou Road km 2, 34.723986°N, 32.736892°E, 3.V.2015, (1♀), visiting Asteraceae; 0.7 km N of Anogyra, 34.745537°N, 32.73385°E, 3.V.2015, (1♀); Anogyra to Pachna Road km 4, 34.764269°N, 32.757736°E, 5.V.215, (1♀), all Kouklia, Kathikas, Mavrokolympos Reservoir and Troodos Mt. Olympos records C. Schmid-Egger leg., A.W. Ebmer det., all Anogyra records S.P.M. Roberts leg., A. Pauly det.

**Distribution. Cyprus. ENDEMIC.**

**Halictus (Seladonia) phryganicus (Pauly & Devalez, 2015)**

**References.** Mavromoustakis (1949 [“1948”]); Pittioni (1950); Mavromoustakis (1951, 1952, 1957a); Georghiou (1977); Ebmer (2014); Pauly (2015).

**Mavromoustakis localities.** Limassol, Polemedia Hills, Mt. Troodos Kannoures springs, Erimi, Chiflicoudia marshes, Amathus, Pissouri, Yermasoyia River/Hills, Moni, Pyrga, Near Mesayitonia, Alassa.

**Material examined.** Paphos District: 15 km SE Paphos, Kouklia, 34.72°N, 32.55°E, 20.VI.2013, (5♀); 6 km NE Polis, beach, 35.06°N, 32.46°E, 20.VI.2013, (4♀); 6 km W Polis, botanical garden, 35.03°N, 32.37°E, 20.VI.2013, (1♀); 8 km N Paphos, Mavrokolympos Reservoir, 34.85°N, 32.40°E, 20.VI.2013, (1♀, 1♂); Limassol District: SBA, 8 km S Limassol, Akrotiri (near Airbase), 34.60°N, 32.97°E, 20.VI.2013, (1♀, 1♂); Sovereign Base Area, Episkopi, Kensington Cliffs, 34.670772°N, 32.846923°E, 4.V.2015, (2♀), all Paphos district and 8 km S Limassol Akrotiri records C. Schmid-Egger leg., A. Pauly det., all Episkopi records S.P.M. Roberts leg., A. Pauly det.

**Distribution.** Cyprus, Southern Europe (Greece), Eastern Europe (Bulgaria), Western Asia (Turkey, Israel), Central Asia, Southern Asia (Iran).

**Notes.** All records of *Halictus smaragdulus* Vachal, 1895 from Cyprus are now attributable to this recently described species.

**Halictus (Mucoreohalictus) pollinosus Sichel, 1860**

**References.**Blüthgen (1937); Mavromoustakis (1949 [“1948”], 1951, 1954, 1957a); Ebmer (2014).

**Mavromoustakis localities.** Limassol, Pera Pedi, hills near Trimiklini, Amiandos, Moni, Mt. Troodos Kannoures Springs.

**Material examined.** Limassol District: Amiantos, 34.918°N, 32.9472°E, 31.V.2012, 2.VI.2012, (2♀), pan trap (UAEG); Anogyra to Pachna Road km 4, 34.764269°N, 32.757736°E, 5.V.2015, (2♀), visiting Asteraceae; Paphos District: 2.7 km SW of Acheleia, Potamos tis Ezouzas, 34.729004°N, 32.457544°E, 30.IV.2015, (1♀), collecting pollen at *Chrysanthemum* sp. (Asteraceae), all Amiantos records S. Dimitriou leg., J. Devalez and A. Pauly det., all Anogyra and Paphos district records S.P.M. Roberts leg., A. Pauly det.

**Distribution.** Cyprus, Western Europe (France, Germany, Austria), Southern Europe, Eastern Europe, Northern Africa, Western Asia (Turkey, Israel, Jordan), Central Asia, Southern Asia (Iran, Afghanistan, Pakistan).

**Notes.** The subspecies from Cyprus is *Halictus pollinosus limissicus* Blüthgen, 1937, from Limassol: 34.66839°N, 33.03252°E, 2.VI.1928, G.A. Mavromoustakis leg., P.A.V. Blüthgen det. ♂, (MFNB).

**Halictus (Vestitohalictus) pulvereus Morawitz, 1874**

**References.**Ebmer (2014).

**Material examined.** Paphos District: 20 km N Paphos, Kathikas, 34.90°N, 32.42°E, 20.VI.2013, C. Schmid-Egger leg., A.W. Ebmer det. (2♀).

**Distribution.** Cyprus, Southern Europe (Greece), Eastern Europe (Ukraine), Western Asia (Turkey), Central Asia (Turkmenistan, Uzbekistan), Southern Asia (Iran, Afghanistan), Eastern Asia.

**Halictus (Halictus) quadricinctus (Fabricius, 1776)**

**References.**Georghiou (1977).

**Material examined.** Limassol District: 1 km E of Pissouri, 34.677579°N, 32.722066°E, 27.IV.2015, (1♀), visiting Asteraceae; 2 km N of Anogyra, 34.748126°N, 32.732248°E, 1.V.2015, (1♀), visiting Asteraceae, all records S.P.M. Roberts leg., A. Pauly det.

**Distribution.** Cyprus, Western Europe, Southern Europe, Eastern Europe, Northern Europe (Denmark, Finland), Western Asia, Central Asia, Southern Asia (Iran, Afghanistan), Eastern Asia (China, Mongolia).

**Halictus (Halictus) resurgens Nurse, 1903**

**References.**Blüthgen (1923); Mavromoustakis (1949 [“1948”], 1951, 1952, 1953, 1954, 1957a, b); Georghiou (1977); Ebmer (2014).

**Mavromoustakis localities.** Limassol, Mesayitonia, Chiflicoudia marshes, Moni, Yermasoyia River, Amathus, Lania, Pera Pedi, Famagusta, Nicosia, Livadin of Cedars (Paphos Forest), Near Zakaki, Near Amathus, Near Ayios Theodoros (Pitsillias).

**Material examined.** Paphos District: 20 km NNW Paphos, Lara Beach, 34.94°N, 32.31°E, 20.VI.2013, (2♀); 8 km N Paphos, Mavrokolympos Reservoir, 34.85°N, 32.40°E, 20.VI.2013, C. Schmid-Egger leg., A.W. Ebmer det. (2♀, 2♂); Limassol District: Anogyra, 34.73663°N, 32.732715°E, 26.IV.2015, (4♀), visiting *Chrysanthemum* (Asteraceae); Sovereign Base Area, Avdimou Bay Cliffs, 34.656698°N, 32.773339°E, 27.IV.2015, (1♀); Sovereign Base Area, Akrotiri, 34.628771°N, 32.941031°E, 29.X.2016, (3♂, 1♀), visiting *Dittrichia viscosa* (Asteraceae); 2 km N of Anogyra, 34.748126°N, 32.732248°E, 1.V.2015, (1♀); 0.7 km N of Anogyra, 34.745537°N, 32.73385°E, 3.V.2015, (1♀); Larnaca District: Zygi, 34.731233°N, 33.343487°E, 28.X.2016, (1♂), visiting *Dittrichia viscosa* (Asteraceae), all Paphos district records C. Schmid-Egger leg., A.W. Ebmer det., all Limassol and Larnaca district records S.P.M. Roberts leg., A. Pauly det.

**Distribution.** Cyprus, Southern Europe, Eastern Europe, Northern Africa (Egypt), Western Asia (Turkey), Central Asia, Southern Asia, Eastern Asia (China).

**Halictus (Halictus) subsenilis Blüthgen, 1955**

**References.**Ebmer (1975, 2014).

**Distribution.** Cyprus, Western Asia (Israel).

**Halictus (Halictus) tetrazonianellus Strand, 1909**

**References.** Mavromoustakis (1949 [“1948”]); Pittioni (1950); Mavromoustakis (1951, 1952, 1953); Ebmer (2014).

**Mavromoustakis localities.** Limassol, Cherkes, Moni, Yermasoyia River, Near Paramytha, Near Zakaki, Near Ayios Theodoros (Pitsilia), Larnaca.

**Material examined.** Paphos District: 15 km SE Paphos, Kouklia, 34.72°N, 32.55°E, 20.VI.2013, (13♀); 20 km N Paphos, Kathikas, 34.90°N, 32.42°E, 20.VI.2013, (2♀); 20 km NNW Paphos, Lara Beach, 34.94°N, 32.31°E, 20.VI.2013, (4♀); Limassol District: 1 km E of Pissouri, 34.677579°N, 32.722066°E, 27.IV.2015, (1♀), all Paphos district records C. Schmid-Egger leg., A.W. Ebmer det., all Limassol district records S.P.M. Roberts leg., A. Pauly det.

**Distribution.** Cyprus, Southern Europe (Greece), Eastern Europe (Ukraine, Moldova, Russian Federation), Western Asia (Turkey), Central Asia (Turkmenistan), Southern Asia (Iran).

**Genus *Lasioglossum* CURTIS, 1833**

37 species.

**Lasioglossum (Lasioglossum) aegyptiellum (Strand, 1909)**

**References.** Mavromoustakis (1949 [“1948”]); Pittioni (1950); Mavromoustakis (1951, 1952, 1957a); Ebmer (2014).

**Mavromoustakis localities.** Limassol, Cherkes, Chiflicoudia marshes, Fassouri, Amathus, Moni, Yermasoyia Hills, Near Mesayitonia, Larnaca.

**Material examined.** Paphos District: 15 km SE Paphos, Kouklia, 34.72°N, 32.55°E, 20.VI.2013, C. Schmid-Egger leg., A.W. Ebmer det. (1♀).

**Distribution.** Cyprus, Southern Europe (Croatia, Greece), Eastern Europe (Bulgaria), Northern Africa (Libya, Egypt), Western Asia, Central Asia (Turkmenistan), Southern Asia (Iran), Eastern Asia (China).

**Lasioglossum (Dialictus) akroundicum (Blüthgen, 1937)**

**Type locality–country.** Cyprus, Akrounda: 34.768889°N, 33.079444°E, 24.IV.1931, G.A. Mavromoustakis leg., P.A.V. Blüthgen det. ♀, (MFNB).

**References.**Blüthgen (1937); Mavromoustakis (1949 [“1948”]); Pittioni (1950); Mavromoustakis (1951, 1952); Ebmer (2014).

**Mavromoustakis localities.** Pyrgos, Near Paramytha, Akrounda, Near Eftagonia, Mt. Troodos, Listovounos.

**Material examined.** Limassol District: Troodos, Chionistra, 34.9317°N, 32.8664°E, 31.V.2012, 2.VI.2012, S. Dimitriou leg., A. Ebmer det. (1♀), pan trap (UAEG); Troodos, Mt. Olympos, 34.93°N, 32.86°E, 20.VI.2013, C. Schmid-Egger leg., A.W. Ebmer det. (3♀).

**Distribution. Cyprus. ENDEMIC.**

**Lasioglossum (Sphecodogastra) anellum (Vachal, 1905)**

**References.** Mavromoustakis (1949 [“1948”]); Pittioni (1950); Mavromoustakis (1957a); Ebmer (2014).

**Mavromoustakis localities.** Limassol, Pera Pedi, Mt. Troodos, Amiantos, Ayia Varvara (Stavrovouni).

**Material examined.** Paphos District: 15 km SE Paphos, Kouklia, 34.72°N, 32.55°E, 20.VI.2013, (1♀); 20 km NNW Paphos, Lara Beach, 34.94°N, 32.31°E, 20.VI.2013, (1♀); 6 km NE Polis, beach, 35.06°N, 32.46°E, 20.VI.2013, (1♀); 8 km N Paphos, Mavrokolympos Reservoir, 34.85°N, 32.40°E, 20.VI.2013, (1♀, 2♂); Nr Arminou Reservoir, 34.883435°N, 32.750988°E, 29.IV.2015, (1♀); Asprokremmos Dam, 34.720825°N, 32.551994°E, 30.IV.2015, (1♀), collecting pollen at *Sisymbrium* (Brassicaceae); Limassol District: 1 km E of Pissouri, 34.677579°N, 32.722066°E, 27.IV.2015, (1♀), collecting pollen at Brassicaceae; Pissouri 2 km S, 34.654385°N, 32.717924°E, 30.x.2016, (2♂), visiting *Ceratonia siliqua* (Fabaceae); Larnaca District: Zygi, 34.731233°N, 33.343487°E, 28.X.2016, (1♂), visiting *Ceratonia siliqua* (Fabaceae); Zygi, 34.746277°N, 33.384472°E, 28.X.2016, (1♂), visiting *Ceratonia siliqua* (Fabaceae), all Kouklia, Lara beach, Polis and Mavrokolympos Reservoir records C. Schmid-Egger leg., A.W. Ebmer det., all Arminou Reservoir, Asprokremmos Dam, Limassol and Larnaca district records S.P.M. Roberts leg., A. Pauly det.

**Distribution.** Cyprus, Southern Europe, Eastern Europe (Bulgaria), Western Asia, Southern Asia (Iran).

**Lasioglossum (Lasioglossum) aphrodite Ebmer, 2014**

**Type locality–country.** Cyprus, Mt. Troodos, S of Troodos, Caledonia Waterfall, 1400–1500 m: 34.9045°N, 32.86849°E, 10.VII.1987, A.W. Ebmer leg., A.W. Ebmer det. ♂, (AWE).

**References.**Ebmer (2014).

**Material examined.** Limassol District: Troodos, Chionistra, 34.9317°N, 32.8664°E, 14-16.V.2012, (1♂, 2♀), pan trap (UAEG); Troodos, Chionistra, 34.9317°N, 32.8664°E, 31.V.2012, 2.VI.2012, (7♂, 34♀), pan trap (UAEG), all records S. Dimitriou leg., A. Ebmer det.

**Distribution. Cyprus. ENDEMIC.**

**Lasioglossum (Hemihalictus) clypeiferellum (Strand, 1909)**

**References.** Mavromoustakis (1949 [“1948”]); Ebmer (2014).

**Mavromoustakis localities.** Limassol.

**Distribution.** Cyprus, Southern Europe (North Macedonia, Greece), Northern Africa (Egypt), Western Asia (Israel), Central Asia, Southern Asia (Iran, Afghanistan), Eastern Asia (Mongolia).

**Lasioglossum (Hemihalictus) convexiusculum (Schenck, 1853)**

**References.** Mavromoustakis (1949 [“1948”]); Ebmer (2014).

**Mavromoustakis localities.** Mt. Troodos.

**Material examined.** Limassol District: Troodos, Mt. Olympos, 34.93°N, 32.86°E, 20.VI.2013, C. Schmid-Egger leg., A.W. Ebmer det. (6♀).

**Distribution.** Cyprus, Western Europe, Southern Europe (Spain, Italy, Greece), Eastern Europe (Czech Republic, Poland, Ukraine), Northern Europe (Lithuania), Western Asia (Turkey), Central Asia (Tajikistan), Southern Asia (Iran).

**Lasioglossum (Sphecodogastra) damascenum (Pérez, 1910)**

**References.**Mavromoustakis (1954, 1957a); Ebmer (2014).

**Mavromoustakis localities.** Pera Pedi, Yerasa.

**Material examined.** Limassol District: Sovereign Base Area, Episkopi, Kensington Cliffs, 34.670772°N, 32.846923°E, 4.V.2015, S.P.M. Roberts leg., A. Pauly det. (1♂).

**Distribution.** Cyprus, Southern Europe, Eastern Europe (Hungary, Romania, Bulgaria), Western Asia (Turkey, Israel, Syria).

**Lasioglossum (Hemihalictus) dolichocephalum (Blüthgen, 1923)**

**References.** Mavromoustakis (1949 [“1948”]); Ebmer (2014).

**Mavromoustakis localities.** Ayios Athanasios.

**Distribution.** Cyprus, Southern Europe (North Macedonia, Croatia, Greece), Western Asia (Turkey, Israel, Lebanon).

**Lasioglossum (Hemihalictus) elegans (Lepeletier, 1841)**

**References.** Mavromoustakis (1949 [“1948”], 1951); Ebmer (2014).

**Mavromoustakis localities.** Apostolos Varnavas (near Famagusta), Ayios Athanasios, Evdhimou River.

**Distribution.** Cyprus, Western Europe (France, Switzerland, Austria), Southern Europe, Eastern Europe (Hungary, Romania), Northern Africa (Algeria), Western Asia (Turkey, Israel, Georgia), Central Asia, Southern Asia (Iran).

**Lasioglossum (Sphecodogastra) epipygiale (Blüthgen, 1924)**

**References.**Ebmer (2014).

**Distribution.** Cyprus, Western Asia (Turkey), Central Asia, Southern Asia.

**Lasioglossum (Hemihalictus) erraticum (Blüthgen, 1931)**

**References.** Mavromoustakis (1949 [“1948”]); Ebmer (2014).

**Mavromoustakis localities.** Ayios Athanasios.

**Distribution.** Cyprus, Southern Europe (Greece), Western Asia (Turkey, Armenia).

**Lasioglossum (Hemihalictus) griseolum (Morawitz, 1872)**

**References.** Mavromoustakis (1949 [“1948”], 1957a); Pittioni (1950); Ebmer (2014).

**Mavromoustakis localities.** Limassol, Cherkes.

**Material examined.** Paphos District: 20 km NNW Paphos, Lara Beach, 34.94°N, 32.31°E, 20.VI.2013, (3♀); 6 km NE Polis, beach, 35.06°N, 32.46°E, 20.VI.2013, (1♀), all records C. Schmid-Egger leg., A.W. Ebmer det.

**Distribution.** Cyprus, Western Europe, Southern Europe, Eastern Europe (Bulgaria), Northern Africa (Morocco, Tunisia), Western Asia, Central Asia, Southern Asia (Afghanistan).

**Lasioglossum (Sphecodogastra) imbecillum Ebmer, 1974**

**References.**Ebmer (2014).

**Distribution.** Cyprus, Southern Europe (Greece), Eastern Europe (Bulgaria), Western Asia (Turkey, Jordan).

**Lasioglossum (Lasioglossum) kotschyi Ebmer, 1981**

**Type locality–country.** Described from Cyprus as *Halictus eurasicus torquillus* Warncke, 1982, from Mt. Troodos: 34.9045°N, 32.86849°E, 7.VII.1935, A.W. Ebmer det. ♂.

**References.**Ebmer (1981); Warncke (1982); Pittioni (1950); Ebmer (2014).

**Material examined.** Limassol District: Troodos, Chionistra, 34.9317°N, 32.8664°E, 14-16.V.2012, (14♀), pan trap (UAEG); Troodos, Chionistra, 34.9317°N, 32.8664°E, 31.V.2012, 2.VI.2012, (1♀), pan trap (UAEG), all records S. Dimitriou leg., A. Ebmer det.

**Distribution. Cyprus. ENDEMIC.**

**Lasioglossum (Hemihalictus) laevidorsum (Blüthgen, 1923)**

**References.**Blüthgen (1937); Mavromoustakis (1949 [“1948”], 1951); Warncke (1982); Ebmer (2014).

**Mavromoustakis localities.** Mt. Troodos Kannoures springs, Pera Pedi, Between Mozaras Station and Kato Aminados, Mt. Troodos Pasha Livadin.

**Distribution.** Cyprus, Western Europe (France, Switzerland, Austria), Southern Europe, Northern Africa (Egypt), Western Asia (Turkey, Israel), Southern Asia (Iran, India).

**Notes.** The subspecies described from Cyprus is *Lasioglossum laevidorsum troodicum* (Blüthgen, 1937), from Mt. Troodos: 34.9234°N, 32.8833°E, 19.VI.1935 G.A. Mavromoustakis leg., P.A.V. Blüthgen det. ♀, (KW).

**Lasioglossum (Sphecodogastra) laticeps (Schenck, 1870)**

**References.**Pittioni (1950); Mavromoustakis (1957b); Ebmer (2014).

**Mavromoustakis localities.** Limassol, Apsiou, Moni River, Near Amathus, Kaloiri Hill, Morphou, Trimiklini, Kellaki, Monagroulli, Near Mesayitonia, Polemedia.

**Distribution.** Cyprus, Widespread in Europe, Western Asia (Turkey, Israel), Southern Asia (Iran).

**Lasioglossum (Leuchalictus) leucozonium (Schrank, 1781)**

**References.**Blüthgen (1937); Mavromoustakis (1949 [“1948”], 1951, 1952, 1953); Ebmer (2014).

**Mavromoustakis localities.** Limassol, Polemedia Hills, Yermasoyia River, Amathus, Trimiklini, Near Famagusta, Saettas, Kilani.

**Material examined.** Nicosia District: Linou, 35.0755°N, 32.9164°E, 5-7.IV.2012, (7♀), pan trap (UAEG); Linou, 35.0755°N, 32.9164°E, 25-27.IV.2012, (6♀), pan trap (UAEG); Linou, 35.0755°N, 32.9164°E, 14-16.V.2012, (1♀), pan trap (UAEG); Kakopetria, 34.992°N, 32.9082°E, 5-7.IV.2012, (1♀), pan trap (UAEG); Kakopetria, 34.992°N, 32.9082°E, 14-16.V.2012, (1♀), pan trap (UAEG); Limassol District: Amiantos, 34.918°N, 32.9472°E, 5-7.IV.2012, (2♀), pan trap (UAEG), all records S. Dimitriou leg., J. Devalez det.

**Distribution.** Cyprus, Widespread in Europe, Northern Africa, Western Asia, Central Asia, Southern Asia (Iran), Eastern Asia (Mongolia, China).

**Lasioglossum (Hemihalictus) limbellum (Morawitz, 1876)**

**References.**Ebmer (2014).

**Distribution.** Cyprus, Western Europe, Southern Europe, Eastern Europe, Northern Africa (Morocco, Algeria, Tunisia), Western Asia (Turkey, Israel), Central Asia, Southern Asia (Iran, Afghanistan), Eastern Asia (China).

**Lasioglossum (Sphecodogastra) lineare (Schenck, 1870)**

**References.** Mavromoustakis (1949 [“1948”], 1952, 1954); Ebmer (2014).

**Mavromoustakis localities.** Limassol, Akrotiri Forest, Polemedia Hills, Ayios Athanasios, Yermasoyia River, Pera Pedi.

**Material examined.** Limassol District: Troodos, Chionistra, 34.9317°N, 32.8664°E, 14-16.V.2012, S. Dimitriou leg., A. Ebmer det. (1♂), pan trap (UAEG); Paphos District: 2.7 km SW of Acheleia, Potamos tis Ezousas, 34.729004°N, 32.457544°E, 30.IV.2015, S.P.M. Roberts leg., A. Pauly det. (1♀).

**Distribution.** Cyprus, Western Europe, Southern Europe, Eastern Europe, Western Asia, Central Asia (Turkmenistan, Tajikistan, Uzbekistan), Southern Asia (Iran).

**Lasioglossum (Hemihalictus) lucidulum (Schenck, 1861)**

**References.**Ebmer (2014).

**Material examined.** Paphos District: 15 km SE Paphos, Kouklia, 34.72°N, 32.55°E, 20.VI.2013, C. Schmid-Egger leg., A.W. Ebmer det. (1♀).

**Distribution.** Cyprus, Widespread in Europe, Northern Africa, Western Asia (Turkey, Israel, Georgia), Central Asia, Southern Asia (Iran, Afghanistan, Pakistan), Eastern Asia.

**Lasioglossum (Sphecodogastra) malachurum (Kirby, 1802)**

**References.** Mavromoustakis (1949 [“1948”]); Pittioni (1950); Mavromoustakis (1951, 1952, 1953, 1954, 1957a, b); Georghiou (1977); Ebmer (2014).

**Mavromoustakis localities.** Limassol, Polemedia Hills, Yermasoyia, Cherkes, Episkopi, Chiflicoudia marshes, Famagusta.

**Material examined.** Paphos District: 15 km SE Paphos, Kouklia, 34.72°N, 32.55°E, 20.VI.2013, (1♂); 20 km NNW Paphos, Lara Beach, 34.94°N, 32.31°E, 20.VI.2013, (6♀); 2.7 km SW of Acheleia, Potamos tis Ezouzas, 34.729004°N, 32.457544°E, 30.IV.2015, (1♀); Famagusta District: Rizokarpaso, 5 km E, 35.63°N, 34.50°E, 10.IV.2007, (1♂); Limassol District: Sovereign Base Area, Akrotiri, Bishop's Pool, 34.597305°N, 32.984521°E, 28.IV.2015, (1♀), all Kouklia, Lara Beach, and Rizokarpaso records C. Schmid-Egger leg., A.W. Ebmer det., all Potamos tis Ezousas and Akrotiri records S.P.M. Roberts leg., A. Pauly det.

**Distribution.** Cyprus, Widespread in Europe, Northern Africa, Western Asia, Central Asia (Turkmenistan), Southern Asia (Iran).

**Lasioglossum (Dialictus) mandibulare (Morawitz, 1866)**

**References.** Mavromoustakis (1949 [“1948”], 1952, 1957a); Ebmer (2014).

**Mavromoustakis localities.** Limassol, Cherkes, Pernera coast of Paralimni, Chiflicoudia marshes, Famagusta.

**Distribution.** Cyprus, Western Europe (Switzerland), Southern Europe (Spain, Bosnia and Herzegovina, Greece), Eastern Europe (Romania, Bulgaria, Russian Federation), Western Asia.

**Lasioglossum (Evylaeus) marginatum (Brullé, 1832)**

**References.** Mavromoustakis (1949 [“1948”], 1951, 1952, 1953, 1954); Ebmer (2014).

**Mavromoustakis localities.** Polemedia Hills, Ayia Phyla, Pera Pedi, Amathus, Trimiklini, Kellaki, Lania, Platania Forest Station, Amyrou Monastery, Ayia Irini Station.

**Material examined.** Kyrenia District: 15 km E Kyrenia, 'Turtle Beach', 35.334413°N, 33.494187°E, 10.IV.2007, (15♀); Limassol District: Anogyra, 34.73663°N, 32.732715°E, 26.IV.2015, (1♀); Sovereign Base Area, Avdimou Bay Cliffs, 34.656698°N, 32.773339°E, 27.IV.2015, (1♀); 2 km N of Anogyra, 34.748126°N, 32.732248°E, 1.V.2015, (4♀); 0.7 km N of Anogyra, 34.745537°N, 32.73385°E, 3.V.2015, (1♀), visiting *Cistus creticus* (Cistaceae); Anogyra to Pachna Road km 4, 34.764269°N, 32.757736°E, 5.V.2015, (1♀), collecting pollen on Brassicaceae; Paphos District: Nr Arminou Reservoir, 34.883435°N, 32.750988°E, 29.IV.2015, (5♀); 2.7 km SW of Acheleia, Potamos tis Ezouzas, 34.729004°N, 32.457544°E, 30.IV.2015, (1♀), all Kyrenia district records C. Schmid-Egger leg., A.W. Ebmer det., all Limassol and Paphos district records S.P.M. Roberts leg., A. Pauly det.

**Distribution.** Cyprus, Western Asia, Southern Europe (Spain, Greece), Eastern Europe (Czech Republic, Hungary, Russian Federation), Western Asia (Israel, Armenia), Central Asia, Southern Asia.

**Lasioglossum (Hemihalictus) medinai (Vachal, 1895)**

**References.** Mavromoustakis (1949 [“1948”], 1954); Ebmer (2014) [all as *L. villosulum* (Kirby).

Integrative taxonomy (Pauly et al. 2019) resuscitated two cryptic species in this complex including *L. medinai*, the form verified to occur on Cyprus, whereas *Lasioglossum villosulum villosulumsensu stricto* has been confirmed from Greece including Crete and from Israel but not from Cyprus.

**References.** Mavromoustakis (1949 [“1948”], 1954); Ebmer (2014).

**Distribution.** Western Europe (Austria), Southern Europe (Spain, France, Italy, Greece including Crete), Eastern Europe (Romania, Russia (Volgograd)), Northern Africa (Algeria), Western Asia.

**Lasioglossum (Hemihalictus) mesosclerum (Pérez, 1903)**

**References.**Ebmer (2014).

**Material examined.** Paphos District: 15 km SE Paphos, Kouklia, 34.72°N, 32.55°E, 20.VI.2013, (1♀); 20 km NNW Paphos, Lara Beach, 34.94°N, 32.31°E, 20.VI.2013, (1♀); 8 km N Paphos, Mavrokolympos Reservoir, 34.85°N, 32.40°E, 20.VI.2013, (1♂), all records C. Schmid-Egger leg., A.W. Ebmer det.

**Distribution.** Cyprus, Western Asia (France, Austria), Southern Europe, Eastern Europe, Northern Africa (Morocco, Libya, Egypt), Western Asia, Central Asia, Southern Asia (Iran, Afghanistan).

**Lasioglossum (Hemihalictus) minutissimum (Kirby, 1802)**

**References.**Ebmer (2014).

**Distribution.** Cyprus, Widespread in Europe, Northern Africa, Western Asia (Turkey), Southern Asia (Pakistan).

**Lasioglossum (Hemihalictus) nitidiusculum (Kirby, 1802)**

**References.**Scheuchl and Willner (2016).

**Distribution.** Cyprus, Widespread in Europe, Northern Africa (Morocco, Algeria, Tunisia), Western Asia (Turkey), Central Asia (Turkmenistan, Kazakhstan, Tajikistan), Southern Asia (Iran).

**Lasioglossum (Sphecodogastra) obscuratum (Morawitz, 1876)**

**References.** Mavromoustakis (1949 [“1948”], 1951, 1952, 1954, 1958 ["1957"]).

**Mavromoustakis localities.** Polemedia Hills, Yermasoyia River, Episkopi, Amathus, Fasoula, Yerasa, Mt. Troodos Kannoures Springs, Mt. Troodos open slopes, Amiantos, Near Paramytha, Lania, Kykkou Monastery, Famagusta.

**Material examined.** Limassol District: Troodos, Chionistra, 34.9317°N, 32.8664°E, 14-16.V.2012, (1♂, 1♀), pan trap (UAEG); Troodos, Chionistra, 34.9317°N, 32.8664°E, 31.V.2012, 2.VI.2012, (2♂, 4♀), pan trap (UAEG); Paphos District: 20 km NNW Paphos, Lara Beach, 34.94°N, 32.31°E, 20.VI.2013, (1♀), all Limassol district records S. Dimitriou leg., J. Devalez det., all Paphos district records C. Schmid-Egger leg., A.W. Ebmer det.

**Distribution.** Cyprus, Western Europe (Austria), Southern Europe (Croatia, Greece), Eastern Europe, Western Asia, Central Asia, Southern Asia (Iran, Afghanistan).

**Lasioglossum (Lasioglossum) pallens (Brullé, 1832)**

**References.**Warncke (1982); Ebmer (2014).

**Material examined.** Nicosia District: Kakopetria, 34.992°N, 32.9082°E, 25-27.IV.2012, S. Dimitriou leg., A. Ebmer det. (1♀), pan trap (UAEG); Limassol District: 2 km N of Anogyra, 34.748126°N, 32.732248°E, 1.V.2015, S.P.M. Roberts leg., A. Pauly det. (2♀).

**Distribution.** Cyprus, Western Europe, Southern Europe (Spain, Italy, Greece), Northern Europe (Lithuania), Eastern Europe (Czech Republic, Hungary), Northern Africa (Morocco), Western Asia (Turkey), Southern Asia (Iran), Eastern Asia (Mongolia).

**Notes.** The subspecies described from Cyprus is *Lasioglossum pallens kantarae* (Warncke, 1982), from Kantara Castle: 35.4064°N, 33.9233°E, 7.IV.1953, K. Warncke det. ♂, (KW).

**Lasioglossum (Sphecodogastra) pauxillum (Schenck, 1853)**

**References.**Pittioni (1950); Ebmer (2014).

**Distribution.** Cyprus, Widespread in Europe, Northern Africa (Morocco to Tunisia), Western Asia, Central Asia, Southern Asia (Iran).

**Lasioglossum (Dialictus) podolicum (Noskiewicz, 1925)**

**References.**Ebmer (2014).

**Distribution.** Cyprus, Western Europe (France, Switzerland), Southern Europe, Eastern Europe (Hungary, Ukraine), Western Asia (Turkey), Southern Asia (Iran).

**Lasioglossum (Hemihalictus) puncticolle (Morawitz, 1872)**

**References.**Ebmer (2014).

**Distribution.** Cyprus, Western Europe, Southern Europe, Northern Europe (United Kingdom), Eastern Europe, Northern Africa, Western Asia (Turkey, Israel, Georgia), Southern Asia (Iran).

**Lasioglossum (Hemihalictus) pygmaeum (Schenck, 1853)**

**References.**Ebmer (2014).

**Material examined.** Kyrenia District: 15 km E Kyrenia, 'Turtle Beach', 35.334413°N, 33.494187°E, 10.IV.2007, (1♀); Limassol District: 2 km N of Anogyra, 34.748126°N, 32.732248°E, 1.V.2015, (1♀), collecting pollen at *Cistus creticus* (Cistaceae); Sovereign Base Area, Episkopi Kensington Cliffs, 34.670772°N, 32.846923°E, 4.V.2015, (3♂); Anogyra to Pachna Road km 4, 34.764269°N, 32.757736°E, 5.V.2015, (1♀), all Kyrenia district records C. Schmid-Egger leg., A.W. Ebmer det., all Limassol district records S.P.M. Roberts leg., A. Pauly det.

**Distribution.** Cyprus, Western Europe, Southern Europe, Eastern Europe, Northern Africa (Morocco to Tunisia), Western Asia, Central Asia (Turkmenistan, Kazakhstan, Tajikistan), Southern Asia.

**Lasioglossum (Hemihalictus) transitorium (Schenck, 1870)**

**References.**Erlandsson (1979).

**Material examined.** Kyrenia District: 15 km E Kyrenia, 'Turtle Beach', 35.334413°N, 33.494187°E, 10.IV.2007, (1♀); Limassol District: Anogyra to Avdimou Road km 2, 34.723986°N, 32.736892°E, 3.V.2015, (1♂, 1♀), visiting Apiaceae; Sovereign Base Area, Episkopi, Kensington Cliffs, 34.670772°N, 32.846923°E, 4.V.2015, (2♀), all Kyrenia district records C. Schmid-Egger leg., A.W. Ebmer det., all Limassol district records S.P.M. Roberts leg., A. Pauly det.

**Distribution.** Cyprus, Western Europe (France), Southern Europe, Eastern Europe (Bulgaria).

**Lasioglossum (Sphecodogastra) tricinctum (Schenck, 1874)**

**References.**Ebmer (2014).

**Material examined.** Famagusta District: 15 km E Rizokarpaso, 'Golden Sands', 35.64°N, 34.55°E, 9.VIII.2001, (1♀); Paphos District: 20 km N Paphos, Kathikas, 34.90°N, 32.42°E, 20.VI.2013, (1♂); 20 km NNW Paphos, Lara Beach, 34.94°N, 32.31°E, 20.VI.2013, C. Schmid-Egger leg., A.W. Ebmer det. (1♀); 8 km N Paphos, Mavrokolympos Reservoir, 34.85°N, 32.40°E, 20.VI.2013, (1♀, 1♂), all records C. Schmid-Egger leg., A.W. Ebmer det.

**Distribution.** Cyprus, Western Europe, Southern Europe, Eastern Europe (Hungary, Poland, Ukraine), Western Asia (Turkey, Azerbaijan), Southern Asia (Iran).

**Notes.** The subspecies described from Cyprus is *Lasioglossum tricinctum lonicerae* Ebmer, 2014, from Mt. Troodos: 34.9234°N, 32.8833°E, 11.VII.1987, A.W. Ebmer leg., A.W. Ebmer det. ♂, (AWE).

**Lasioglossum (Hemihalictus) truncaticolle (Morawitz, 1877)**

**References.**Ebmer (2014).

**Distribution.** Cyprus, Western Europe (France), Southern Europe, Eastern Europe, Northern Africa (Algeria), Western Asia (Israel), Central Asia (Kazakhstan), Southern Asia (Iran).

**Lasioglossum (Leuchalictus) zonulum (Smith, 1848)**

**References.**Ebmer (2014).

**Distribution.** Cyprus, Widespread in Europe, Western Asia (Turkey, Azerbaijan), Southern Asia (Iran), Eastern Asia (China).

**Genus *Sphecodes* LATREILLE, 1804**

14 species.

***Sphecodes albilabris* (Fabricius, 1793)**

**References.**Pittioni (1950); Mavromoustakis (1951, 1952, 1953, 1957a) (Warncke, 1992).

**Mavromoustakis localities.** Cherkes, Moni River, Near Limassol, Nicosia, Polemedia Hills, Salipes marshes near Akrotiri, Trimiklini, Yermasoyia River, Zakaki marshes.

**Distribution.** Cyprus, Widespread in Europe, Northern Africa, Western Asia, Central Asia, Southern Asia (Iran, India).

**Notes.** Includes specimens labelled *S. fumipennis* by Mavromoustakis, which are referable to this species. According to Warncke (1992) Cyprus specimens refer to *S. a. rubripes* Spinola, 1838, variously treated by recent authors as a valid subspecies or as a species.

***Sphecodes alternatus* Smith, 1853**

**References.** Mavromoustakis (1949 [“1948”]); Pittioni (1950); Mavromoustakis (1952, 1954).

**Mavromoustakis localities.** Asomatos, Pera Pedi, Akrounda.

**Distribution.** Cyprus, Western Europe (France, Austria), Southern Europe, Eastern Europe, Northern Africa, Western Asia, Central Asia, Southern Asia (Iran), Eastern Asia (China).

**Notes.** Described from Cyprus as *Sphecodes alternatus lindbergi* Pittioni, 1950, from Geroskipou, E of Paphos: 34.76666°N, 32.46666°E, 20.VII.1939, H. Lindberg leg., B. Pittioni det., (MZHF).

***Sphecodes croaticus* Meyer, 1922**

**References.**Blüthgen (1937); Mavromoustakis (1949 [“1948”], 1952).

**Mavromoustakis localities.** Limassol, Episkopi, Yermasoyia River.

**Distribution.** Cyprus, Western Europe, Southern Europe, Eastern Europe, Northern Africa (Morocco), Western Asia (Turkey), Central Asia (Turkmenistan).

**Notes.** The subspecies described from Cyprus is *Sphecodes croaticus cypricus* Blüthgen, 1937, from Limassol: 34.66839°N, 33.03252°E, 7.III.1931, G.A. Mavromoustakis leg., P.A.V. Blüthgen det. ♀, (MFNB).

***Sphecodes ephippius* (Linnaeus, 1767)**

**Material examined.** Limassol District: Yermasoyia Dam, 34.755799°N, 33.096194°E, 7.III.2017, Bee Course students leg., M. Schwarz det. (1♀).

**Distribution.** Cyprus, Widespread in Europe, Northern Africa, Western Asia (Turkey), Eastern Asia.

***Sphecodes gibbus* (Linnaeus, 1758)**

**References.**Blüthgen (1937); Mavromoustakis (1949 [“1948”]); Pittioni (1950); Mavromoustakis (1952, 1954, 1957a); Georghiou (1977).

**Mavromoustakis localities.** Limassol, Episkopi, Pera Pedi, Akrounda, Moni River, Younarka (near Zakaki), Near Asomatos.

**Distribution.** Cyprus, Widespread in Europe, Northern Africa, Western Asia, Central Asia, Southern Asia, Eastern Asia (China, Mongolia).

**Notes.** Described from Cyprus as *Sphecodes pergibbus* Blüthgen, 1937, from [Limassol; Episcopi]: 34.675134°N, 32.882598°E, IV-VI, [G.A. Mavromoustakis] leg., P.A.V. Blüthgen, det., (MFNB).

***Sphecodes longuloides* Blüthgen, 1923**

**References.**Warncke (1992).

**Distribution.** Cyprus, Southern Europe (Spain, Italy, Portugal), Northern Africa (Morocco, Algeria, Tunisia).

***Sphecodes longulus* Hagens, 1882**

**References.**Scheuchl and Willner (2016).

**Distribution.** Cyprus, Widespread in Europe, Northern Africa (Tunisia, Libya), Western Asia (Turkey, Syria, Armenia), Central Asia, Southern Asia (Iran), Eastern Asia (China, Japan).

***Sphecodes monilicornis* (Kirby, 1802)**

**References.** Mavromoustakis (1949 [“1948”], 1952, 1957a); Pittioni (1950).

**Mavromoustakis localities.** Limassol, Fassouri, Mt. Troodos, Cherkes, Moni River, Pyrga (Larnaca).

**Material examined.** Limassol District: 1 km E of Pissouri, 34.677579°N, 32.722066°E, 27.IV.2015, S.P.M. Roberts leg./det. (2♀).

**Distribution.** Cyprus, Widespread in Europe, Northern Africa, Western Asia, Central Asia (Turkmenistan, Uzbekistan, Tajikistan), Southern Asia (Iran, Pakistan, India), Eastern Asia.

**Notes.** Described from Cyprus as *Sphecodes quadratus cephalotiformes* Pittioni, 1950, from Mt. Troodos, Chionistra: 34.9364°N, 32.8636°E, 17.VI.1939, H. Lindberg leg., B. Pittioni det., (MZHF). *Sphecodes monilicornis* is recognised *sensu lato* following Nieto et al. (2014), including forms that have been considered distinct such as *Sphecodes cephalotes*.

***Sphecodes pellucidus* Smith, 1845**

**References.** Mavromoustakis (1949 [“1948”], 1952).

**Mavromoustakis localities.** Episkopi, Pera Pedi, Ayios Kostantinos of Pitsilia.

**Distribution.** Cyprus, Widespread in Europe, Northern Africa, Western Asia, Central Asia (Turkmenistan, Kyrgyzstan), Southern Asia (Iran), Eastern Asia (China, Mongolia).

***Sphecodes pinguiculus* Pérez, 1903**

**References.**Warncke (1992).

**Distribution.** Cyprus, Western Europe (France), Southern Europe (Spain, Italy, Greece), Eastern Europe, Northern Africa, Western Asia (Turkey), Eastern Asia (Mongolia).

***Sphecodes pseudofasciatus* Blüthgen, 1925**

**Material examined.** Limassol District: Yermasoyia Dam, 34.755799°N, 33.096194°E, 7.III.2017, Bee Course students leg., M. Schwarz det. (2♀).

**Distribution.** Cyprus, Western Europe, Southern Europe (Spain, Italy. Portugal), Eastern Europe, Western Asia (Turkey).

***Sphecodes puncticeps* Thomson, 1870**

**References.** Mavromoustakis (1949 [“1948”], 1952).

**Mavromoustakis localities.** Cherkes, Yermasoyia River.

**Distribution.** Cyprus, Widespread in Europe, Northern Africa (Egypt), Western Asia (Israel), Central Asia (Uzbekistan, Tajikistan), Southern Asia (Iran), Eastern Asia (Mongolia).

***Sphecodes rufiventris* (Panzer, 1798)**

**References.** Mavromoustakis (1949 [“1948”], 1952).

**Mavromoustakis localities.** Episkopi, Pera Pedi.

**Distribution.** Cyprus, Widespread in Europe, Northern Africa (Morocco, Algeria), Western Asia, Central Asia (Uzbekistan, Kazakhstan, Tajikistan), Southern Asia (Iran).

***Sphecodes schenckii* Hagens, 1882**

**References.**Warncke (1992).

**Distribution.** Cyprus, Western Asia, Southern Europe, Eastern Europe, Northern Africa, Western Asia (Turkey), Southern Asia (Iran).

**NOMIINI**

**Genus *Pseudapis* KIRBY, 1900**

4 species.

**Pseudapis (Nomiapis) bispinosa (Brullé, 1832)**

**References.** Mavromoustakis (1949 [“1948”]); Pittioni (1950); Mavromoustakis (1951, 1952, 1957a); Ebmer (2014).

**Mavromoustakis localities.** Limassol, Akrotiri Forest, Cherkes, Zakaki, Near Akrotiri, Moni, Pyrgos, Larnaca, Eftagonia.

**Distribution.** Cyprus, Western Europe (France), Southern Europe, Eastern Europe, Northern Africa, Western Asia, Central Asia, Southern Asia, Eastern Asia (China).

**Pseudapis (Nomiapis) diversipes (Latreille, 1806)**

**References.** Mavromoustakis (1948); Pittioni (1950); Mavromoustakis (1951, 1952, 1957a); Ebmer (2014).

**Mavromoustakis localities.** Limassol, Cherkes, Akrotiri Bay, Yermasoyia River, Moni, Tsada, Erimi, Pera Pedi, Chiflicoudia marshes, Near Akhyritou, Kathikas, Near Enkomi of Famagusta, Larnaca, Ayia Varvara Stavrovouni.

**Material examined.** Famagusta District: Achna Dam, 35.05519°N, 33.814011°E, 28.X.2016, (1♀), visiting *Dittrichia viscosa* (Asteraceae); Paphos District: Polis, 35.053539°N, 32.351197°E, 30.X.2016, (2♂, 1♀), visiting *Dittrichia viscosa* (Asteraceae), all records S.P.M. Roberts leg., A. Pauly det.

**Distribution.** Cyprus, Western Europe, Southern Europe, Eastern Europe, Central Asia.

**Pseudapis (Nomiapis) equestris (Gerstäcker, 1872)**

**References.**Ebmer (2014).

**Distribution.** Cyprus, Southern Europe (Greece), Western Asia, Central Asia (Turkestan), Southern Asia (Iran).

**Pseudapis (Nomiapis) valga (Gerstäcker, 1872)**

**References.**Ebmer (2014).

**Distribution.** Cyprus, Southern Europe, (North Macedonia, Spain, Greece), Eastern Europe (Czech Republic), Western Asia, Central Asia (Turkmenistan, Kazakhstan, Tajikistan), Southern Asia (Iran, Pakistan).

**NOMIOIDINI**

**Genus *Ceylalictus* STRAND, 1913**

1 species.

**Ceylalictus (Ceylalictus) variegatus (Olivier, 1789)**

**References.**Cockerell (1931); Mavromoustakis (1949 [“1948”]); Pittioni (1950); Mavromoustakis (1951, 1952); Georghiou (1977); Ebmer (2014).

**Mavromoustakis localities.** Limassol, Cherkes, Zakaki, Akrotiri Bay and Forest, Yermasoyia River, Moni River, Pernera coast of Paralimni, Akrotiri Bay Bogazi shore.

**Material examined.** Paphos District: 2.7 km SW of Acheleia, Potamos tis Ezouzas, 34.729004°N, 32.457544°E, 30.IV.2015, (7♀), collecting pollen on *Tamarix* (Tamaricaceae); Polis, 35.037305°N, 32.391073°E, 30.X.2016, (1♂), visiting *Ceratonia siliqua* (Fabaceae); Larnaca District: Zygi, 34.731233°N, 33.343487°E, 28.X.2016, (1♂), visiting *Ceratonia siliqua* (Fabaceae), all records S.P.M. Roberts leg., A. Pauly det.

**Distribution.** Cyprus, Widespread in Europe, Western Africa, Northern Africa, Western Asia, Central Asia, Southern Asia, Eastern Asia.

**Genus *Nomioides* SCHENCK, 1867**

1 species.

***Nomioides* (*Nomioides) minutissimus* (Rossi, 1790)**

**References.** Mavromoustakis (1949 [“1948”]); Pittioni (1950); Mavromoustakis (1952); Ebmer (2014).

**Mavromoustakis localities.** Limassol, Akrotiri Bay, Yermasoyia River.

**Distribution.** Cyprus, Widespread in Europe, Northern Africa, Western Asia (Turkey, Israel, Georgia), Central Asia, Southern Asia, Eastern Asia (China, Mongolia).

**ROPHITINI**

**Genus *Dufourea* LEPELETIER, 1841**

1 species.

**Dufourea (Cyprirorophites) cypria Mavromoustakis, 1952**

**Type locality–country.** Cyprus, Famagusta: 35.125°N, 33.941667°E, 24.IV.1949, G.A. Mavromoustakis leg., G.A. Mavromoustakis det. ♂, (DAAN).

**References.**Mavromoustakis (1952); Ebmer (2014).

**Mavromoustakis localities.** Famagusta.

**Distribution.** Cyprus, Western Asia (Turkey).

**COLLETIDAE**

**COLLETINI**

**Genus *Colletes* LATREILLE, 1802**

7 species.

***Colletes brevigena* Noskiewicz, 1936**

**References.** Mavromoustakis (1949 [“1948”], 1951, 1952, 1953, 1957a, b).

**Mavromoustakis localities.** Limassol, Polemedia Hills, Akrotiri Forest, Kantara Mountains, Chiflicoudia marshes, Platania Forest, Yermasoyia River, Kellaki, Amathus, Kellaki, Zakaki, Stavrovouni Monastery, Monagroulli, Paramali.

**Material examined.** Nicosia District: Kakopetria, 34.992°N, 32.9082°E, 31.V.2012, 2.VI.2012, (1♀), pan trap (UAEG); Makria Kontarka, 34.9095°N, 32.8971°E, 31.V.2012, 2.VI.2012, (1♀), pan trap (UAEG); Pedoulas, 34°57.711"N, 32°49.733"E, 14.X.2017, (1♂), visiting *Hedera helix*; Between Pedoulas-Kalopanayiwtis, 34°58.308"N, 32°49.775"E, 14.X.2017, (1♂), visiting *Hedera helix*; Limassol District: Troodos, Chionistra, 34.9317°N 32.8664°E, 31.V.2012, 2.VI.2012, (1♀), pan trap (UAEG); Troodos Natural Bath, 34 52.892°N, 32 52.618°E, 27.IV.2016, (1♀), visiting *Cistus creticus*; Yermasoyia Dam, 34.745849°N, 33.083579°E, 28.X.2016, (1♂), visiting *Dittrichia viscosa* (Asteraceae); Yermasoyia Dam, 34.745849°N, 33.083579°E, 28.X.2016, (1♂), visiting *Dittrichia viscosa*; Sovereign Base Area, Paramali, 34.676011°N 32.794947°E, 30.X.2016, (3♂), visiting *Dittrichia viscosa* (Asteraceae); 0.7 km N of Anogyra, 34.743911°N, 32.732439°E, 26.X.2016, (41♂, 3♀), visiting *Ceratonia siliqua* (Fabaceae); Anogyra, 34.729369°N, 32.738368°E, 11.III.2016, (1♂), visiting *Ceratonia siliqua* (Fabaceae); Anogyra, 34.742646°N, 32.730431°E, 11.III.2016, (2♂), visiting *Ceratonia siliqua* (Fabaceae); Anogyra, 34.742646°N, 32.730431°E, 22.X.2016, (1♀), visiting *Ceratonia siliqua* (Fabaceae); Anogyra, 34.729369°N, 32.738368°E, 26.X.2016, (1♂), visiting *Ceratonia siliqua* (Fabaceae); Anogyra, 34.729369°N, 32.738368°E, 11.XII.2016, (1♀), visiting *Ceratonia siliqua* (Fabaceae); Anogyra, 34.729369°N, 32.738368°E, 11.III.2017, , (1♀), visiting *Ceratonia siliqua* (Fabaceae); Akrotiri, 34.600657°N, 32.971419°E, 11.VI.2016, (3♀, 1♂); Paramali, 34.676011°N, 32.794947°E, 30.X.2016, (1♂), visiting *Ceratonia siliqua* (Fabaceae); Akrotiri, 34.600657°N, 32.971419°E, 30.X.2017, (1♀, 1♂), visiting *Dittrichia viscosa*; Sovereign Base Area, Akrotiri, 34.600657 32.971419, 29.X.2016, (3♂, 4♀), visiting *Dittrichia viscosa* (Asteraceae); Larnaca District: Zygi, 34.731233°N, 33.343487°E, 28.X.2016, (5♂, 1♀), visiting *Ceratonia siliqua* (Fabaceae) and *Dittrichia viscosa* (Asteraceae); Zygi, 34.746277°N, 33.384472°E, 28.X.2016, (11♂, 1♀), visiting *Ceratonia siliqua* (Fabaceae); Zygi, 34.731233°N, 33.343487°E, 11.IV.2016, (1♂), visiting *Ceratonia siliqua* (Fabaceae); Zygi, 34.746277°N, 33.384472°E, 11.IV.2016, (1♀), visiting *Ceratonia siliqua* (Fabaceae); Zygi, 34.77458°N, 33.448023°E, 11.IV.2016, (1♀), visiting *Ceratonia siliqua* (Fabaceae); Zygi, 34.731233°N, 33.343487°E, 23.X.2016, (1♀), visiting *Ceratonia siliqua* (Fabaceae); Avdellero, 35 00.598°N 33 35.041°E, 11.IV.2017, (1♂), visiting *Ceratonia siliqua* (Fabaceae); Famagusta District: Achna Dam, 35.05519°N, 33.814011°E, 28.X.2016, (1♂), visiting *Dittrichia viscosa* (Asteraceae); Paphos District: Polis, 35.053539°N, 32.351197°E, 30.X.2016, (10♂), (2♀), visiting *Ceratonia siliqua* (Fabaceae) and *Dittrichia viscosa* (Asteraceae); Polis, 35.036538°N, 32.373117°E, 30.X.2016, (3♂), visiting *Ceratonia siliqua* (Fabaceae); Polis, 35.037305°N, 32.391073°E, 30.X.2016, (3♂, 1♀), visiting *Ceratonia siliqua* (Fabaceae); Polis, 35.032203°N, 32.413732°E, 30.X.2016, det. (5♂, 5♀), visiting *Ceratonia siliqua* (Fabaceae); Mesogi, 34.810747°N, 32.450907°E, 30.X.2016, (7♂, 1♀), visiting *Ceratonia siliqua* (Fabaceae); Polis, 35.053539°N, 32.351197°E, 11.II.2016, (2♂), visiting *Ceratonia siliqua* (Fabaceae) and *Dittrichia viscosa* (Asteraceae); Polis, 35.037305°N, 32.391073°E, 11.II.2016, (1♀); Polis, 35.032203°N, 32.413732°E, 11.II.2016, (1♀); Polis, 35.036538°N, 32.373117°E, 21.X.2016, (1♂); Polis, 35.037305°N, 32.391073°E, 21.X.2016, (1♀); Polis, 35.037305°N, 32.391073°E, 19.XI.2016, (1♀); Polis, 35.037305°N, 32.391073°E, 11.XII.2016, (2♀); Polis, 35.036538°N, 32.373117°E, 11.II.2017, (2♀); Polis, 35.053539°N, 32.351197°E, 11.II.2017, (2♂); Polis, 35.053539°N, 32.351197°E, 11.XII.2017, (2♀); Polis, 35.037305°N, 32.391073°E, 11.XII.2017, (1♀), all Polis records visiting *Ceratonia siliqua* (Fabaceae), all Kakopetria, Makria Kontarka and Troodos Chionistra records S. Dimitriou leg., J. Devalez det., all Pedoulas, Between Pedoulas-Kalopanayiotis, Troodos Natural Bath, Yermasoyia Dam, Anogyra, Paramali, Akrotiri, Zygi and Polis records A. Varnava leg., M. Kuhlmann det., all Yermasoyia Dam, Sovereign Base Area, Paramali, 0.7 km N of Anogyra, Sovereign Base Area, Akrotiri, Zygi, Achna Dam, Polis, Mesogi records S.P.M. Roberts leg., M. Kuhlmann det.

**Distribution.** Cyprus, Western Europe (France, Austria), Southern Europe, Eastern Europe (Hungary, Bulgaria, Ukraine), Western Asia (Turkey).

***Colletes creticus* Noskiewicz, 1936**

**Material examined.** Limassol District: Sovereign Base Area, Episkopi, Kensington Cliffs, 34.670772°N, 32.846923°E, 4.V.2015, (1♂); Pissouri 2 km S, 34.654385°N, 32.717924°E, 30.X.2016, (3♂), visiting *Dittrichia viscosa* (Asteraceae) and *Ceratonia siliqua* (Fabaceae), all records S.P.M. Roberts leg., M. Kuhlmann det.

**Distribution.** Cyprus, Southern Europe (Greece).

***Colletes cyprius* Noskiewicz, 1936**

**Type locality–country.** Cyprus, Kykkos mountains, 1500m: 34.984°N, 32.741°E, V-VI.1922/1923 or 1930, G.A. Mavromoustakis leg., J. Noskiewicz det.

**References.**Noskiewicz (1936); Mavromoustakis (1949 [“1948”], 1951, 1952, 1957a).

**Mavromoustakis localities.** Limassol, Mesayitonia, Cherkes, Yermasoyia Hills, Asomatos, Alassa River, Pera Pedi, Akrotiri Bay, Stroumbi, Livadin of Cedar (Paphos Forest), Potamitissa, Akrounda, Moni, Kellaki, Larnaca, Kykkou Monastery, Ayia Phyla, Mt. Troodos, Near Mesayitonia, Bogazi, Kathikas, Eftagonia, Xerokolimbi near Trooditissa.

**Material examined.** Paphos District: 15 km SE Paphos, Kouklia, 34.72°N, 32.55°E, 20.VI.2013, (1♂); 8 km N Paphos, Mavrokolympos Reservoir, 34.85°N, 32.40°E, 20.VI.2013, (1♂); Polis, 35.053539°N, 32.351197°E, 30.X.2016, (1♂), visiting *Ceratonia siliqua* (Fabaceae); Polis, 35.036538°N, 32.373117°E, 11.II.2017, (1♂), visiting *Ceratonia siliqua* (Fabaceae); Limassol District: Anogyra, 34.729369°N, 32.738368°E, 11.V.2015, (1♀), visiting *Ceratonia siliqua* (Fabaceae); Akrotiri, 34.600657°N, 32.971419°E, 5.VIII.2016, (1♀); Anogyra, 34.729369°N, 32.738368°E, 26.X.2016 (1♀), visiting *Ceratonia siliqua* (Fabaceae); Anogyra, 34.729369°N, 32.738368°E, 11.III.2017, (1♂), visiting *Ceratonia siliqua* (Fabaceae); Anogyra, 34.729369°N, 32.738368°E, 18.X.2017, (2♀), visiting *Ceratonia siliqua* (Fabaceae), all 15 km SE Paphos, Kouklia and Mavrokolympos Reservoir records, C. Schmid-Egger leg., M. Kuhlmann det., all Polis and Anogyra records A. Varnava leg., M. Kuhlmann det., all Anogyra (11.V.2015) records S. Louca leg., M. Kuhlmann det.

**Distribution. Cyprus. ENDEMIC.**

***Colletes hylaeiformis* Eversmann, 1852**

**References.**Scheuchl and Willner (2016).

**Distribution.** Cyprus, Western Europe (France, Germany, Austria), Southern Europe, Eastern Europe, Western Asia (Turkey, Georgia, Azerbaijan), Central Asia.

***Colletes maidli* Noskiewicz, 193**﻿**6**

**References.** Mavromoustakis (1949 [“1948”], 1952); Georghiou (1977).

**Mavromoustakis localities.** Asomatos, Yermasoyia River, Evdhimou River, Famagusta.

**Material examined.** Limassol District: Akrotiri, 34.583676°N, 32.949306°E, 20.VI.2016, (1♀); Akrotiri, 34.352103°N, 32.562132°E, 20.VI.2016, (1♂), all records A. Varnava leg., M. Kuhlmann det.

**Distribution.** Cyprus, Western Europe (France), Southern Europe, Eastern Europe, Western Asia, Central Asia.

***Colletes similis* Schenck, 1853**

**References.** Mavromoustakis (1949 [“1948”], 1951, 1952, 1953).

**Mavromoustakis localities.** Limassol, Akrotiri Bay, Asomatos, Moni, Yerasa, Kato Platres, Pera Pedi, Monagroulli, Platania Forest Station, Mt. Troodos.

**Material examined.** Limassol District: Sovereign Base Area, Akrotiri, 34.600657°N, 32.971419°E, 29.X.2016, (1♀), visiting *Dittrichia viscosa* (Asteraceae); Paphos District: Polis, 35.053539°N, 32.351197°E, 30.X.2016, (5♀), visiting *Dittrichia viscosa* (Asteraceae), all records S.P.M. Roberts leg., M. Kuhlmann det.

**Distribution.** Cyprus, Widespread in Europe, Northern Africa, Western Asia, Central Asia, Southern Asia (Iran), Eastern Asia (China).

***Colletes squamulosus* Noskiewicz, 1936**

**References.** Mavromoustakis (1949 [“1948”]).

**Mavromoustakis localities.** Larnaca.

**Distribution.** Cyprus, Western Asia, Central Asia (Turkmenistan).

**HYLAEINI**

**Genus *Hylaeus* FABRICIUS, 1793**

**12 species.**

**Hylaeus (Paraprosopis) clypearis (Schenck, 1853)**

**References.**Erlandsson (1986).

**Distribution.** Cyprus, Western Europe, Southern Europe, Eastern Europe, Northern Africa, Western Asia (Turkey, Georgia).

**Hylaeus (Abrupta) cornutus Curtis, 1831**

**References.** Mavromoustakis (1949 [“1948”]).

**Mavromoustakis localities.** Limassol, Zakaki, Asomatos, Pera Pedi, Larnaca.

**Distribution.** Cyprus, Widespread in Europe, Northern Africa, Western Asia (Israel), Central Asia (Turkmenistan), Southern Asia (Iran).

**Hylaeus (Spatulariella) cypricola (Warncke, 1972)**

**References.**Pittioni (1950); Mavromoustakis (1957a).

**Mavromoustakis localities.** Cherkes.

**Material examined.** Limassol District: Anogyra, 34.741952°N, 32.734845°E, 3.V.2015, (2♂), visiting *Sinapis* (Brassicaceae); Sovereign Base Area, Episkopi Kensington Cliffs, 34.670772°N, 32.846923°E, 4.V.2015, (5♂, 3♀); Larnaca District: Zygi, 34.731233°N, 33.343487°E, 28.X.2016, (1♀), visiting *Ceratonia siliqua* (Fabaceae), all records S.P.M. Roberts leg., G. Holmström and H. Dathe det.

**Distribution.** Cyprus, Western Asia (Turkey), Northern Africa (Egypt).

**Notes.** Described from Cyprus as Spatulariella (Brachyspatulariella) dimidiatus Pittioni, 1950, from Limassol: 34.66839°N, 33.03252°E, G.A. Mavromoustakis leg., K. Warncke det., (MZHF).

**Hylaeus (Prosopis) gibbus Saunders, 1850**

**References.**Alfken (1912).

**Material examined.** Limassol District: Sovereign Base Area, Akrotiri, 34.628771°N, 32.941031°E, 29.X.2016, (1♂, 2♀), visiting *Dittrichia viscosa* (Asteraceae); Paphos District: 2.7 km SW of Acheleia, Potamos tis Ezouzas, 34.729004°N, 32.457544°E, 30.IV.2015, (1♂, 2♀), all records S.P.M. Roberts leg., G. Holmström and H. Dathe det.

**Distribution.** Cyprus, Widespread in Europe, Northern Africa (Morocco, Egypt), Western Asia Central Asia (Kazakhstan), Southern Asia (Iran).

**Notes.** Although the Cyprus specimens listed below were determined by H. Dathe, he himself believes that DNA analysis could reveal that they might be specimens of *H. incongruus* Forster, 1871, *H. stigmorhinus* (Pérez, 1895) or the dark form of *H. pictus* (Smith, 1853) (with black scutellum) (Dathe, 2018 in litt.). It is clear that a revision of this group is required to elucidate the nature of this species group.

**Hylaeus (Dentigera) imparilis Förster, 1871**

**References.**Alfken (1928); Pittioni (1950).

**Material examined.** Limassol District: 1 km E of Pissouri, 34.677579°N, 32.722066°E, 27.IV.2015, (2♂, 1♀), visiting Brassicaceae; Anogyra to Pachna Road km 4, 34.764269°N, 32.757736°E, 5.V.2015, (1♂); 0.7 km N of Anogyra, 34.743911°N, 32.732439°E, 26.X.2016, (1♀), visiting *Ceratonia siliqua* (Fabaceae); 1 km S of Anogyra, 34.727647°N, 32.73462°E, 26.X.2016, (1♀), visiting *Ceratonia siliqua* (Fabaceae); Paphos District: Nr Arminou Reservoir, 34.883435°N, 32.750988°E, 29.IV.2015, (1♂); Asprokremmos Dam, 34.720825°N, 32.551994°E, 30.IV.2015, (1♂, 2♀), visiting *Sisymbrium* (Brassicaceae); 2.7 km SW of Acheleia, Potamos tis Ezousas 34.729004°N, 32.457544°E, 30.IV.2015, (1♂); Larnaca District: Zygi, 34.731233°N, 33.343487°E, 28.X.2016, (6♀), visiting *Dittrichia viscosa* (Asteraceae) and *Ceratonia siliqua* (Fabaceae), all records S.P.M. Roberts leg., G. Holmström and H. Dathe det.

**Distribution.** Cyprus, Western Europe (France, Switzerland, Austria), Southern Europe, Eastern Europe, Northern Africa (Morocco, Tunisia), Western Asia, Southern Asia (Iran).

**Notes.** Described from Cyprus as Prosopis brevicornis var. cypria Alfken, 1928, from [Yermasoyia; Episcopi]: 34.72231°N, 33.08497°E, 34.67513°N, 32.88260°E, G.A. Mavromoustakis leg., J.D. Alfken det.

**Hylaeus (Paraprosopis) lineolatus (Schenck, 1861)**

**References.** Mavromoustakis (1949 [“1948”]); Pittioni (1950); Mavromoustakis (1957a).

**Mavromoustakis localities.** Finikaria River, Mesapotamos, Near Zakaki, Near Mesayitonia.

**Distribution.** Cyprus, Western Europe, Southern Europe, Eastern Europe, Northern Africa (Morocco, Algeria), Western Asia (Israel, Lebanon, Azerbaijan), Southern Asia (Iran).

**Notes.** Described from Cyprus as *Prosopis lineolata rudis* Pittioni, 1950, from Kykkos: 34.984°N, 32.741°E, 15-17.VII.1939, H. Lindberg leg., B. Pittioni det., (MZHF).

**Hylaeus (Prosopis) meridionalis Förster, 1871**

**References.**Alfken (1928); Mavromoustakis (1949 [“1948”], 1951, 1957a).

**Mavromoustakis localities.** Akrotiri Forest, Cherkes, Zakaki, Asomatos, Fassouri, Gape Gata, Larnaca, Pernera coast of Paralimni.

**Distribution.** Cyprus, Western Europe (France, Switzerland, Austria), Southern Europe, Eastern Europe, Northern Africa (Morocco), Western Asia, Central Asia (Kyrgyzstan), Southern Asia (Iran).

**Notes.** Described from Cyprus as *Prosopis maculiscutum* Alfken, 1928, from Cherkes: 34.65°N, 32.975°E, 7 or 24.VIII.1924, G.A. Mavromoustakis leg., J.D. Alfken det.

**Hylaeus (Prosopis) pictus (Smith, 1853)**

**References.**Mavromoustakis (1951, 1952).

**Mavromoustakis localities.** Near Limassol, Cherkes, Chiflicoudia marshes, Pera Pedi, Near Akrotiri, Yermasoyia River, Potamitissa, Mt. Troodos Kannoures springs, Near Cape Gata.

**Distribution.** Cyprus, Western Europe (France), Southern Europe, Eastern Europe (Hungary, Bulgaria), Northern Africa, Western Asia.

**Hylaeus (Dentigera) rubicola Saunders, 1850**

**References.**Alfken (1928); Mavromoustakis (1949 [“1948”]); Pittioni (1950).

**Mavromoustakis localities.** Yermasoyia.

**Distribution.** Cyprus, Western Europe (France), Southern Europe (Spain, Italy, Greece), Eastern Europe (Bulgaria), Northern Africa (Egypt), Western Asia.

**Notes.** Described from Cyprus as *Prosopis rectanguliceps* Alfken, 1928, from Yermasoyia: 34.7182°N, 33.08788°E, 1-20.IX.1924, G.A. Mavromoustakis leg., J.D. Alfken det.

**Hylaeus (Lambdopsis) scutellatus (Spinola, 1838)**

**References.**Alfken (1928); Mavromoustakis (1949 [“1948”]); Pittioni (1950); Mavromoustakis (1951, 1957a).

**Mavromoustakis localities.** Limassol, Cherkes, Zakaki, Moni, Pera Pedi, Stroumbi, Tsada.

**Material examined.** Paphos District: Asprokremmos Dam, 34.720825°N, 32.551994°E, 30.IV.2015, S.P.M. Roberts leg., G. Holmström and H. Dathe det. (1♂), visiting *Sisymbrium* (Brassicaceae).

**Distribution.** Cyprus, Southern Europe, Northern Africa (Egypt), Western Asia (Palestine to Turkey), Southern Asia (Iran).

**Hylaeus (Paraprosopis) soror (Pérez, 1903)**

**References.**Pittioni (1950).

**Distribution.** Cyprus, Western Europe (France), Southern Europe, Eastern Europe (Bulgaria, Russian Federation), Northern Africa, Southern Asia (Iran).

**Hylaeus (Paraprosopis) taeniolatus Förster, 1871**

**References.**Erlandsson (1986).

**Material examined.** Limassol District: Anogyra, 34.73663°N, 32.732715°E, 26.IV.2015, (1♂), visiting Brassicaceae; 1 km E of Pissouri, 34.677579°N, 32.722066°E, 27.IV.2015, (4♀, 1♂), visiting Brassicaceae; 2 km N of Anogyra, 34.748126°N, 32.732248°E, 1.V.2015, (1♀); Anogyra to Avdimou Road km 2, 34.723986°N, 32.736892°E, 3.V.2015, (1♂), visiting Apiaceae; Anogyra, 34.741952°N, 32.734845°E, 3.V.2015, (1♀, 3♂), visiting Brassicaceae; 0.7 km N of Anogyra, 34.745537°N, 32.73385°E, 3.V.2015, (8♂); 0.7 km N of Anogyra, 34.743911°N, 32.732439°E, 26.X.2016, (1♀), visiting Fabaceae; Yermasoyia Dam, 34.755799°N, 33.096194°E, 7.III.2017, (1♂); Paphos District: Nr Arminou Reservoir, 34.883435°N, 32.750988°E, 29.IV.2015, (1♀, 1♂); Asprokremmos Dam, 34.720825°N, 32.551994°E, 30.IV.2015, (1♀, 1♂), visiting Brassicaceae; 2.7 km SW of Acheleia, Potamos tis Ezouzas, 34.729004°N, 32.457544°E, 30.IV.2015, (2♂); Polis, 35.053539°N, 32.351197°E, 30.X.2016, (1♀, 1♂), visiting Fabaceae; Larnaca District: Zygi, 34.731233°N, 33.343487°E, 28.X.2016, (1♀, 4♂), visiting Asteraceae and Fabaceae, all records S.P.M. Roberts leg., G. Holmström and H. Dathe det.

**Distribution.** Cyprus, Western Europe, Southern Europe, Eastern Europe (Bulgaria), Northern Africa, Western Asia.

**MEGACHILIDAE**

**LITHURGINI**

**Genus *Lithurgus* BERTHOLD, 1827**

2 species.

**Lithurgus (Lithurgus) chrysurus Fonscolombe, 1834**

**References.** Mavromoustakis (1949 [“1948”], 1952, 1954, 1957a).

**Mavromoustakis localities.** Near Limassol, Cherkes, Pera Pedi, Mandria, Near Kalliana.

**Distribution.** Cyprus, Western Europe (France, Switzerland, Austria), Southern Europe, Eastern Europe, Northern Africa (Morocco), Western Asia, Central Asia (Tajikistan), Southern Asia (Iran), Eastern Asia (China).

**Lithurgus (Lithurgus) tibialis Morawitz, 1875**

**References.** Mavromoustakis (1949 [“1948”], 1957a).

**Mavromoustakis localities.** Cherkes, Zakaki, Asomatos, Episkopi, Pera Pedi, Fassouri, Near Famagusta, Symboulas.

**Material examined.** Paphos District: 15 km SE Paphos, Kouklia, 34.72°N, 32.55°E, 20.VI.2013, (1♂); 20 km N Paphos, Kathikas, 34.90°N, 32.42°E, 20.VI.2013, (2♂), all records C. Schmid-Egger leg., C. Schmid-Egger det.

**Distribution.** Cyprus, Southern Europe, Northern Africa, Western Asia, Central Asia (Turkmenistan), Southern Asia (Iran, Pakistan).

**OSMIINI**

**Genus *Chelostoma* LATREILLE, 1809**

3 species.

**Chelostoma (Chelostoma) comosum Müller, 2012**

**Type locality–country.** Cyprus, Troodos, Kato Platres S of Foini: 34.8775°N, 32.8386°E, 1.V.2011, C. Sedivy, A. Müller leg., A. Müller det. ♂, (ETHZ).

**References.**Müller (2012).

**Material examined.** Larnaca District: 2 km S of Ora, 450m, 34.85°N, 33.20°E, 3.IV.2009, H.R. Schwenninger leg., A. Müller det. (1♀); Limassol District: Anogyra, 34.73663°N, 32.732715°E, 26.IV.2015, M. Jenner leg., G. Le Goff det. (1♂); Famagusta District: 5 km E of Rizokarpaso, 35.62°N, 34.46°E, C. Schmid-Egger leg., A. Müller det. (1♂).

**Distribution.** Cyprus, Western Asia.

**Chelostoma (Chelostoma) diodon Schletterer, 1889**

**References.**Mavromoustakis (1951, 1952, 1957a).

**Mavromoustakis localities.** Yerasa, Platania Forest Station.

**Material examined.** Limassol District: Anogyra to Pachna Road km 4, 34.764269°N, 32.757736°E, 5.V.2015, S.P.M. Roberts leg., A. Müller det. (1♀), visiting Asteraceae.

**Distribution.** Cyprus, Southern Europe (Greece), Western Asia, Southern Asia (Iran).

**Notes.** The subspecies described from Cyprus is *Chelostoma diodon cypriacum* (Mavromoustakis, 1951), from Yerasa: 34.8025°N, 32.998056°E, 22.III.1945, G.A. Mavromoustakis leg., G.A. Mavromoustakis det. ♀, (DAAN).

**Chelostoma (Chelostoma) lucens (Benoist, 1928)**

**References.**Mavromoustakis (1951, 1952, 1957a).

**Mavromoustakis localities.** Apsiou, Yerasa.

**Distribution.** Cyprus, Southern Europe (Greece), Eastern Europe (Bulgaria), Western Asia.

**Genus *Heriades* SPINOLA, 1808**

4 species.

**Heriades (Rhopaloheriades) clavicornis Morawitz, 1875**

**References.** Ungricht, Müller and Dorn (2008).

**Distribution.** Cyprus, Southern Europe (Greece), Western Asia, Central Asia (Tajikistan), Southern Asia (Iran).

**Heriades (Michenerella) punctulifera Schletterer, 1889**

**References.**Mavromoustakis (1951).

**Mavromoustakis localities.** Mt. Troodos.

**Distribution.** Cyprus, Southern Europe, Eastern Europe (Bulgaria), Western Asia.

**Notes.** The subspecies described from Cyprus is *Heriades punctulifera troodica* (Mavromoustakis, 1951), from Mt. Troodos, open slopes, 1676.4m: 34.9234°N, 32.8833°E, 18.VII.1949 [“1948”], G.A. Mavromoustakis leg. G.A. Mavromoustakis det. ♀, (DAAN).

**Heriades (Heriades) rubicola Pérez, 1890**

**References.** Mavromoustakis (1949 [“1948”], 1952).

**Mavromoustakis localities.** Near Limassol, Akrotiri Bay, Cherkes, Chiflicoudia marshes, Erimi, Alassa River, Evdhimou River.

**Material examined.** Paphos District: 20 km NNW Paphos, Lara Beach, 34.94°N, 32.31°E, 20.VI.2013, (4♀); Polis, 35.053539°N, 32.351197°E, 30.X.2016, (2♂), visiting *Dittrichia viscosa* (Asteraceae); Polis, 35.053539°N, 32.351197°E, 30.X.2016, (6♂), visiting *Dittrichia viscosa*; Limassol District: Sovereign Base Area, Akrotiri, 34.628771°N, 32.941031°E, 29.X.2016, (1♀), visiting *Dittrichia viscosa* (Asteraceae); Cherkes, 34.653067°N, 32.974233°E, 22.V.2016, (1♂); Cherkes, 34.65096°N, 32.99091°E, 26.VIII.2016, (1♀); Cherkes, 34.65096°N, 32.99091°E, 12.VIII.2016, (1♀); Cherkes, 34.6432°N 32.9952°E, 6.XI.2016, (1♀); Akrotiri, 34.628817°N, 32.940667°E, 3.VII.2016, (2♂); Akrotiri, 34.628817°N, 32.940667°E, 26.VIII.2016, (1♀); Akrotiri, 34.600657°N, 32.971419°E, 11.IX.2016, (1♂); Akrotiri, 34.628817°N, 32.940667°E, 8.X.2016, (1♀); Akrotiri, 34.628817°N, 32.940667°E, 6.XI.2016, (1♀); Akrotiri, 34.628817°N, 32.940667°E, 21.IV.2017, (1♀); Akrotiri, 34.628817°N, 32.940667°E, 3.VII.2017, (1♀, 1♂), all 20 km NNW Paphos and Lara Beach (20.VI.2013) records, C. Schmid-Egger leg., A. Müller det., all Polis (30.X.2016, 2♂) and Sovereign Base Area, Akrotiri records S.P.M. Roberts leg., S.P.M. Roberts and G. Le Goff det., all Polis (30.X.2016, 6♂), all Cherkes and Akrotiri records A. Varnava leg., A. Müller det.

**Distribution.** Cyprus, Western Europe (France, Switzerland), Southern Europe, Eastern Europe (Bulgaria, Slovakia), Northern Africa, Western Asia, Central Asia (Turkmenistan, Kazakhstan, Kyrgyzstan), Southern Asia.

**Heriades (Heriades) truncorum (Linnaeus, 1758)**

**References.** Mavromoustakis (1949 [“1948”]); Pittioni (1950); Mavromoustakis (1952).

**Mavromoustakis localities.** Near Limassol, Chiflicoudia marshes, Erimi, Near Platania Forest Station, Rizokarpaso.

**Material examined.** Paphos District: 20 km NNW Paphos, Lara Beach, 34.94°N, 32.31°E, 20.VI.2013, (1♀); Polis, 35.053539°N, 32.351197°E, 30.X.2016, (1♀), visiting *Dittrichia viscosa* (Asteraceae); Limassol District: Cherkes, 34.653067°N, 32.974233°E, 22.V.2016, (2♀, 1♂); Cherkes, 34.659801°N, 32.99051°E, 22.V.2016, (1♀); Cherkes, 34.659801°N, 32.99051°E, 5.VI.2016, (2♀); Akrotiri, 34.600657°N, 32.971419°E, 22.V.2016, (1♂); Akrotiri, 34.628817°N, 32.940667°E, 13.X.2017, (1♀); Platres, 34.886528°N, 32.862465°E, 14.X.2017, (1♀), visiting *Hedera helix*, all 20 km NNW Paphos, Lara Beach records C. Schmid-Egger leg., A. Müller det., all Polis records S.P.M. Roberts leg., S.P.M. Roberts and G. Le Goff det., all Limassol district records A. Varnava leg., A. Müller det.

**Distribution.** Cyprus, Widespread in Europe, Northern Africa, Western Asia.

**Genus *Hoplitis* KLUG, 1807**

9 species.

**Hoplitis (Alcidamea) acuticornis (Dufour & Perris, 1840)**

**References.**Mavromoustakis (1939b, 1949 [“1948”]).

**Mavromoustakis localities.** Sphalangiotissa Monastery.

**Material examined.** Limassol District: Sovereign Base Area, Akrotiri, Bishop's Pool, 34.597305°N, 32.984521°E, 28.IV.2015, M. Jenner leg., G. Le Goff det. (1♀); Polemidia, 34.71178°N, 33.004775°E, 8.III.2017, Bee Course students leg., A. Müller det. (2♂, 1♀).

**Distribution.** Cyprus, Widespread in Europe (except Great Britain and northern Europe), Northern Africa, Western Asia, Central Asia, Southern Asia (Iran).

**Hoplitis (Anthocopa) anipuncta (Alfken, 1935)**

**References.**Müller (2018).

**Distribution.** Cyprus, Western Asia.

**Hoplitis (Hoplitis) annulata (Latreille, 1811)**

**References.**Mavromoustakis (1939b, 1949 [“1948”], 1951).

**Material examined.** Paphos District: 20 km NNW Paphos, Lara Beach, 34.94°N, 32.31°E, 20.VI.2013, (2♀); 8 km N Paphos, Mavrokolympos Reservoir, 34.85°N, 32.40°E, 20.VI.2013, (1♀); Asprokremmos Dam, 34.720825°N, 32.551994°E, 30.IV.2015, (1♂, 1♀), visiting *Echium angustifolium* (Boraginaceae); Limassol District: Sovereign Base Area, 8 km S Limassol, Akrotiri (near Airbase), 34.60°N, 32.97°E, 20.VI.2013, (2♀); Sovereign Base Area, Akrotiri, Bishop's Pool, 34.597305°N, 32.984521°E, 28.IV.2015, (1♂, 4♀), visiting *Echium angustifolium* (Boraginaceae); Cherkes, 34.65096°N, 32.99091°E, 9.IV.2016, (1♀); Akrotiri, 34.600657°N, 32.971419°E, 19.IV.2016, (1♂); Akrotiri, 34.600657°N, 32.971419°E, 22.V.2016, (2♂); Akrotiri, 34.601506°N, 32.986197°E, 22.V.2016, (2♂); Akrotiri, 34.600657°N, 32.971419°E, 5.V.2017, (1♀, 1♂); Akrotiri, 34.600657°N, 32.971419°E, 24.V.2017, (1♀); Akrotiri, 34.600657°N, 32.971419°E, 15.VI.2017, (1♀); Akrotiri, 34.600657°N, 32.971419°E, 26.IV.2018, (1♀); Akrotiri, 34.601506°N, 32.986197°E, 26.IV.2018, (1♂) Yermasoyia Dam, 34.755799°N, 33.096194°E, 7.III.2017, (1♂), all 20 km NNW Paphos, Lara Beach, Mavrokolympos Reservoir and Sovereign Base Area, 8 km S Limassol, Akrotiri (near Airbase) records C. Schmid-Egger leg., A. Müller det., all Asprokremmos Dam and Sovereign Base Area, Akrotiri, Bishop's Pool records S.P.M. Roberts leg., A. Müller det., all Yermasoyia Dam records S.P.M. Roberts leg., G. Le Goff det., all Cherkes and Akrotiri records A. Varnava leg., A. Müller det.

**Distribution.** Cyprus, Western Europe (France), Southern Europe, Eastern Europe (Bulgaria), Northern Africa, Western Asia.

**Hoplitis (Anthocopa) cypriaca (Mavromoustakis, 1938)**

**Type locality–country.** Cyprus, Limassol: 34.66839°N, 33.03252°E, 21.IV.1938, G.A. Mavromoustakis leg., G.A. Mavromoustakis det. ♀, (DAAN).

**References.**Mavromoustakis (1938, 1949 [“1948”], 1952, 1953, 1954).

**Mavromoustakis localities.** Limassol, Evdhimou River, Near Enkomi of Famagusta, Xylophagou, Arakapas, Deryneia.

**Material examined.** Limassol District: Akrotiri, 34.583676°N, 32.949306°E, 27.IV.2018, A. Varnava leg, A. Müller det. (1♀).

**Distribution.** Cyprus, Western Asia (Israel, Jordan, Syria).

**Hoplitis (Anthocopa) fasciculata (Alfken, 1934)**

**References.** Mavromoustakis (1949 [“1948”]); Pittioni (1950); Mavromoustakis (1952).

**Mavromoustakis localities.** Cherkes, Yermasoyia River, Younaros of Zakaki, Deryneia.

**Material examined.** Paphos District: 15 km SE Paphos, Kouklia, 34.72°N, 32.55°E, 20.VI.2013, (4♀); Limassol District: Akrotiri, 34.583676°N, 32.949306°E, 24.V.2017, (1♂); Akrotiri, 34.583676°N, 32.949306°E, 26.IV.2018, (1♀), all Paphos district records C. Schmid-Egger leg., A. Müller det., all Limassol district records A. Varnava leg., A. Müller det.

**Distribution.** Cyprus, Southern Europe (Italy, Greece), Western Asia, Southern Asia (Iran).

**Notes.** Described from Cyprus as *Osmia idalia* Mavromoustakis, 1949 [“1948"], from Yermasoyia River: 34.7182°N, 33.08788°E, 5.VI.1938, G.A. Mavromoustakis leg., G.A. Mavromoustakis det. ♀, (DAAN).

**Hoplitis (Hoplitis) holmboei (Mavromoustakis, 1949)**

**Type locality–country.** Cyprus, Yermasoyia River: 34.7182°N, 33.08788°E, 20.III.1937, G.A. Mavromoustakis leg., G.A. Mavromoustakis det. ♀, (DAAN).

**References.** Mavromoustakis (1949 [“1948”], 1952, 1957a).

**Mavromoustakis localities.** Polemedia Hills, Yermasoyia River, Near Amathus.

**Material examined.** Limassol District: Almirolivado, 34.9333°N, 32.9004°E, 14-16.V.2012, S. Dimitriou leg., A. Müller det. (1♂), pan trap (UAEG).

**Distribution.** Cyprus, Southern Europe (Greece).

**Hoplitis (Alcidamea) limassolica (Mavromoustakis, 1937)**

**Type locality–country.** Cyprus, Limassol: 34.66839°N, 33.03252°E, 18.III.1935, G.A. Mavromoustakis leg., G.A. Mavromoustakis det. ♀, (DAAN).

**References.**Mavromoustakis (1937b, 1938, 1949 [“1948”], 1951, 1952, 1953, 1957a).

**Mavromoustakis localities.** Polemedia Hills, Yerasa, Kellaki, Trimiklini, Famagusta, Ayia Varvara (Stavrovouni).

**Material examined.** Limassol District: Polemidia, 34.71178°N, 33.004775°E, 8.III.2017, Bee Course students leg., A. Müller det. (1♂).

**Distribution.** Cyprus, Northern Africa, Western Asia.

**Hoplitis (Pentadentosmia) pomarina (Warncke, 1991)**

**References.**Müller (2018).

**Material examined.** C. Schmid-Egger leg., A. Müller det., 2013.

**Distribution.** Cyprus, Southern Europe (North Macedonia, Greece), Western Asia.

**Hoplitis (Anthocopa) yermasoyiae (Mavromoustakis, 1938)**

**References.**Mavromoustakis (1938, 1949 [“1948”], 1951, 1952).

**Mavromoustakis localities.** Polemedia Hills, Potamitissa, Yerasa, Kellaki, Near Eftagonia, Ayia Irini Station, Eleousa Monastery (Karpasian Peninsula).

**Material examined.** Nicosia District: Kakopetria, 34.992°N, 32.9082°E, 5-7.IV.2012, (6♂, 2♀), pan trap (UAEG); Kakopetria, 34.992°N, 32.9082°E, 25-27.IV.2012, (14♂, 10♀), pan trap (UAEG); Limassol District: 1 km E of Pissouri, 34.677579°N, 32.722066°E, 27.IV.2015, (1♀); 0.7 km N of Anogyra, 34.745537°N, 32.73385°E, 3.V.2015, (1♀); Anogyra to Pachna Road km 4, 34.764269°N, 32.757736°E, 5.V.2015, (2♀), visiting Asteraceae; Akrotiri, 34.583676°N, 32.949306°E, 23.IV.2016, (1♀); Akrotiri, 34.600657°N, 32.971419°E, 23.IV.2016, (1♀); Yermasoyia Dam, 34.755799°N, 33.096194°E, 7.III.2017, (1♂); Akrotiri, 34.601506°N, 32.986197°E, 7.IV.2017, (1♀); Akrotiri, 34.601506°N, 32.986197°E, 21.IV.2017, (1♂); Akrotiri, 34.601506°N, 32.986197°E, 14.IV.2018, (1♀, 1♂); Akrotiri, 34.601506°N, 32.986197°E, 26.IV.2018, (2♀); Paphos District: N of Elia Bridge, 34.900977°N, 32.776759°E, 29.IV.2015, (1♂), all Nicosia district records S. Dimitriou leg., A. Müller det., all 1 km E of Pissouri, 0.7 km N of Anogyra, Anogyra to Pachna Road km 4 and Paphos district records S.P.M. Roberts leg., A. Müller det., all Akrotiri records A. Varnava leg., A. Müller det., all Yermasoyia Dam records Bee Course students leg., A. Müller det.

**Distribution.** Cyprus, Southern Europe (North Macedonia, Albania, Greece), Western Asia.

**Notes.** The subspecies described from Cyprus is *Hoplitis yermasoyiae yermasoyiae*, from Yermasoyia: 34.7182°N, 33.08788°E, 21.III.1937, G.A. Mavromoustakis leg./det. ♂, (DAAN).

**Genus *Osmia* PANZER, 1806**

25 species.

**Osmia (Pyrosmia) amathusica Mavromoustakis, 1937**

**Type locality–country.** Cyprus, Amathus ruins: 34.7125°N, 33.1419°E, 26.III.1935, G.A. Mavromoustakis leg., G.A. Mavromoustakis det. ♀, (DAAN).

**References.**Mavromoustakis (1937b, 1949 [“1948”], 1957a).

**Mavromoustakis localities.** Polemedia Hills, Choirokitia.

**Material examined.** Limassol District: Polemidia, 34.71178°N, 33.004775°E, 8.III.2017, Bee Course students leg., A. Müller det. (1♂).

**Distribution.** Cyprus, Southern Europe (Italy, Greece), Western Asia.

**Osmia (Erythrosmia) andrenoides Spinola, 1808**

**References.**Mavromoustakis (1939b, 1949 [“1948”], 1951).

**Mavromoustakis localities.** Limassol, Polemedia Hills.

**Distribution.** Cyprus, Western Europe, Southern Europe, Eastern Europe, Western Asia.

**Osmia (Osmia) bicornis (Linnaeus, 1758)**

**References.**Mavromoustakis (1939b, 1949 [“1948”], 1951, 1952, 1953).

**Mavromoustakis localities.** Apsiou, Yerasa, Trimiklini, Pera Pedi, Mt. Troodos open slopes, Listovounos Kakopetria.

**Material examined.** Nicosia District: Kakopetria, 34.992°N, 32.9082°E, 25-27.IV.2012, S. Dimitriou leg., J. Devalez and A. Müller det. (1♂), pan trap (UAEG).

**Distribution.** Cyprus, Widespread in Europe, Northern Africa, Western Asia, Central Asia (Turkmenistan, Kazakhstan, Kyrgyzstan), Southern Asia (Iran).

**Osmia (Metallinella) brevicornis (Fabricius, 1798)**

**References.**Scheuchl and Willner (2016).

**Distribution.** Cyprus, Widespread in Europe, Northern Africa, Western Asia, Central Asia, Southern Asia (Iran, Afghanistan).

**Notes.** Records pertain to ssp. leucogastra Morawitz, 1875.

**Osmia (Helicosmia) caerulescens (Linnaeus, 1758)**

**References.**Mavromoustakis (1939b, 1949 [“1948”]); Pittioni (1950); Mavromoustakis (1951, 1952, 1953, 1957a).

**Mavromoustakis localities.** Episkopi Forest, Potamitissa, Trimiklini, Platres, Ayios Ioannis tou Agrou, Asbestotos spring near Amathus, Platania Forest Station, Mt. Troodos open slopes, Livadin of Cedars (Paphos Forest).

**Material examined.** Limassol District: Amiantos, 34.918°N, 32.9472°E, 25-27.IV.2012, (1♂), pan trap (UAEG); Troodos, Chionistra, 34.9317°N, 32.8664°E, 14-16.V.2012, (1♂), pan trap (UAEG); Yermasoyia Dam, 34.755799°N, 33.096194°E, 7.III.2017, (1♂); Sovereign Base Area, Akrotiri, Bishop's Pool, 34.597305°N, 32.984521°E, 10.III.2017, (2♂); Sovereign Base Area, Episkopi, Kensington Cliffs, 34.670772°N, 32.846923°E, 4.V.2015, (1♀); Paphos District: 8 km N Paphos, Mavrokolympos Reservoir, 34.85°N, 32.40°E, 20.VI.2013, (1♀); Famagusta District: Cape Greco, 34.963264°N, 34.066211°E, 15.III.2017, (1♂), all Amiantos records S. Dimitriou leg., J. Devalez and A. Müller det., all Troodos, Chionistra records S. Dimitriou leg., A. Müller det., all Yermasoyia Dam, Sovereign Base Area, Akrotiri, Bishop's Pool and Famagusta district records S.P.M. Roberts leg., G. Le Goff det., all Sovereign Base Area, Episkopi, Kensington Cliffs records S.P.M. Roberts leg., A. Müller det., all Paphos district records C. Schmid-Egger leg., A. Müller det.

**Distribution.** Cyprus, Widespread in Europe, Northern Africa, Western Asia, Central Asia, Southern Asia (India), Eastern Asia, New Zealand.

**Osmia (Pyrosmia) cephalotes Morawitz, 1870**

**References.**Mavromoustakis (1951, 1952, 1953).

**Mavromoustakis localities.** Nicosia, Apsiou, Yerasa, Trimiklini, Platania Forest Station, Kykkou Monastery, Prodromos, Rizokarpaso.

**Material examined.** Limassol District: Anogyra, 34.73663°N, 32.732715°E, 26.IV.2015, M. Jenner leg., G. Le Goff det. (1♀).

**Distribution.** Cyprus, Western Europe (France), Southern Europe, Eastern Europe, Northern Africa, Western Asia, Central Asia (Turkmenistan, Uzbekistan), Southern Asia (Iran).

**Osmia (Osmia) cornuta (Latreille, 1805)**

**References.**Mavromoustakis (1938, 1949 [“1948”], 1951, 1952).

**Mavromoustakis localities.** Apsiou, Potamitissa, Kitromili near Polemedia.

**Distribution.** Cyprus, Western Europe, Southern Europe, Eastern Europe, Northern Africa, Western Asia, Central Asia (Turkmenistan, Kazakhstan), Southern Asia (Iran).

**Notes.** The subspecies described from Cyprus is *Osmia cornuta neoregaena* Mavromoustakis, 1938, from Saittas, 762m: 34.8708333°N, 32.9166667°E, 10.III.1937, G.A. Mavromoustakis leg./det. ♀, (DAAN).

**Osmia (Pyrosmia) cyanoxantha Pérez, 1879**

**References.** Mavromoustakis (1949 [“1948”]).

**Mavromoustakis localities.** Near Limassol, Mesayitonia, Yermasoyia River, Amathus, Akrounda.

**Distribution.** Cyprus, Western Europe (France), Southern Europe, Eastern Europe (Bulgaria), Northern Africa, Western Asia, Southern Asia (Iran).

**Osmia (Helicosmia) dimidiata Morawitz, 1870**

**References.** Mavromoustakis (1949 [“1948”], 1952, 1957a).

**Mavromoustakis localities.** Limassol, Mesayitonia, Apsiou, Famagusta, Near Enkomi of Famagusta, Deryneia, Near Trimiklini.

**Distribution.** Cyprus, Western Europe (France), Southern Europe, Eastern Europe (Bulgaria, Ukraine, Russian Federation), Northern Africa, Western Asia, Central Asia (Kyrgyzstan), Southern Asia (Iran).

**Osmia (Helicosmia) dives Mocsáry, 1877**

**References.** Mavromoustakis (1949 [“1948”]).

**Mavromoustakis localities.** Limassol, Episkopi, Fassouri, Yermasoyia River, Amathus, Mesayitonia.

**Material examined.** Limassol District: Amiantos, 34.918°N, 32.9472°E, 25-27.IV.2012, (9♂), pan trap (UAEG); 1 km E of Pissouri, 34.677579°N, 32.722066°E, 27.IV.2015, (1♀), collecting pollen at *Echium angustifolium* (Boraginaceae); Cherkes, 34.641933°N, 32.963433°E, 26.IV.2018, (1♂); Akrotiri, 34.601506°N, 32.986197°E, 19.IV.2016, (1♀); Akrotiri, 34.628817°N, 32.940667°E, 30.III.2018, (1♀); Akrotiri, 34.583676°N, 32.949306°E, 14.IV.2018, (2♀); Akrotiri, 34.583676°N, 32.949306°E, 27.IV.2018, (1♀); Paphos District: 1.75 km N of Ag. Georgios, 34.911736°N, 32.327703°E, 30.IV.2015, (1♀), visiting Asteraceae, all Amiantos records S. Dimitriou leg., J. Devalez and A. Müller det., all 1 km E of Pissouri and Paphos district records S.P.M. Roberts leg., A. Müller det., all Cherkes and Akrotiri records A. Varnava leg., A. Müller det.

**Distribution.** Cyprus, Southern Europe (Greece), Eastern Europe (Hungary, Bulgaria), Northern Africa, Western Asia, Central Asia, Southern Asia (Iran).

**Osmia (Erythrosmia) erythrogastra Ferton, 1905**

**References.**Müller (2018).

**Material examined.** Limassol District: Yermasoyia Dam, 34.755799°N, 33.096194°E, 7.III.2017, Bee Course students leg., A. Müller det. (1♂).

**Distribution.** Cyprus, Western Europe (France), Southern Europe, Western Asia.

**Osmia (Pyrosmia) ferruginea Latreille, 1811**

**References.**Mavromoustakis (1939b, 1949 [“1948”], 1951, 1952).

**Mavromoustakis localities.** Cape Gata, Larnaca, Morphou, Near Deryneia.

**Material examined.** Limassol District: Yermasoyia Dam, 34.755799°N, 33.096194°E, 7.III.2017, (3♀); Yermasoyia Dam, 34.755799°N, 33.096194°E, 7.III.2017, (1♂); Polemidia, 34.71178°N, 33.004775°E, 8.III.2017, (2♀); Polemidia, 34.71178°N, 33.004775°E, 8.III.2017, (2♂, 10♀); Sovereign Base Area, Avdimou Bay Cliffs, 34.656698°N, 32.773339°E, 10.III.2017, (3♀), visiting *Onobrychis* (Fabaceae); Akrotiri, 34.601506°N, 32.986197°E, 25.III.2017, (1♂); Akrotiri, 34.583676°N, 32.949306°E, 25.III.2017, (1♂); Akrotiri, 34.600657°N, 32.971419°E, 1.III.2018, (1♂); Akrotiri, 34.5886°N, 32.9389°E, 1.III.2018, (1♀); Akrotiri, 34.628817°N, 32.940667°E, 1.III.2018, (1♂); Larnaca District: Koshi, 34.96663°N, 33.544391°E, 9.III.2018, (1♀, 1♂), visiting *Onobrychis venosa*, Yermasoyia Dam (7.III.2017, 3♀), Polemidia (8.III.2017, 2♀) and Sovereign Base Area, Avdimou Bay Cliffs records S.P.M. Roberts leg., G. Le Goff det., Yermasoyia Dam (7.III.2017, 1♂) and Polemidia (8.III.2017, 2♂, 10♀) records Bee Course students leg., A. Müller det., all Akrotiri and Larnaca district records A. Varnava leg., A. Müller det.

**Distribution.** Cyprus, Western Europe (France), Southern Europe, Northern Africa, Western Asia.

**Osmia (Pyrosmia) hellados van der Zanden, 1984**

**References.**Müller (2018).

**Material examined.** Limassol District: Yermasoyia Dam, 34.755799°N, 33.096194°E, 7.III.2017, (1♂); Polemidia, 34.71178°N, 33.004775°E, 8.III.2017, (5♂, 2♀) all records Bee Course students leg., A. Müller det.

**Distribution.** Cyprus, Southern Europe, Eastern Europe (Bulgaria), Western Asia.

**Osmia (Helicosmia) latreillei (Spinola, 1806)**

**References.**Mavromoustakis (1939b, 1951, 1952, 1957a); Georghiou (1977).

**Mavromoustakis localities.** Limassol, Cherkes, Amathus, sand dunes of Ayios Memnon (2 miles from Famagusta).

**Material examined.** Limassol District: Anogyra, 34.73663°N, 32.732715°E, 26.IV.2015, (1♀); 0.7 km N of Anogyra, 34.745537°N, 32.73385°E, 3.V.2015, (1♀); Polemidia, 34.71178°N, 33.004775°E, 8.III.2017, (6♂, 2♀); Cherkes, 34.643188°N, 32.995281°E, 25.III.2017, (1♂); Cherkes, 34.643188°N, 32.995281°E, 7.IV.2017, (1♀); Cherkes, 34.653067°N, 32.974233°E, 1.III.2018, (1♂); Cherkes, 34.659801°N, 32.99051°E, 16.III.2018, (1♀); Cherkes, 34.65096°N, 32.99091°E, 16.III.2018, (1♀); Cherkes, 34.65096°N, 32.99091°E, 30.III.2018, (1♀); Cherkes, 34.653067°N, 32.974233°E, 30.III.2018, (1♀); Paphos District: Aspro Pools, 34.720825°N, 32.551994°E, 30.IV.2015, (1♀), all Anogyra and Paphos district records M. Jenner leg., G. Le Goff det., all Polemidia records Bee Course students leg., A. Müller det., all Cherkes records A. Varnava leg., A. Müller det.

**Distribution.** Cyprus, Western Europe (France), Southern Europe, Northern Africa, Western Asia, Southern Asia (Iran).

**Osmia (Hoplosmia) ligurica Morawitz, 1868**

**References.**Mavromoustakis (1939b, 1949 [“1948”], 1951, 1952, 1953).

**Mavromoustakis localities.** Evdhimou River, Potamitissa, Yerasa, Eftagonia, Trimiklini, Arakapas, sand dunes of Ayios Memnon.

**Distribution.** Cyprus, Western Europe (France, Austria), Southern Europe, Eastern Europe, Northern Africa, Western Asia, Central Asia (Turkmenistan), Southern Asia (Iran).

**Osmia (Helicosmia) melanogaster Spinola, 1808**

**References.**Scheuchl and Willner (2016).

**Distribution.** Cyprus, Western Europe (France, Austria), Southern Europe, Eastern Europe, Northern Africa, Western Asia, Central Asia (Turkmenistan, Uzbekistan), Southern Asia (Iran), Eastern Asia (China).

**Osmia (Pyrosmia) nana Morawitz, 1874**

**References.**Mavromoustakis (1951).

**Mavromoustakis localities.** Cape Apostolos Andreas.

**Distribution.** Cyprus, Southern Europe (Italy, Croatia, Greece), Eastern Europe (Bulgaria, Russian Federation), Western Asia, Central Asia (Turkmenistan), Southern Asia (Iran).

**Osmia (Helicosmia) niveata (Fabricius, 1804)**

**References.** Mavromoustakis (1949 [“1948”]); Georghiou (1977).

**Mavromoustakis localities.** Episkopi, Potamitissa.

**Material examined.** Limassol District: Amiantos, 34.918°N, 32.9472°E, 25.iv.2012, 27.iv.2012, (2♂), pan trap (UAEG); Sovereign Base Area, Akrotiri, Bishop's Pool, 34.597305°N, 32.984521°E, 28.IV.2015, (6♀), collecting pollen on Asteraceae (Cardueae); Anogyra, 34.741952°N, 32.734845°E, 28.IV.2015, (1♂); Cherkes, 34.65096°N, 32.99091°E, 17.IV.2016, (1♀); Cherkes, 34.65096°N, 32.99091°E, 16.III.2018, (1♀); Cherkes, 34.65096°N, 32.99091°E, 14.IV.2018, (1♀); Cherkes, 34.65096°N, 32.99091°E, 26.IV.2018, (2♀); Akrotiri, 34.601506°N, 32.986197°E, 16.III.2018, (1♂), all Amiantos records S. Dimitriou leg., J. Devalez and A. Müller det., all Sovereign Base Area, Akrotiri, Bishop's Pool records S.P.M. Roberts leg., A. Müller det., all Anogyra records M. Jenner leg., G. Le Goff det., all Cherkes and Akrotiri records A. Varnava leg., A. Müller det.

**Distribution.** Cyprus, Widespread in Europe, Northern Africa, Western Asia, Southern Asia (Iran), Eastern Asia (China).

**Osmia (Pyrosmia) saxicola Ducke, 1899**

**References.**Mavromoustakis (1937b, 1939b, 1949 [“1948”], 1951, 1952, 1953).

**Mavromoustakis localities.** Near Pano Kivides, Platania Forest Station, Yerasa Hills, hills near Paramytha.

**Distribution.** Cyprus, Western Europe (France), Southern Europe, Western Asia, Central Asia (Tajikistan), Southern Asia (Iran).

**Notes.** Described from Cyprus as *Osmia cypricola* Mavromoustakis, 1937, from Pera Pedi: 34.859444°N, 32.876111°E, 20.V.1929, G.A. Mavromoustakis leg./det. ♀, (DAAN).

**Osmia (Hoplosmia) scutellaris Morawitz, 1868**

**References.**Mavromoustakis (1939b, 1949 [“1948”], 1951, 1952).

**Mavromoustakis localities.** Polemedia Hills, Akrotiri Forest, Cape Apostolos Andreas, Evdhimou River, Potamitissa, Yerasa.

**Material examined.** Limassol District: Cherkes, 34.65096°N, 32.99091°E, 9.IV.2016, (3♂); Akrotiri, 34.600657°N, 32.971419°E, 19.IV.2016, (1♀); Akrotiri, 34.600657°N, 32.971419°E, 8.V.2016, (1♀); Akrotiri, 34.600657°N, 32.971419°E, 5.V.2017, (1♂); Cherkes, 34.659801°N, 32.99051°E, 26.IV.2018, (1♀), all records A. Varnava leg., A. Müller det.

**Distribution.** Cyprus, Western Europe (France, Switzerland), Southern Europe, Eastern Europe, Northern Africa, Western Asia, Southern Asia (Iran).

**Osmia (Helicosmia) signata Erichson, 1835**

**References.** Mavromoustakis (1949 [“1948”]); Pittioni (1950); Georghiou (1977).

**Mavromoustakis localities.** Limassol, Fassouri, Randidi Forest.

**Material examined.** Paphos District: 20 km N Paphos, Kathikas, 34.90°N, 32.42°E, 20.VI.2013, (1♀); Limassol District: Cherkes, 34.65096°N, 32.99091°E, 14.IV.2018, (2♀); Cherkes, 34.65096°N, 32.99091°E, 26.IV.2018, (1♀); Akrotiri, 34.583676°N, 32.949306°E, 10.III.2017, (1♂); Akrotiri, 34.583676°N, 32.949306°E, 26.IV.2018, (3♀); Akrotiri, 34.583676°N, 32.949306°E, 28.IV.2018, (2♀), all Paphos district records C. Schmid-Egger leg., A. Müller det., all Limassol district records A. Varnava leg., A. Müller det.

**Distribution.** Cyprus, Western Europe (France), Southern Europe, Eastern Europe (Ukraine), Northern Africa, Western Asia, Central Asia (Turkmenistan), Southern Asia (Iran), Eastern Asia (China).

**Osmia (Pyrosmia) submicans Morawitz, 1870**

**References.**Mavromoustakis (1939b, 1949 [“1948”], 1951, 1957a).

**Mavromoustakis localities.** Akrotiri forest, Near Platania Forest Station, Potamitissa, Cape Apostolos Andreas, Morphou, Choirokitia, Deryneia.

**Material examined.** Limassol District: Sovereign Base Area, Akrotiri, Bishop's Pool, 34.597305°N, 32.984521°E, 28.IV.2015, (1♀); Anogyra, 34.741952°N, 32.734845°E, 28.IV.2015, (1♀); Anogyra, 34.741952°N, 32.734845°E, 3.V.2015, (1♀); Anogyra, 34.724979°N, 32.737225°E, 1.IV.2016, (1♂); Troodos, Kannoures Spring, 34.940989°N, 32.872738°E, 21.VIII.2016, (1♂); Yermasoyia Dam, 34.755799°N, 33.096194°E, 7.III.2017, (1♀); Yermasoyia Dam, 34.755799°N, 33.096194°E, 7.III.2017, (1♂); Polemidia, 34.71178°N, 33.004775°E, 8.III.2017, (1♀); Polemidia, 34.71178°N, 33.004775°E, 8.III.2017, (7♀); Akrotiri, 34.601506°N, 32.986197°E, 26.IV.2018, (1♀), all Sovereign Base Area, Akrotiri, Bishop's Pool records S.P.M. Roberts leg., A. Müller det., Anogyra records M. Jenner leg., G. Le Goff det., Anogyra (1.IV.2016, 1♂), Troodos, Kannoures Spring and Akrotiri records A. Varnava leg., A. Müller det., Yermasoyia Dam (7.III.2017, 1♀) and Polemidia (8.III.2017, 1♀) records S.P.M. Roberts leg., G. Le Goff det., Yermasoyia Dam (7.III.2017, 1♂) and Polemidia (8.III.2017, 7♀) records Bee Course students leg., A. Müller det.

**Distribution.** Cyprus, Western Europe, Southern Europe, Eastern Europe, Northern Africa, Western Asia, Central Asia (Kazakhstan).

**Osmia (Allosmia) sybarita Smith, 1853**

**References.**Mavromoustakis (1939b, 1949 [“1948”]).

**Mavromoustakis localities.** Polemedia Hills, Near Paramytha, Randidi Forest, Near Deryneia.

**Material examined.** Limassol District: Filitos, 34.800043°N, 33.000918°E, 1.IV.2016, (1♀); Akrotiri, 34.600657°N, 32.971419°E, 23.IV.2016, (1♀); Akrotiri, 34.583676°N, 32.949306°E, 16.III.2018, (1♂); Yermasoyia Dam, 34.755799°N, 33.096194°E, 7.III.2017, (1♂); Polemidia, 34.71178°N, 33.004775°E, 8.III.2017, (1♂, 3♀), all Filitos and Akrotiri records A. Varnava leg., A. Müller det., all Yermasoyia Dam and Polemidia records Bee Course students leg., A. Müller det.

**Distribution.** Cyprus, Southern Europe (Albania, Greece), Eastern Europe (Bulgaria), Western Asia.

**Osmia (Pyrosmia) teunisseni van der Zanden, 1981**

**References.** Ungricht, Müller and Dorn (2008).

**Distribution.** Cyprus, Southern Europe (Italy, Croatia, Greece), Western Asia.

**Osmia (Pyrosmia) viridana Morawitz, 1874**

**References.**Mavromoustakis (1939b, 1949 [“1948”], 1951, 1952, 1953, 1957a).

**Mavromoustakis localities.** Polemedia Hills, Trimiklini, Near Paramytha, Near Pano Kivides, Near Cape Gata, Morphou, Panagia ton Katharadon Monastery (Kyrenia).

**Material examined.** Limassol District: 0.7 km N of Anogyra, 34.745537°N, 32.73385°E, 3.V.2015, (1♀), visiting *Trifolium* (Fabaceae); Anogyra, 34.741952°N, 32.734845°E, 3.V.2015, (2♀); Anogyra to Pachna Road km 4, 34.764269°N, 32.757736°E, 5.V.2015, (2♀); Filitos, 34.800043°N, 33.000918°E, 1.IV.2016, (1♀); Polemidia, 34.71178°N, 33.004775°E, 8.III.2017, (2♂); Polemidia, 34.71178°N, 33.004775°E, 8.III.2017, (5♂, 1♀), all 0.7 km N of Anogyra and Anogyra to Pachna Road km 4 records S.P.M. Roberts leg., A. Müller det., all Anogyra records M. Jenner leg., G. Le Goff det., all Filitos records A. Varnava leg., A. Müller det., Polemidia (8.III.2017, 2♂) records S.P.M. Roberts leg., G. Le Goff det., Polemidia (8.III.2017, 5♂, 1♀) records Bee Course students leg., A. Müller det.

**Distribution.** Cyprus, Western Europe (France, Italy, Germany), Southern Europe, Eastern Europe (Bulgaria, Ukraine, Russian Federation), Northern Africa, Western Asia, Central Asia, Southern Asia (Iran).

**Notes.** The subspecies described from Cyprus is *Osmia viridana nicosiana* Mavromoustakis, 1939, from Ayia Fyla: 34.71947°N, 33.01962°E, 22.II.1937, G.A. Mavromoustakis leg., G.A. Mavromoustakis det. ♀, (DAAN).

**Genus *Protosmia* DUCKE, 1900**

3 species.

**Protosmia (Protosmia) glutinosa (Giraud, 1871)**

**References.**Mavromoustakis (1951).

**Mavromoustakis localities.** Mt. Troodos.

**Distribution.** Cyprus, Western Europe (France), Southern Europe, Eastern Europe (Bulgaria), Northern Africa, Western Asia (Turkey).

**Protosmia (Protosmia) monstrosa (Pérez, 1895)**

**References.**Mavromoustakis (1939b).

**Distribution.** Cyprus, Southern Europe (Greece), Northern Africa, Western Asia.

**Protosmia (Protosmia) paradoxa (Friese, 1899)**

**References.**Mavromoustakis (1939b, 1949 [“1948”]); Pittioni (1950).

**Mavromoustakis localities.** Cape Apostolos Andreas, Eleousa Monastery (Karpasia).

**Material examined.** Limassol District: 1 km E of Pissouri, 34.677579°N, 32.722066°E, 27.IV.2015, (1♂); Sovereign Base Area, Akrotiri, Bishop's Pool, 34.597305°N, 32.984521°E, 28.IV.2015, (1♀), collecting pollen on *Echium angustifolium* (Boraginaceae); 2 km N of Anogyra, 34.748126°N, 32.732248°E, 1.V.2015, (1♂), visiting Asteraceae; Anogyra to Pachna Road km 4, 34.764269°N, 32.757736°E, 5.V.2015, (1♂, 1♀), visiting Asteraceae; Filitos, 34.800043°N, 33.000918°E, 1.IV.2016, (2♂); Polemidia, 34.71178°N, 33.004775°E, 8.III.2017, (1♂), all 1 km E of Pissouri, Sovereign Base Area, Akrotiri Bishop's Pool, 2 km N of Anogyra and Anogyra to Pachna Road km 4 records S.P.M. Roberts leg., A. Müller det., all Filitos records A. Varnava leg., A. Müller det., all Polemidia records S.P.M. Roberts leg., G. Le Goff det.

**Distribution.** Cyprus, Southern Europe (Greece), Western Asia.

**ANTHIDIINI**

**Genus *Anthidiellum* COCKERELL, 1904**

2 Species.

**Anthidiellum (Anthidiellum) breviusculum (Mavromoustakis, 1949)**

**References.** Mavromoustakis (1949 [“1948”], 1951, 1952, 1953, 1957a).

**Mavromoustakis localities.** Mt. Troodos, Livadin of Cedars (Paphos Forest), Ayios Ilarion, Xerokolimbi (near Trooditissa), Platania Forest, Mt. Troodos Chionistra.

**Distribution.** Cyprus, Southern Europe (Spain, Greece), Eastern Europe (Bulgaria, Romania), Northern Africa (Tunisia), Western Asia (Turkey, Lebanon, Israel), Southern Asia (Iran).

**Notes.** The subspecies described from Cyprus is *Anthidiellum breviusculum troodicum* Mavromoustakis, 1949 [“1948”], from Mt. Troodos, 1524 m: 34.908°N, 32.866°E, 14.VI.1935, G.A. Mavromoustakis leg., G.A. Mavromoustakis det. ♀, (DAAN).

**Anthidiellum (Anthidiellum) strigatum (Panzer, 1805)**

**References.** Mavromoustakis (1949 [“1948”], 1951, 1952, 1957a).

**Mavromoustakis localities.** Limassol, Cherkes, Erimi, Alassa River, Chiflicoudia marshes, Livadin of Cedars (Paphos Forest), Ayia Irini (Kyrenia), Moni River, Finikaria, Symboulas, Paramali, Zakaki, Asomatos, Salipes marshes near Akrotiri.

**Distribution.** Cyprus, Widespread in Europe, Northern Africa, Western Asia, Central Asia, Southern Asia (Iran), Eastern Asia.

**Genus *Anthidium* FABRICIUS, 1804**

5 species.

**Anthidium (Anthidium) cingulatum Latreille, 1809**

**References.** Mavromoustakis (1949 [“1948”], 1952, 1957a).

**Mavromoustakis localities.** Limassol, Polemedia Hills, Akrotiri Forest and Bay, Asomatos, Yermasoyia River, Eftagonia, Lania, Cape Gata, Trimiklini, Platres, Salipes marshes, Curium (near Episkopi), Episkopi, Moni, Evdhimou River, Near Platania Forest Station, Livadin of Cedars (Paphos Forest), Mt. Troodos, Symboulas Forest, Ayia Irini Station, Kykkou Monastery, Famagusta.

**Material examined.** Limassol District: Sovereign Base Area, Episkopi, Kensington Cliffs, 34.670772°N, 32.846923°E, 4.V.2015, S.P.M. Roberts leg., J. Praz det. (1♀), visiting Lamiaceae.

**Distribution.** Cyprus, Western Europe (France, Austria, Germany), Southern Europe, Eastern Europe, Northern Africa, Western Asia, Central Asia, Southern Asia (Iran), Eastern Asia (China).

**Anthidium (Anthidium) diadema Latreille, 1809**

**References.** Mavromoustakis (1949 [“1948”], 1951, 1957a).

**Mavromoustakis localities.** Limassol, Polemedia Hills, Mesayitonia, Ayios Athanasios, Yermasoyia River, Younarka (near Zakaki), Curium (near Episkopi), Near Akhyritou, Pernera coast of Paralimni.

**Material examined.** Limassol District: Sovereign Base Area, Episkopi, Kensington Cliffs, 34.670772°N, 32.846923°E, 4.V.2015, S.P.M. Roberts leg., J. Praz det. (1♀), visiting Asteraceae.

**Distribution.** Cyprus, Western Europe (France, Belgium), Southern Europe, Eastern Europe (Bulgaria), Northern Africa (Morocco, Algeria,), Central Asia, Southern Asia (Iran), Eastern Asia (China).

**Anthidium (Anthidium) florentinum (Fabricius, 1775)**

**References.**Cockerell (1910); Mavromoustakis (1949 [“1948”], 1957).

**Mavromoustakis localities.** Ayios Ioannis (Agros), Odou, Stavros (Paphos Forest), Krios River, Zakaki.

**Distribution.** Cyprus, Western Europe, Southern Europe, Eastern Europe, Northern Africa, Western Asia, Central Asia, Southern Asia (Iran), Eastern Asia (China).

**Notes.** Described from Cyprus as *Anthidium florentinum cypriacum* Mavromoustakis, 1949 ["1949 [“1948”]"], from Agios Ioannis, 34.86777°N, 32.69027°E, VII.1930, G.A. Mavromoustakis leg., G.A. Mavromoustakis det. ♀, (DAAN).

**Anthidium (Anthidium) loti Perris, 1852**

**References.** Mavromoustakis (1949 [“1948”], 1951, 1952, 1957a); Georghiou (1977).

**Mavromoustakis localities.** Limassol, Polemedia Hills, Cherkes, Episkopi, Chiflicoudia marshes, Pera Pedi, Fassouri, Moni River, Zakaki, Episkopi Forest.

**Distribution.** Cyprus, Western Europe (France), Southern Europe, Eastern Europe (Hungary, Bulgaria, Romania), Western Asia (Turkey), Central Asia, Southern Asia (Iran).

**Anthidium (Proanthidium) undulatum Dours, 1873**

**References.**Mavromoustakis (1939a, 1949 [“1948”], 1951, 1952, 1957a).

**Mavromoustakis localities.** Limassol, Yermasoyia River, Eftagonia, Mesayitonia, Polemedia Hills, Episkopi, Erimi, Ayia Varvara (Stavrovouni), Famagusta, Glypha (near Akanthou), Mt. Troodos.

**Distribution.** Cyprus, Western Europe (France), Southern Europe (Croatia, North Macedonia, Greece), Northern Africa (Algeria), Western Asia, Southern Asia (Iran).

**Notes.** The subspecies described from Cyprus is *Anthidium undulatum holozonium* (Mavromoustakis, 1939), from Limassol: 34.66839°N, 33.03252°E, G.A. Mavromoustakis leg., G.A. Mavromoustakis det. ♀, (DAAN).

**Genus *Icteranthidium* MICHENER, 1948**

2 species.

***Icteranthidium ferrugineum* (Fabricius, 1787)**

**References.** Mavromoustakis (1949 [“1948”], 1951).

**Mavromoustakis localities.** Akrotiri Bay, Famagusta, Ayios Memnon, Yermasoyia River, Near Episkopi.

**Distribution.** Cyprus, Southern Europe (Spain), Northern Africa (Morocco, Tunisia to Egypt), Western Africa (Mauritania), Western Asia, Central Asia (Turkmenistan, Kazakhstan), Southern Asia (Iran), Eastern Asia (China).

***Icteranthidium grohmanni* (Spinola, 1838)**

**References.**Pittioni (1950); Mavromoustakis (1957a).

**Mavromoustakis localities.** Mt. Troodos, Yermasoyia River.

**Distribution.** Cyprus, Southern Europe, Eastern Europe (Bulgaria, Ukraine), Northern Africa (from Morocco to Egypt), Western Asia, Central Asia (Kyrgyzstan), Southern Asia (Iran), Eastern Asia (China).

**Genus *Rhodanthidium* ISENSEE, 1927**

1 species.

**Rhodanthidium (Rhodanthidium) septemdentatum (Latreille, 1809)**

**References.** Mavromoustakis (1949 [“1948”], 1951).

**Mavromoustakis localities.** Polemedia Hills, Northern Mountains Kantara, Near Amathus, Episkopi Forest, Symboulas Forest.

**Distribution.** Cyprus, Western Europe, Southern Europe, Eastern Europe, Western Asia.

**Genus *Eoanthidium* POPOV, 1950**

1 species.

**Eoanthidium (Eoanthidium) insulare (Morawitz, 1874)**

**References.**Mavromoustakis (1937a, 1949 [“1948”], 1951, 1952, 1953, 1957a).

**Mavromoustakis localities.** Polemedia Hills, Moni River, Evdhimou River, Mt. Troodos Kannoures springs, Mt. Troodos open slopes, Livadin of Cedars (Paphos Forest), Platania Forest Station, Ayios Ilarion (Northern Mountain), Xerokolimbi (near Trooditissa), Pasha Livadin (Mt. Troodos), Eagle’s Bath (Mt. Troodos).

**Material examined.** Limassol District: Almirolivado, 34.9333°N, 32.9004°E, 16-18.IX.2011, S. Dimitriou leg., J. Devalez det. (1♀), pan trap (UAEG).

**Distribution.** Cyprus, Southern Europe (Croatia, Greece), Western Asia (Turkey), Central Asia (Tajikistan).

**Notes.** Described from Cyprus as Dianthidium insulare var. lemesium Mavromoustakis, 1937, from Limassol: 34.66839°N, 33.03252°E, 26.VI.1931, G.A. Mavromoustakis leg./det. ♀, (DAAN).

**Genus *Stelis* PANZER, 1806**

2 species.

**Stelis (Stelis) murina Perez, 1884**

**References.**Warncke (1992); Kasparek (2015).

**Distribution.** Cyprus, Southern Europe and Northern Africa.

**Notes.** Recorded as a subspecies of *S. phaeoptera* (Kirby, 1802) by Warncke (1992). *Stelis phaeopterasensu stricto* may occur on Cyprus as depicted on the map in Kasparek (2015), but the area of overlap, hosts, and identification criteria of these forms remain uncertain as noted in text of the revision and as confirmed by its author (M. Kasparek in litt.). Pending further verification, we treat *S. phaeopterasensu stricto* as unverified in the supplementary text.

**Stelis (Protostelis) signata (Latreille, 1809)**

**References.**Popov (1944); Mavromoustakis (1949 [“1948”], 1952).

**Mavromoustakis localities.** Limassol, Polemedia Hills, Cherkes, Alassa River, Livadin of Cedars (Paphos Forest), Near Akrotiri (village), Yermasoyia River.

**Distribution.** Cyprus, Widespread in Europe, Northern Africa (Morocco, Algeria, Tunisia), Central Asia (Kazakhstan), Southern Asia (Iran).

**DIOXYINI**

**Genus *Stelis* CAMERON, 1901**

1 species.

***Stelis tridentata* (Nylander, 1848)**

**References.** Mavromoustakis (1949 [“1948”]).

**Mavromoustakis localities.** Polemedia Hills (Limassol).

**Distribution.** Cyprus, Widespread in Europe, Western Asia, Central Asia (Kyrgyzstan), Eastern Asia (China).

**Notes.** Described from Cyprus as *Dioxoides tridentata limassolica* Mavromoustakis, 1949 [“1948”]"], from Polemedia Hills, 4 miles NW of Limassol: 34.7134°N, 32.9812°E, 20.V.1939, G.A. Mavromoustakis leg./det. ♀, (DAAN).

**Genus *Dioxys* LEPELETIER & SERVILLE, 1825**

2 species.

***Dioxys cinctus* (Jurine, 1807)**

**References.**Popov (1944); Mavromoustakis (1949 [“1948”]).

**Mavromoustakis localities.** Polemedia Hills, Ayia Phyla, Sphalangiotissa Monastery.

**Distribution.** Cyprus, Western Europe (France, Switzerland, Austria), Southern Europe, Eastern Europe, Northern Africa, Western Asia (Israel), Central Asia (Uzbekistan).

***Dioxys pumilus* Gerstäcker, 1869**

**References.**Popov (1944); Mavromoustakis (1949 [“1948”], 1957a).

**Mavromoustakis localities.** Limassol, Episkopi.

**Distribution.** Cyprus, Southern Europe (Spain, Italy, Greece), Northern Africa (Morocco, Algeria, Tunisia), Western Asia (Turkey, Israel, Syria), Southern Asia (Iran).

**Notes.** Described from Cyprus as *Dioxys cypriaca* Popov, 1944, from Limassol: 34.66839°N, 33.03252°E, V.1935; IV.1936, G.A. Mavromoustakis leg., V.V. Popov det. (ZISP).

**MEGACHILINI**

**Genus *Coelioxys* LATREILLE, 1809**

12 species.

**Coelioxys (Allocoelioxys) acanthopyga Alfken, 1940**

**References.**Alfken (1940); Mavromoustakis (1952).

**Distribution.** Cyprus, Southern Europe (Greece), Western Asia (Israel).

**Notes.** Described from Cyprus as *Coelioxys carinulata* Alfken, 1940, from Cherkes: 34.65°N, 32.975°E, 29.VI.1939, G.A. Mavromoustakis leg., J.D. Alfken det. ♀, (MFNB).

**Coelioxys (Allocoelioxys) acanthura (Illiger, 1806)**

**References.** Mavromoustakis (1949 [“1948”], 1952).

**Mavromoustakis localities.** Limassol, Cherkes, Evdhimou River, Near Akrotiri.

**Distribution.** Cyprus, Western Europe (France), Southern Europe, Eastern Europe (Hungary, Ukraine), Northern Africa, Western Asia (Turkey).

**Coelioxys (Allocoelioxys) afer Lepeletier, 1841**

**References.** Mavromoustakis (1949 [“1948”], 1951, 1952, 1957a).

**Material examined.** Paphos District: 20 km N Paphos, Kathikas, 34.90°N, 32.42°E, 20.VI.2013, (2♀); Limassol District: Troodos, Mt. Olympos, 34.93°N, 32.86°E, 20.VI.2013, (2♀), all records C. Schmid-Egger leg., C. Schmid-Egger det.

**Distribution.** Cyprus, Widespread in Europe, Northern Africa (from Morocco to Tunisia), Western Asia (Turkey, Oman), Central Asia, Southern Asia (Iran).

**Coelioxys (Allocoelioxys) argenteus Lepeletier, 1841**

**References.** Mavromoustakis (1949 [“1948”], 1951, 1952).

**Mavromoustakis localities.** Limassol, Yermasoyia River, Cherkes, Polemedia Hills, Asomatos, Episkopi, Chiflicoudia marshes, Fassouri, Ayia Irini (Kyrenia), Near Akrotiri, Moni, Paramali, Near Enkomi of Famagusta.

**Distribution.** Cyprus, Western Europe (France), Southern Europe, Eastern Europe (Romania), Northern Africa, Western Asia (Turkey), Central Asia (Turkmenistan), Southern Asia (Iran), Eastern Asia (China).

**Coelioxys (Allocoelioxys) brevis Eversmann, 1852**

**References.** Mavromoustakis (1949 [“1948”]).

**Mavromoustakis localities.** Limassol, Cherkes, Near Enkomi of Famagusta.

**Distribution.** Cyprus, Western Europe (France, Germany, Austria), Southern Europe, Eastern Europe, Northern Africa, Western Asia (Turkey), Central Asia, Southern Asia (Iran), Eastern Asia (China).

**Coelioxys (Melissoctonia) conoideus (Illiger, 1806)**

**References.** Mavromoustakis (1949 [“1948”], 1952, 1957a).

**Mavromoustakis localities.** Limassol, Cherkes, Potamitissa, Odou, Erimi, Trooditissa Monastery.

**Distribution.** Cyprus, Widespread in Europe, Northern Africa, Western Asia (Turkey), Southern Asia (Iran).

**Coelioxys (Liothyrapis) decipiens Spinola, 1838**

**Remarks.** Reported by Mavromoustakis under its junior synonym *Paracoelioxys decipiens*.

**References.** Mavromoustakis (1949 [“1948”]); Pittioni (1950); Mavromoustakis (1952, 1957).

**Mavromoustakis localities.** Limassol, Erimi, Cherkes, Famagusta.

**Distribution.** Cyprus, Southern Europe (Greece), Northern Africa, Western Asia (Turkey), Central Asia, Southern Asia (Iran, India), Eastern Asia (China).

**Coelioxys (Allocoelioxys) echinatus Förster, 1853**

**References.**Scheuchl and Willner (2016).

**Distribution.** Cyprus, Western Europe, Southern Europe, Eastern Europe, Northern Africa (Morocco, Algeria, Egypt), Western Asia (Turkey, Israel, Iraq), Southern Asia (Iran, Afghanistan).

**Coelioxys (Allocoelioxys) elegantulus Alfken, 1934**

**References.** Mavromoustakis (1949 [“1948”]); Pittioni (1950); Mavromoustakis (1952, 1957a).

**Mavromoustakis localities.** Limassol, Polemedia Hills, Cherkes, Chiflicoudia marshes, Yermasoyia River.

**Distribution.** Cyprus, Southern Europe (Greece), Northern Africa (Algeria, Egypt), Western Asia (Turkey, Israel, Palestine).

**Coelioxys (Allocoelioxys) haemorrhoa Förster, 1853**

**References.**Alfken (1940); Mavromoustakis (1949 [“1948”]); Pittioni (1950); Mavromoustakis (1952).

**Mavromoustakis localities.** Limassol, Cherkes, Asomatos, Chiflicoudia marshes.

**Distribution.** Cyprus, Western Europe (Austria), Southern Europe (Spain, Greece), Eastern Europe (Romania), Northern Africa, Western Asia (Turkey, Israel), Central Asia, Southern Asia (Iran, Pakistan, India), Eastern Asia (China).

**Coelioxys (Paracoelioxys) inermis Kirby, 1802**

**References.**Scheuchl and Willner (2016).

**Distribution.** Cyprus, Widespread in Europe, Northern Africa (Morocco, Algeria), Western Asia (Turkey), Central Asia (Uzbekistan, Kyrgyzstan), Southern Asia (Iran), Eastern Asia (China).

**Coelioxys (Allocoelioxys) polycentris Förster, 1853**

**References.**Pittioni (1950).

**Distribution.** Cyprus, Western Europe (France, Austria), Southern Europe, Eastern Europe, Southern Asia (Iran), Southern Asia (Iran, Pakistan), Eastern Asia (Mongolia).

**Genus *Megachile* LATREILLE, 1802**

17 species.

**Megachile (Creightonella) albisecta (Klug, 1817)**

**References.**Cockerell (1931); Mavromoustakis (1939b, 1949 [“1948”]); Pittioni (1950); Mavromoustakis (1951, 1954, 1957a).

**Mavromoustakis localities.** Limassol, Polemedia Hills, Cherkes, Erimi, Evdhimou River, Pera Pedi, Pissouri, Ayia Irini (Kyrenia), Near Akrotiri village, Yermasoyia River, Moni River, Pernera coast of Paralimni, Younarka (near Zakaki), Near Ayios Theodoros (Pitsillia).

**Material examined.** Paphos District: 20 km N Paphos, Kathikas, 34.90°N, 32.42°E, 20.VI.2013, C. Schmid-Egger leg., C. Praz det. (1♀, 1♂).

**Distribution.** Cyprus, Western Europe (France, Switzerland), Southern Europe, Eastern Europe, Northern Africa, Western Asia, Central Asia, Eastern Asia (China).

**Notes.** The subspecies described from Cyprus is *Megachile albisecta cyprica* Cockerell, 1931, from Limassol: 34.66839°N, 33.03252°E, 15-17.VII.1928, G.A. Mavromoustakis leg., T.D.A. Cockerell det. ♀.

**Megachile (Eutricharaea) anatolica Rebmann, 1968**

**References.**Soltani et al. (2017).

**Distribution.** Cyprus, Southern Europe (Italy, Greece), Western Asia, Southern Asia (Iran).

**Megachile (Eutricharaea) apicalis Spinola, 1808**

**References.**Mavromoustakis (1939b, 1949 [“1948”]); Pittioni (1950); Mavromoustakis (1951, 1952, 1957a).

**Mavromoustakis localities.** Limassol, Polemedia Hills, Cherkes, Near Akrotiri, Paramali, Davlos coast, Tsada, Chiflicoudia marshes, Livadin of Cedars (Paphos Forest), Krios River (Kaledonia Falls), Glypha (near Akanthou), Younarka (near Zakaki), Near Mesayitonia, Ayios Memnon near Famagusta, Younaros of Zakaki, Akrotiri (village), Ayios Ilarion, Mt. Pentadactylos, Garillis River, Pachna.

**Material examined.** Paphos District: 15 km SE Paphos, Kouklia, 34.72°N, 32.55°E, 20.VI.2013, (1♀, 2♂); 20 km NNW Paphos, Lara Beach, 34.94°N, 32.31°E, 20.VI.2013, (1♀); 6 km W Polis, botanical garden, 35.03°N, 32.37°E, 20.VI.2013, (1♀); Limassol District: 8 km S Limassol, Akrotiri (near Airbase), 34.60°N, 32.97°E, 20.VI.2013, C. (1♀, 1♂), all records Schmid-Egger leg., C. Praz det.

**Distribution.** Cyprus, Widespread in Europe, Western Asia, Central Asia (Uzbekistan), Southern Asia (Iran, Pakistan), Eastern Asia (China).

**Megachile (Megachile) centuncularis (Linnaeus, 1758)**

**References.**Mavromoustakis (1953, 1954, 1957a, b).

**Mavromoustakis localities.** Limassol, Cherkes, Famagusta, Platania Forest Station, Platres, Pera Pedi, Kilani, Odou, Yermasoyia River, Chiflicoudia marshes, Eagle’s Bath (Mt. Troodos).

**Distribution.** Cyprus, Widespread in Europe, Northern Africa, Western Asia, Central Asia (Kazakhstan), Southern Asia (Iran, India).

**Notes.** Described from Cyprus as *Megachile centuncularis nesiotica* Mavromoustakis, 1953, from Limassol: 34.66839°N, 33.03252°E, VIII.1936, G.A. Mavromoustakis leg., G.A. Mavromoustakis det. ♀, (DAAN).

**Megachile (Chalicodoma) cypricola Mavromoustakis, 1938**

**Type locality–country.** Cyprus, Ayia Phyla: 34.7198°N, 33.0195°E, 23.III.1938, G.A. Mavromoustakis leg., G.A. Mavromoustakis det. ♀, (DAAN).

**References.**Mavromoustakis (1938, 1949 [“1948”], 1951, 1952, 1957a).

**Mavromoustakis localities.** Apsiou, Near Pano Kivides, Yerasa Hills, Choirokitia, Ayia Napa.

**Material examined.** Limassol District: Sovereign Base Area, Avdimou Bay Cliffs, 34.656698°N, 32.773339°E, 27.IV.2015, (1♀); Sovereign Base Area, Avdimou Bay Cliffs, 34.656698°N, 32.773339°E, 27.IV.2015, (1♀); 3.5 km SE of Moni, 34.706873°N, 33.211916°E, 14.III.2017, (2♂, 1♀), visiting *Onobrychis venosa* (Fabaceae); 3.5 km SE of Moni, 34.706873°N, 33.211916°E, 14.III.2017, (2♀, 1♂), visiting *Onobrychis venosa* (Fabaceae); 0.5 km W of Agios Georgios, 34.706786°N, 33.229364°E, 14.III.2017, (1♀); 0.5 km W of Agios Georgios, 34.706786°N, 33.229364°E, 14.III.2017, (2♀); Agios Georgios, 34.706786°N, 33.229364°E, 27.II.2018, (1♀, 3♂), visiting *Onobrychis venosa* (Fabaceae); Avdimou Bay Cliffs, 34.656698°N, 32.773339°E, 17.III.2018, (5♀), visiting *Onobrychis venosa* (Fabaceae); Paramali, 34.66198333°N, 32.80439444°E, 17.III.2018, (3♀), visiting *Onobrychis venosa* (Fabaceae); Larnaca District: 2.1 km S of Choirokoitia, 34.777521°N, 33.33622°E, 14.III.2017, (1♂); Choirokitia, 34.777521°N, 33.33622°E, 4.III.2018, (1♀), Sovereign Base Area, Avdimou Bay Cliffs records M. Jenner leg., C. Praz det. and S.P.M. Roberts leg., C. Praz det., 3.5 km SE of Moni (14.III.2017, 2♂, 1♀), 0.5 km W of Agios Georgios (14.III.2017, 1♀) and 2.1 km S of Choirokoitia records S.P.M. Roberts leg., S.P.M. Roberts det., 3.5 km SE of Moni (14.III.2017, 2♀, 1♂) and 0.5 km W of Agios Georgios (14.III.2017, 2♀) records A. Varnava leg., S.P.M. Roberts det., Agios Georgios, Avdimou Bay Cliffs, Paramali, and Choirokitia (4.III.2018) records A. Varnava leg., A. Varnava det.

**Distribution. Cyprus. ENDEMIC.**

**Megachile (Pseudomegachile) ericetorum (Lepeletier, 1841)**

**References.**Georghiou (1977).

**Material examined.** Limassol District: Yermasoyia Dam, 34.745849°N, 33.083579°E, 28.X.2016, S.P.M. Roberts leg., G. Le Goff det. (1♀), visiting *Dittrichia viscosa* (Asteraceae).

**Distribution.** Cyprus, Western Europe, Southern Europe, Eastern Europe, Northern Europe (Finland), Northern Africa, Western Asia, Southern Asia (Iran), Eastern Asia (China).

**Megachile (Pseudomegachile) farinosa Smith, 1853**

**References.**Dorchin and Praz (2018).

**Material examined.** Paphos District: 15 km SE Paphos, Kouklia, 34.72°N, 32.55°E, 20.VI.2013, (1♂); 8 km N Paphos, Mavrokolympos Reservoir, 34.85°N, 32.40°E, 20.VI.2013, (3♂), all records C. Schmid-Egger leg., C. Praz det.

**Distribution.** Cyprus, Southern Europe (Greece), Western Asia (Turkey, Syria, Iraq), Southern Asia (Iran).

**Megachile (Eutricharaea) inexspectata Rebmann, 1968**

**Material examined.** Paphos District: 6 km NE Polis, beach 35.06°N, 32.46°E, 20.VI.2013, C. Schmid-Egger leg., C. Praz det. (2♂).

**Distribution.** Cyprus, Northern Africa (Morocco, Algeria, Tunisia), Western Asia.

**Megachile (Eutricharea) leachella Curtis, 1828**

**References.**Georghiou (1977).

**Material examined.** Paphos District: 15 km SE Paphos, Kouklia, 34.72°N, 32.55°E, 20.VI.2013, (4♀, 3♂); 20 km N Paphos, Kathikas, 34.90°N, 32.42°E, 20.VI.2013, (1♂); 20 km NNW Paphos, Lara Beach, 34.94°N, 32.31°E, (12♀, 7♂); 6 km W Polis, botanical garden, 35.03°N, 32.37°E, 20.VI.2013, (1♀); 8 km N Paphos, Mavrokolympos Reservoir, 34.85°N, 32.40°E, 20.VI.2013, (2♀, 1♂); Polis, 35.053539°N, 32.351197°E, 30.X.2016, (1♂), visiting *Dittrichia viscosa* (Asteraceae); Limassol District: Troodos, Mt. Olympos, 34.93°N, 32.86°E, (1♂); Yermasoyia Dam, 34.745849°N, 33.083579°E, 28.X.2016, (1♂, 4♀), visiting *Dittrichia viscosa* (Asteraceae); Sovereign Base Area, Akrotiri, 34.600657°N, 32.971419°E, 29.X.2016, (1♀), visiting *Dittrichia viscosa* (Asteraceae); Sovereign Base Area, Paramali, 34.676011°N, 32.794947°E, 30.X.2016, (1♂), visiting *Dittrichia viscosa* (Asteraceae); Larnaca District: Zygi, 34.731233°N, 33.343487°E, 28.X.2016, (1♀), visiting *Dittrichia viscosa* (Asteraceae); Famagusta District: Achna Dam, 35.05519°N, 33.814011°E, 28.X.2016, (2♂, 1♀), visiting *Dittrichia viscosa* (Asteraceae), all 15 km SE Paphos Kouklia, 20 km N Paphos Kathikas, 20 km NNW Paphos Lara Beach, 6 km W Polis botanical garden, 8 km N Paphos Mavrokolympos Reservoir and Troodos Mt. Olympos records C. Schmid-Egger leg., C. Praz det., Polis, Yermasoyia Dam, Sovereign Base Area Akrotiri, Sovereign Base Area Paramali, Zygi, and Achna Dam records S.P.M. Roberts leg., S.P.M. Roberts and G. Le Goff det.

**Distribution.** Cyprus, Widespread in Europe, Central Asia.

**Megachile (Eutricharaea) marginata Smith, 1853**

**References.**Mavromoustakis (1939b).

**Material examined.** Paphos District: 15 km SE Paphos, Kouklia, 34.72°N, 32.55°E, 20.VI.2013, C. Schmid-Egger leg., C. Praz det. (2♀, 2♂).

**Distribution.** Cyprus, Southern Europe, Northern Africa (Tunisia), Western Asia (Turkey, Iraq), Central Asia (Kyrgyzstan, Tajikistan), Southern Asia (Afghanistan, Pakistan).

**Megachile (Megachile) melanopyga Costa, 1863**

**References.**Mavromoustakis (1957a); Georghiou (1977).

**Mavromoustakis localities.** Platania Forest Station, Odou, Yermasoyia River, Near Zakaki.

**Distribution.** Cyprus, Western Europe, Southern Europe (Spain, Italy, Slovenia), Eastern Europe, Western Asia, Eastern Asia (China).

**Notes.** Described from Cyprus as *Megachile melanopyga zakakica* Mavromoustakis, 1957, from Platania Forest Station, Mt. Troodos: 34.9473°N, 32.9284°E, 5.IX.1951, G.A. Mavromoustakis leg., G.A. Mavromoustakis det. ♀.

**Megachile (Eurymella) patellimana Spinola, 1838**

**References.**Pittioni (1950); Mavromoustakis (1951, 1952).

**Mavromoustakis localities.** Famagusta, Near Akrotiri, Moni River, Akrotiri (village), Ayios Memnon.

**Material examined.** Paphos District: 20 km NNW Paphos, Lara Beach, 34.94°N, 32.31°E, 20.VI.2013, (10♀, 7♂); 6 km W Polis, botanical garden, 35.03°N, 32.37°E, 20.VI.2013, (1♂); 8 km N Paphos, Mavrokolympos Reservoir, 34.85°N, 32.40°E, 20.VI.2013, (1♂), all records C. Schmid-Egger leg., C. Praz det.

**Distribution.** Cyprus, Western Africa (Niger, Nigeria), Southern Africa (Namibia, Botswana), Northern Africa (Egypt, Sudan), Eastern Africa (Mozambique), Western Asia (Israel, Saudi Arabia, United Arab Emirates), Southern Asia (Pakistan).

**Megachile (Eutricharea) pilidens Alfken, 1890**

**References.**Cockerell (1931).

**Material examined.** Limassol District: Amiantos, 34.918°N, 32.9472°E, 15-17.IX.2011, S. Dimitriou leg., J. Devalez det. (1♀), pan trap (UAEG).

**Distribution.** Cyprus, Northern Africa (Morocco), Western Europe, Southern Europe, Eastern Europe, Central Asia.

**Megachile (Eutricharaea) posti Mavromoustakis, 1952**

**Type locality–country.** Cyprus, Polemedia Hills: 34.699444°N, 32.996944°E, 5.VII.1949, G.A. Mavromoustakis leg., G.A. Mavromoustakis det. ♀, (DAAN).

**References.**Mavromoustakis (1952).

**Mavromoustakis localities.** Polemedia Hills.

**Distribution. Cyprus. ENDEMIC.**

**Notes.** The status of this species has long been in question but Praz (2017) is of the opinion that *M. posti* is a good species and distinct from *M. basilaris* Morawitz, 1875, with which it has been synonymised in the past.

**Megachile (Chalicodoma) roeweri (Alfken, 1928)**

**References.**Mavromoustakis (1939b, 1949 [“1948”], 1952, 1954).

**Mavromoustakis localities.** Limassol, Pera Pedi, Akrotiri (village).

**Distribution.** Cyprus, Southern Europe (Greece), Western Asia (Turkey).

**Notes.** The subspecies described from Cyprus is *Megachile roeweri akrotirica* Mavromoustakis, 1939, from Akrotiri Forest: 34.5843°N, 32.9676°E, 23.V.1938, G.A. Mavromoustakis leg., G.A. Mavromoustakis det. ♀, (DAAN).

**Megachile (Eutricharaea) rotundata (Fabricius, 1787)**

**References.**Mavromoustakis (1939b, 1952).

**Mavromoustakis localities.** Chiflicoudia marshes, Erimi, Salipes marshes near Akrotiri.

**Distribution.** Cyprus, Widespread in Europe, Northern Africa, Western Asia (Turkey, Georgia, Azerbaijan), Central Asia, Southern Asia (Iran, Pakistan, India), Eastern Asia (China, Mongolia), Australia, New Zealand.

**Megachile (Paracella) troodica Mavromoustakis, 1953**

**References.**Mavromoustakis (1953); Zanden (1992).

**Mavromoustakis localities.** Mt. Troodos, Chionistra, Xerokolimbi.

**Distribution. Cyprus. ENDEMIC.**

**Notes.** Described from Cyprus as Megachile (Eutricharaea) mavromoustakisi van der Zanden, 1992, from Mt. Troodos, 1676m (open slopes in the forest of *Pinus nigra pallasiana* Lamb.): 34.9234°N, 32.8833°E, 7.VII.1936, G.A. Mavromoustakis leg., G.A. Mavromoustakis det. ♀ (DAAN).

**APIDAE**

**ANTHOPHORINI**

**Genus *Amegilla* FRIESE, 1897**

4 species.

**Amegilla (Zebramegilla) albigena (Lepeletier, 1841)**

**References.** Mavromoustakis (1949 [“1948”], 1951).

**Mavromoustakis localities.** Limassol, Polemedia Hills, Ayios Athanasios, Tsada, Mt. Troodos, Chionistra, Kato Platres, Ayios Ioannis Agrou, Famagusta, Stroumbi, Kathikas.

**Material examined.** Nicosia District: Kakopetria, 34.992°N, 32.9082°E, 15-17.IX.2011, (1♂), pan trap (UAEG); Limassol District: Almirolivado, 34.9333°N, 32.9004°E, 16-18.IX.2011, (4♀, 3♂), pan trap (UAEG), all records S. Dimitriou leg., J. Devalez det.

**Distribution.** Cyprus, Western Europe (France, Switzerland, Austria), Southern Europe, Eastern Europe, Northern Africa, Western Asia, Central Asia, Southern Asia (Iran, Pakistan), Eastern Asia (China).

**Amegilla (Micramegilla) glauca (Alfken, 1926)**

**References.** Mavromoustakis (1949 [“1948”]); Pittioni (1950); Mavromoustakis (1951, 1952, 1957a).

**Mavromoustakis localities.** Chiflicoudia marshes, Akrotiri Bay and Forest, Cherkes, Famagusta, Paramali.

**Distribution.** Cyprus, Northern Africa (Egypt).

**Amegilla (Amegilla) quadrifasciata** (de Villers, 1789)

**References.** Mavromoustakis (1949 [“1948”], 1951, 1952, 1957a); Georghiou (1977).

**Mavromoustakis localities.** Limassol, Pissouri, Akrotiri Bay, Fasouri, Polemedia, Cherkes, Asomatos, Chiflicoudia marshes, Moni, Eftagonia, Evdhimou River, Platania Forest Station, Famagusta, Mt. Troodos, Chionistra, Kannoures springs, Kathikas, Livadin of Cedars (Paphos Forest), Akanthou, Ayia Irini (Kyrenia district).

**Material examined.** Limassol District: Almirolivado, 34.9333°N, 32.9004°E, 16-18.IX.2011, S. Dimitriou leg., J. Devalez det. (1♀), pan trap (UAEG).

**Distribution.** Cyprus, Widespread in Europe, Northern Africa, Western Asia, Central Asia, South-Eastern Asia (Myanmar), Eastern Asia.

***Amegilla*** (***Zebramegilla***) ***salviae* (Morawitz, 1876)**

**References.**Scheuchl and Willner (2016).

**Mavromoustakis localities.** Limassol.

**Distribution.** Cyprus, Western Europe (Switzerland, Austria), Southern Europe, Eastern Europe, Northern Africa, Western Asia (Turkey, Azerbaijan), Central Asia, Southern Asia (Iran), Eastern Asia (China).

**Genus *Anthophora* LATREILLE, 1803**

8 species.

**Anthophora (Anthophora) canescens Brullé, 1832**

**References.** Lepeletier (1841); Dours (1869); Costa (1883); Mavromoustakis (1949 [“1948”], 1951, 1952, 1953, 1954, 1957a); Georghiou (1977).

**Mavromoustakis localities.** Limassol, Polemedia, hills, Episkopi, Ayios Athanasios, Apsiou, Pera Pedi, Akrotiri Bay, Fassouri, Yermasoyia River, Pissouri, Fasoula, Yerasa, Pyrgos, Near Paramytha, Moni, Kellaki, Zakaki, Saettas, Morphou, Amathus, Kyrenia, Famagusta, Nicosia.

**Material examined.** Nicosia District: 35.1688°N, 33.367°E, 13.III.2006, A Grace leg., J. Devalez det. (2♂), hand net (UAEG); Paphos District: 34.7626°N, 32.4108°E, 25.II.2000, 8.III.2000, H. Wolf leg., J. Devalez det. (2♀), hand net (UAEG).

**Distribution.** Cyprus, Southern Europe, Western Europe (France, Switzerland), Northern Africa, Western Asia.

**Notes.** This is the taxon sometimes referred to as *Anthophora subterranea* Germar, 1826.

**Anthophora (Pyganthophora) dalmatica Pérez, 1902**

**Material examined.** Limassol District: Almirolivado, 34.9333°N, 32.9004°E, 14.V.2012, 16.V.2012, S. Dimitriou leg., J. Devalez det. (1♀), pan trap, (UAEG); Nicosia District: 35.1688°N, 33.367°E, 13.III.2006, A. Grace leg., A. Grace det. (2♀), insect net, (UAEG).

**Distribution.** Cyprus, Southern Europe.

**Notes.** The true status of this taxon is not certain and Rasmont (in litt.) suggests that it could be a ssp. of *A. atroalba* Lepeletier, 1841. Whatever the status, the taxon is newly recognised in Cyprus.

**Anthophora (Paramegilla) harmalae Morawitz, 1877**

**References.** Mavromoustakis (1949 [“1948”], 1951).

**Mavromoustakis localities.** Limassol, Tsada, Krios River near Kilani, Mt. Troodos Kannoures springs.

**Distribution.** Cyprus, Eastern Europe (Russian Federation), Northern Africa, Western Asia, Central Asia, Southern Asia (Iran).

**Anthophora (Anthophora) plumipes (Pallas, 1772)**

**References.** Mavromoustakis (1949 [“1948”], 1957); Georghiou (1977).

**Mavromoustakis localities.** Limassol, Yermasoyia River, Cherkes, Fassouri, Trimiklini, Episkopi, Kilani, Amathus, Fasoula, Yerasa, Fassouri, Pera Pedi, Potamitissa, Pyrgos, Near Paramytha, Saettas, Kellaki, Ayios Kostantinos Pitsilia, Kyrenia, Ayios Ilarion, Nicosia.

**Material examined.** Nicosia District: Kakopetria, 34.992°N, 32.9082°E, 5-7.IV.2012, (1♀), pan trap (UAEG); Makria Kontarka, 34.9095°N, 32.8971°E, 14-16.V.2012, (1♀), pan trap (UAEG), all records S. Dimitriou leg., J. Devalez det.

**Distribution.** Cyprus, Widespread in Europe, Northern Africa, Western Asia, Central Asia (Kazakhstan), Southern Asia (Iran, Afghanistan).

**Notes.** Described from Cyprus as *Anthophora acervorum cypriaca* Mavromoustakis, 1957, from Amathus ruins: 34.7125°N, 33.1419°E, 27.II.1935, G.A. Mavromoustakis leg./det. ♂.

**Anthophora (Lophanthophora) robusta (Klug, 1845)**

**References.** Mavromoustakis (1949 [“1948”]); Pittioni (1950); Mavromoustakis (1951, 1957a).

**Mavromoustakis localities.** Limassol, Polemedia Hills, Episkopi, Ayios Athanasios, Pera Pedi, Kato Platres, Mt. Troodos Chionistra, Curium (near Episkopi), Trimiklini, Ayios Athanasios, Mt. Troodos Pasha Livadin.

**Distribution.** Cyprus, Western Europe (France), Southern Europe, Eastern Europe (Bulgaria, Romania, Ukraine), Northern Africa (Morocco, Algeria), Western Asia, Central Asia (Turkmenistan, Kazakhstan).

**Anthophora (Pyganthophora) rogenhoferi Morawitz, 1871**

**References.** Mavromoustakis (1949 [“1948”], 1951, 1952, 1953, 1954, 1957a); Georghiou (1977).

**Mavromoustakis localities.** Limassol, Mesayitonia, Akrotiri Forest, Episkopi, Apsiou, Kantara Mountains, Kaloiri Hills, Kellaki, Pera Pedi, Fassouri, Amathus, Fasoula, Yerasa, Pyrgos, Larnaca, Nicosia, Karpasian Peninsula, Eleousa Monastery, Morphou, Ayios Ilarion, Randidi Forest, Rizokarpaso.

**Distribution.** Cyprus, Southern Europe (Italy, Greece), Western Asia, Central Asia, Southern Asia (Iran).

**Anthophora (Pyganthophora) rubricrus Dours, 1870**

**References.** Mavromoustakis (1949 [“1948”], 1951, 1952, 1953, 1954, 1957a).

**Mavromoustakis localities.** Limassol, Polemedia Hills, Mesayitonia, Akrotiri Forest/Bay, Apsiou, Pera Pedi, Fassouri, Yermasoyia River, Amathus, Fasoula, Yerasa, Pyrgos, Near Paramytha, Akrounda, Kellaki, Lania, Pareklisia, Trimiklini, Morphou, Amathus, Kyrenia, Famagusta.

**Material examined.** Nicosia District: 35.1688°N, 33.367°E, 13.III.2006, A. Grace leg., J. Devalez det. (5♂, 8♀), insect net (UAEG); Paphos District: 34.7626°N, 32.4108°E, 25.II.2000, 8.III.2000, H. Wolf leg., J. Devalez det. (4♀), insect net (UAEG).

**Distribution.** Cyprus, Southern Europe (Greece), Western Asia (Israel).

**Anthophora (Lophanthophora) rutilans Dours, 1870**

**Type locality–country.** Cyprus, Nicosia: 35.166667°N, 33.366667°E, Sichel leg., Dours collection, J.A. Dours det. ♂ (MNHN).

**References.**Dours (1870); Mavromoustakis (1949 [“1948”], 1951, 1952, 1953, 1954, 1957a).

**Mavromoustakis localities.** Limassol, Polemedia, Kitromili near Polemedia, near Ayios Athanasios, Cherkes, Akrotiri Forest, Mesayitonia, Episkopi, Fassouri, Yermasoyia River, Pera Pedi, Amathus, Potamitissa, Fassoula, Yerasa, Kellaki, Moni, Near Paramytha, Amyrou Monastery (near Apsiou), Ayios Ilarion, Near Famagusta, Morphou, Mesayitonia-Fasoula.

**Material examined.** Paphos District: 34.7626°N, 32.4108°E, 25.II.2000, 8.III.2000, H. Wolf leg., J. Devalez det. (1♂), insect net (UAEG).

**Distribution.** Cyprus, Southern Europe (Greece), Eastern Europe (Russian Federation), Western Asia.

**AMMOBATINI**

**Genus *Ammobates* LATREILLE, 1809**

3 species.

***Ammobatoides abdominalis* (Eversmann, 1852)**

**References.**Scheuchl and Willner (2016).

**Distribution.** Cyprus, Western Europe (Austria), Southern Europe, Eastern Europe, Western Asia (Turkey, Lebanon), Central Asia (Kazakhstan), Southern Asia (Iran), Eastern Asia (China).

**Ammobates (Ammobates) biastoides Friese, 1895**

**References.**Mavromoustakis (1954).

**Mavromoustakis localities.** Cherkes.

**Distribution.** Cyprus, Northern Africa (Algeria), Western Asia (Turkey, Israel).

**Notes.** The subspecies described from Cyprus is *Ammobates biastoides globosus* Mavromoustakis, 1954, from Cherkes: 34.65°N, 32.975°E, 16.VI.1939, G.A. Mavromoustakis leg., G.A. Mavomoustakis det. ♀ (DAAN).

**Ammobates (Ammobates) mavromoustakisi Popov, 1944**

**References.**Popov (1944); Mavromoustakis (1949 [“1948”], 1951, 1954).

**Mavromoustakis localities.** Limassol, Cherkes, Zakaki, Ayios Athanasios, Farangas near Famagusta, Paramali, Yermasoyia.

**Distribution.** Cyprus, Western Asia (Turkey, Palestine, Israel), Central Asia (Uzbekistan, Tajikistan), Southern Asia (Iran).

**Notes.** The subspecies described from Cyprus is *Ammobates mavromoustakisi mavromoustakisi*, from Limassol, Ayios Athanasios, Cherkes: 34.72075°N, 33.05327°E, 34.65635°N, 32.98748°E, G.A. Mavromoustakis leg., V.V. Popov. det. (ZISP).

**Genus *Chiasmognathu* s ENGEL, 2006**

1 species.

***Chiasmognathus orientanus* (Warncke, 1983)**

**References.**Warncke (1983).

**Distribution.** Cyprus, Southern Europe (Greece), Eastern Europe (Bulgaria, Romania), Western Asia (Turkey, Palestine, Israel).

**Notes.** Described from Cyprus as Pasites (Parammobatodes) orientanus cyprius Warncke, 1983, from Yermasoyia River: 34.7182°N, 33.08788°E, 27.VII.1967, G.A. Mavromoustakis leg., K. Warncke det. ♀, (KW).

All records of *Ch. orientanus* from Cyprus have been assigned *cyprius*.

**Genus *Parammobatodes* POPOV, 1931**

1 species.

***Parammobatodes minutus* (Mocsáry, 1878)**

**References.**Scheuchl and Willner (2016).

**Distribution.** Cyprus, Western Europe (Austria), Southern Europe (Greece), Eastern Europe, Western Asia.

**Genus *Pasites* JURINE, 1807**

1 species.

***Pasites maculatus* Jurine, 1807**

**References.**Cockerell (1910); Popov (1944); Mavromoustakis (1949 [“1948”], 1951, 1952, 1957a).

**Mavromoustakis localities.** Limassol, Polemedia Hills, Cherkes, Moni River, Farangas near Famagusta, Ayia Napa.

**Distribution.** Cyprus, Western Europe (France, Switzerland, Austria), Southern Europe, Eastern Europe, Northern Africa, Western Asia, Central Asia, Southern Asia (Iran, Afghanistan, Pakistan), Eastern Asia (China, Mongolia).

**ANCYLAINI**

**Genus *Ancyla* LEPELETIER, 1841**

1 species.

***Ancyla holtzi* Friese, 1902**

**References.** Mavromoustakis (1949 [“1948”]).

**Mavromoustakis localities.** Limassol, Cherkes, Zakaki, Asomatos, Near Enkomi of Famagusta.

**Distribution.** Cyprus, Southern Europe (Greece), Eastern Europe (Bulgaria), Western Asia (Turkey, Iraq), Southern Asia (Iran).

**Genus *Tarsalia* MORAWITZ, 1895**

2 species.

***Tarsalia ancyliformis* Popov, 1935**

**References.**Pittioni (1950); Mavromoustakis (1952, 1953).

**Mavromoustakis localities.** Limassol, Cherkes, Yermasoyia River, Moni, Salipes marshes near Akrotiri, Yermasoyia Hills, Near Asomatos, Near Trachoni.

**Distribution.** Cyprus, Southern Europe (Italy), Western Asia (Turkey), Central Asia (Turkmenistan, Uzbekistan, Tajikistan), Southern Asia (Iran).

**Notes.** Described from Cyprus as *Tarsalia ancyliformis mediterranea* Pittioni, 1950, from Geroskipou: 34.7559°N, 32.4516°E, 20.VII.1939, H. Lindberg leg., B. Pittioni det.

Includes *T. mediterranea* which is now regarded as a ssp. of *Tarsalia ancyliformis* (MZHF).

***Tarsalia hirtipes* Morawitz, 1894**

**References.**Mavromoustakis (1952, 1953, 1957a).

**Mavromoustakis localities.** Limassol, Cherkes.

**Distribution.** Cyprus, Western Asia (Turkey), Central Asia (Turkmenistan, Uzbekistan), Southern Asia (Iran).

**Notes.** Described from Cyprus as *Tarsalia hirtipes cypriaca* Mavromoustakis, 1952, from Cherkes: 34.65°N, 32.975°E, 3.VIII.1933, G.A. Mavromoustakis leg./det. ♀, (DAAN). Includes *T. cypriaca* which is now regarded as a ssp. of *Tarsalia hirtipes*.

**APINI**

**Genus *Apis* LINNAEUS, 1758**

1 species.

**Apis (Apis) mellifera Linnaeus, 1758**

**References.**Pittioni (1950); Georghiou (1977).

**Distribution.** Cyprus, Widespread in Europe, Africa, Western Asia, Central Asia, Southern Asia, Australia, New Zealand.

**Notes.** The subspecies described as *Apis mellifera cypria* Pollmann, 1879, from unknown location, det. A. Pollmann. Beekeeping is widely practised throughout the island.

**BOMBINI**

**Genus *Bombus* LATREILLE, 1802**

2 species.

**Bombus (Sibiricobombus) niveatus Kriechbaumer, 1870**

**References.** Rasmont and Iserbyt (2014); Rasmont et al. (2015).

**Distribution.** Cyprus, Southern Europe (Greece, Albania), Eastern Europe (Bulgaria, Ukraine), Western Asia (Lebanon, Syria), Southern Asia (Iran), Eastern Asia (China).

**Notes.** Lack of recent records for Cyprus indicates the need for a conservation assessment of its status on the island.

**Bombus (Bombus) terrestris (Linnaeus, 1758)**

**References.** Mavromoustakis (1949 [“1948”]); Pittioni (1950); Georghiou (1977).

**Material examined.** Nicosia District: Linou, 35.0755°N, 32.9164°E, 14-16.V.2012, (1♀), pan trap (UAEG); Kakopetria, 34.992°N, 32.9082°E, 15-17.IX.2011, (2♀), pan trap (UAEG); Kakopetria, 34.992°N, 32.9082°E, 5-7.IV.2012, (2♀), pan trap (UAEG); Kakopetria, 34.992°N, 32.9082°E, 14-16.V.2012, (2♀), pan trap (UAEG); Limassol District: Amiantos, 34.918°N, 32.9472°E, 14-16.V.2012, (1♀), pan trap (UAEG); Amiantos, 34.918°N, 32.9472°E, 31.V.2012, 2.VI.2012, (1♂, 3♀), pan trap (UAEG); Almirolivado, 34.9333°N, 32.9004°E, 16-18.IX.2011, (2♀), pan trap (UAEG); Almirolivado, 34.9333°N, 32.9004°E, 14-16.V.2012, (29♀), pan trap (UAEG); Almirolivado, 34.9333°N, 32.9004°E, 31.V.2012, 2.VI.2012, (6♀), pan trap (UAEG); Makria Kontarka, 34.9095°N, 32.8971°E, 16-18.IX.2011, (3♀), pan trap (UAEG); Makria Kontarka, 34.9095°N, 32.8971°E, 14-16.V.2012, (15♀) (UAEG); Makria Kontarka, 34.9095°N, 32.8971°E, 31.V.2012, 2.VI.2012, (2♂, 1♀), pan trap (UAEG); Troodos, Chionistra, 34.9317°N, 32.8664°E, 14-16.V.2012, (3♂, 18♀), pan trap (UAEG); Troodos, Chionistra, 34.9317°N, 32.8664°E, 16-18.IX.2011, (1♂), pan trap (UAEG); Troodos, Chionistra, 34.9317°N, 32.8664°E, 31.V.2012, 2.VI.2012, (12♀), pan trap (UAEG); Troodos, Mt. Olympos, 34.93°N, 32.86°E, 20.VI.2013, (6♀); Sovereign Base Area, Avdimou Bay Cliffs, 34.656698°N, 32.773339°E, 27.IV.2015; Sovereign Base Area, Episkopi, Kensington Cliffs, 34.670772°N, 32.846923°E, 4.V.2015, all Linou, Kakopetria, Amiantos, Almirolivado, Makria Kontarka and Troodos Chionistra records S. Dimitriou leg., J. Devalez det., Troodos Mt. Olympos records C. Schmid-Egger leg., C. Schmid-Egger det., Sovereign Base Area, Avdimou Bay Cliffs and Sovereign Base Area, Episkopi, Kensington Cliffs records S.P.M. Roberts leg., S.P.M. Roberts det.

**Distribution.** Cyprus, Widespread in Europe, Northern Africa, Central Asia (Kazakhstan, Kyrgyzstan, Uzbekistan), Southern Asia (Iran, Afghanistan), Eastern China (China, Mongolia), Australia, New Zealand.

**CERATININI**

**Genus *Ceratina* LATREILLE, 1802**

8 species.

**Ceratina (Neoceratina) bispinosa Handlirsch, 1889**

**References.** Mavromoustakis (1949 [“1948”]); Pittioni (1950); Mavromoustakis (1952, 1957a).

**Mavromoustakis localities.** Limassol, Cherkes, Eftagonia, Zakaki, Ayios Athanasios.

**Distribution.** Cyprus, Southern Europe (Croatia, Greece), Eastern Europe (Romania), Western Asia.

**Ceratina (Euceratina) chrysomalla Gerstäcker, 1869**

**References.** Mavromoustakis (1949 [“1948”]); Mavromoustakis (1951, 1952, 1954, 1957a,b).

**Mavromoustakis localities.** Limassol, Cherkes, Apsiou, Pera Pedi, Moni, Yermasoyia River/Hills (Kaloiri), Yerasa, Lania, Ayia Varvara (Stavrovouni), Platres, Pyrga.

**Distribution.** Cyprus, Southern Europe (Greece), Eastern Europe (Romania, Ukraine, Bulgaria), Western Asia, Southern Asia (Iran).

**Ceratina (Euceratina) cypriaca Mavromoustakis, 1949**

**Type locality–country.** Cyprus, Pera Pedi: 609 m, 34.8580°N, 32.8730°E, 27.V.1929, G.A. Mavromoustakis leg., G.A. Mavromoustakis det. ♀, (DAAN).

**References.** Mavromoustakis (1949 [“1948”]); Pittioni (1950); Mavromoustakis (1951, 1952, 1953, 1957a).

**Distribution. Cyprus. ENDEMIC.**

**Ceratina (Euceratina) dallatorreana Friese, 1896**

**References.** Mavromoustakis (1949 [“1948”]).

**Mavromoustakis localities.** Polemedia Hills, Apsiou, Livadin of Cedars (Paphos Forest), Mt. Troodos, Trimiklini, Eftagonia, Pera Pedi, Kykkou Monastery, Stavrovouni, Mesapotamos, Xerokolimbi (near Trooditissa), Potamitissa.

**Distribution.** Cyprus, Western Europe (France), Southern Europe, Eastern Europe, Northern Africa (Morocco, Algeria, Tunisia), Western Asia, Central Asia (Turkmenistan, Kyrgyzstan, Uzbekistan), Southern Asia (Iran).

**Ceratina (Euceratina) mandibularis Friese, 1896**

**References.** Mavromoustakis (1949 [“1948”]); Pittioni (1950); Mavromoustakis (1951, 1952, 1953, 1954, 1958 ["1957"]).

**Mavromoustakis localities.** Cherkes, Chiflicoudia marshes, Yermasoyia River, Yerasa, Trimiklini, Erimi, Kathikas.

**Material examined.** Paphos District: 2.7 km SW of Acheleia, Potamos tis Ezouzas, 34.729004°N, 32.457544°E, 30.IV.2015, S.P.M. Roberts leg./det. (3♂).

**Distribution.** Cyprus, Northern Africa (Egypt), Western Asia (Turkey).

**Ceratina (Euceratina) moricei Friese, 1899**

**References.** Mavromoustakis (1949 [“1948”], 1952, 1957a).

**Mavromoustakis localities.** Limassol, Cherkes, Asomatos, Yermasoyia River, Pyrgos, Trimiklini, Yermasoyia Hills.

**Distribution.** Cyprus, Western Asia (Turkey), Southern Asia (Iran).

**Ceratina (Dalyatina) parvula Smith, 1854**

**References.** Mavromoustakis (1949 [“1948”]); Pittioni (1950); Mavromoustakis (1951).

**Mavromoustakis localities.** Limassol, Yermasoyia River.

**Distribution.** Cyprus, Western Europe (France), Southern Europe, Eastern Europe (Bulgaria), Northern Africa, Western Asia, Central Asia (Turkmenistan).

**Ceratina (Neoceratina) schwarzi Kocourek, 1998**

**References.**Terzo (1998).

**Distribution.** Cyprus, Southern Europe, Eastern Europe (Romania, Bulgaria), Western Asia, Southern Asia (Iran).

**EPEOLINI**

**Genus *Epeolus* LATREILLE, 1802**

2 species.

***Epeolus bischoffi* (Ma**﻿**vromoustakis, 1954)**

**References.**Bogusch and Hadrava (2018).

**Mavromoustakis localities.** Mavromoustakis specimens in the Snow Entomological Museum Collection, Kansas, USA: Akrotiri Bay, 34.62°N, 33.00°E, 12.VII.1943; Zakaki, 23.VI.1949; Salamis, 18.VI.1957; Akrotiri Bay, 34.62°N, 33.00°E, 20.VII.1933; Akrotiri Bay, 34.62°N, 33.00°E, 1.VIII.1933; Akrotiri Bay, 34.62°N, 33.00°E, 12.VII.1943, all records G.A. Mavromoustakis leg./det.

**Distribution.** Cyprus, Western Asia.

***Epeolus transitorius* Eversman**﻿**n, 1852**

**References.**Bogusch and Hadrava (2018).

**Distribution.** Cyprus, Western Europe (France), Southern Europe, Eastern Europe (Russian Federation), Northern Africa (Morocco), Western Asia, Central Asia (Turkmenistan, Kazakhstan, Uzbekistan), Southern Asia (Iran).

**EUCERINI**

**Genus *Eucera* SCOPOLI, 1770**

21 species.

**Eucera (Hetereucera) aequata Vachal, 1907**

**Material examined.** Limassol District: Akrotiri, 34.600657°N, 32.971419°E, 8.V.2016, (1♀); Akrotiri, 34.600657°N, 32.971419°E, 7.IV.2017, (2♂); Akrotiri, 34.600657°N, 32.971419°E, 21.IV.2017, (1♀, 2♂); Akrotiri, 34.600657°N, 32.971419°E, 5.V.2017, (1♀); Akrotiri, 34.600657°N, 32.971419°E, 30.III.2018, (2♂); Akrotiri, 34.600657°N, 32.971419°E, 28.IV.2019 (1♀), all records A. Varnava leg., A. Dorchin det.

**Distribution.** Cyprus, Western Asia (Turkey, Israel, Syria).

**Eucera (Tetralonia) alticincta (Lepeletier, 1841**﻿)

**References.** Mavromoustakis (1949 [“1948”]).

**Mavromoustakis localities.** Odou, Krios River near Kilani.

**Distribution.** Cyprus, Widespread in Europe except in the North, Northern Africa, Western Asia (Turkey).

**Eucera (Hetereucera) bidentata Pérez, 1887**

**References.** Mavromoustakis (1949 [“1948”], 1951, 1952, 1957a); Georghiou (1977).

**Mavromoustakis localities.** Limassol, Mesayitonia, Fassouri, Yermasoyia River, Nicosia, Famagusta, Amathus, Fasoula, Morphou, Rizokarpaso, Ayia Irini station (near Limassol).

**Material examined.** Kyrenia District: 15 km E Kyrenia, 'Turtle Beach', 35.334413°N, 33.494187°E, 10.IV.2013, (1♀); Famagusta District: Protaras, Cape Greco, 34.963264°N, 34.066211°E, 15.III.2017, (1♀); Achna Dam, 35.05519°N, 33.814011°E, 19.II.2018, (1♂); Limassol District: Cherkes, 34.653067°N, 32.974233°E, 10.III.2017, (1♂); Cherkes, 34.641933°N, 32.963433°E, 25.III.2017, (1♂); Cherkes, 34.653067°N, 32.974233°E, 25.III.2017, (1♂); Cherkes, 34.653067°N, 32.974233°E, 14.II.2018, (2♂); Cherkes, 34.653067°N, 32.974233°E, 1.III.2018, (2♂); Cherkes, 34.650960°N, 32.990910°E, 1.III.2018, (1♂); Cherkes, 34.641933°N, 32.963433°E, 1.III.2018, (1♂); Cherkes, 34.650960°N, 32.990910°E, 30.III.2018, (1♀); Akrotiri, 34.601506°N, 32.986197°E, 25.III.2017, (1♂); Akrotiri, 34.628817°N, 32.940667°E, 25.III.2017, (1♂); Akrotiri, 34.601506°N, 32.986197°E, 1.III.2018, (1♂), all Kyrenia district records C. Schmid-Egger leg., S. Risch det., all Famagusta and Limassol district records A. Varnava leg., A. Dorchin det.

**Distribution.** Cyprus, Southern Europe (Italy, Greece), Western Asia (Turkey, Israel, Syria).

**Eucera (Hetereucera) caerulescens Friese, 1899**

**References.** Mavromoustakis (1949 [“1948”], 1951, 1952, 1957a).

**Mavromoustakis localities.** Limassol, Polemedia Hills, Mesayitonia, Cherkes, Apsiou, Cape Apostolos Andreas, Yermasoyia River, Yerasa, Pyrgos, Akrounda, Pareklisia, Randidi Forest.

**Material examined.** Kyrenia District: 5 km E of Kyrenia, "Turtle Beach", 35.334413°N, 33.494187°E, 10.IV.2007; Famagusta District: 15 km E of Rizokarpaso, "Golden Sands", 35.64°N, 34.55°E, 10.IV.2007, 1 (♀) (AMNH), all records C. Schmid-Egger leg., S. Risch det.

**Distribution.** Cyprus, Western Asia (Turkey, Israel), Southern Asia (Iran).

**Eucera (Eucera) cypria Alfken, 1933**

**Type locality–country.** Cyprus, Limassol: 34.66839°N, 33.03252°E, 13-27.II.1927, G.A. Mavromoustakis leg., J.D. Alfken det.

**References.**Alfken (1933); Mavromoustakis (1949 [“1948”], 1951, 1953, 1954, 1957a); Tkalců (1984).

**Mavromoustakis localities.** Limassol, Polemedia Hills, Mesayitonia, Pera Pedi, Fassouri, Yermasoyia River, Famagusta, Amathus, Yerasa, Larnaca, Near Paramytha, Mesayitonia-Fasoula, Moni, Lania, Trimiklini.

**Material examined.** Limassol District: Anogyra, 34.724979°N, 32.737225°E, 23.II.2016, (1♂); Cherkes, 34.650960°N, 32.990910°E, 1.III.2018, (1♂), all records A. Varnava leg., A. Dorchin det.

**Distribution.** Cyprus, Southern Europe (Greece), Western Asia (Israel), Southern Asia (Iran).

**Eucera (Eucera) dalmatica Lepeletier, 1841**

**References.** Mavromoustakis (1949 [“1948”], 1952, 1957a); Georghiou (1977).

**Mavromoustakis localities.** Limassol, Akrotiri Forest, Near Enkomi of Famagusta, Younarka, Pyrga, Xylophagou, Ormideia, Near Deryneia, Ayios Memnon (Famagusta).

**Material examined.** Limassol District: Akrotiri, 34.628817°N, 32.940667°E, 24.V.2017, A. Varnava leg., A. Dorchin det. (1♂).

**Distribution.** Cyprus, Western Europe (France), Southern Europe, Eastern Europe, Northern Africa (Morocco), Western Asia, Southern Asia (Iran).

**Eucera (Eucera) dimidiata Brullé, 1832**

**References.** Mavromoustakis (1949 [“1948”], 1951, 1952, 1953, 1954, 1957a); Georghiou (1977).

**Mavromoustakis localities.** Limassol, Polemedia Hills, Episkopi, Fassouri, Yermasoyia River, Mesayitonia, Fasoula, Pera Pedi, Amathus, Trimiklini, Kyrenia, Morphou, Famagusta, Sandy shore of Salamis (near Famagusta), Larnaca.

**Material examined.** Nicosia District: city centre, 35.1688°N, 33.367°E, 13.III.2006, (5♂, 16♀), insect net (UAEG); Limassol District: Anogyra, 34.724979°N, 32.737225°E, 23.II.2016, (1♀, 3♂); Ayios Dimitrianos, 8.III.2016, (1♂); Aswmatos, 8.III.2016, (1♂); Pissouri, 34.65093056°N, 32.72539722°E, 5.II.2017, (1♂); Cherkes, 34.641933°N, 32.963433°E, 23.II.2017, (1♂); Cherkes, 34.650960°N, 32.990910°E, 25.III.2017, (2♀); Cherkes, 34.641933°N, 32.963433°E, 25.III.2017, (1♀); Cherkes, 34.659801°N, 32.990510°E, 25.III.2017, (3♀); Cherkes, 34.641933°N, 32.963433°E, 21.IV.2017, (2♀); Cherkes, 34.641933°N, 32.963433°E, 1.II.2018, (1♂); Cherkes, 34.653067°N, 32.974233°E, 14.II.2018, (1♂); Cherkes, 34.641933°N, 32.963433°E, 1.III.2018, (1♀); Cherkes, 34.659801°N, 32.990510°E, 1.III.2018, (1♀); Cherkes, 34.659801°N, 32.990510°E, 16.III.2018, (2♀); Akrotiri, 34.588600°N, 32.938900°E, 1.III.2018, (1♀); Akrotiri, 34.628817°N, 32.940667°E, 1.III.2018, (1♂); Larnaca District: Choirokitia, 34.777521°N 33.33622°E, 27.II.2018, (1♀); Famagusta District: Deryneia, 23.III.2016, (1♀); Protaras, Cape Greco, 34.963264°N, 34.066211°E, 5.ii.2018, (1♂); Achna Dam, 35.05519°N, 33.814011°E, 19.II.2018, (1♀), Nicosia district records A. Grace leg., S. Risch det., all Limassol, Larnaca, and Famagusta district records A. Varnava leg., A. Dorchin det.

**Distribution.** Cyprus, Southern Europe (Greece), Northern Africa (Algeria, Tunisia, Egypt), Western Asia (Turkey, Israel, Saudi Arabia), Southern Asia (Iran, Afghanistan).

**Eucera (Hetereucera) furfurea Vachal, 1907**

**References.**Alfken (1933); Mavromoustakis (1949 [“1948”]).

**Mavromoustakis localities.** Limassol, Akrotiri Forest, Pissouri, Nicosia.

**Distribution.** Cyprus, Southern Europe (Greece), Western Asia (Turkey, Israel).

**Eucera (Hetereucera) gaullei Vachal, 1907**

**References.**Alfken (1933); Mavromoustakis (1949 [“1948”], 1951); Georghiou (1977).

**Mavromoustakis localities.** Limassol, Akrotiri Forest, Episkopi, Yermasoyia River, Kato Platres, Potamitissa, Kyrenia, Sphalangiotissa Monastery, Northern Mountains Kantara, Near Salamis, Ayios Ilarion.

**Material examined.** Nicosia District: city centre, 35.1688°N, 33.367°E, 13.III.2006, (4♂), insect net (UAEG); Limassol District: Cherkes, 34.641933°N, 32.963433°E, 8.V.2016, (1♀); Akrotiri, 34.601506°N, 32.986197°E, 21.IV.2017, (1♀); Akrotiri, 34.600657°N, 32.971419°E, 26.IV.2018, (1♀), all Nicosia district records A. Grace leg., S. Risch det., all Limassol district records A. Varnava leg., A. Dorchin det.

**Distribution.** Cyprus, Western Asia.

**Eucera (Tetralonia) glauca (Fabricius, 1775)**

**References.**Pittioni (1950); Mavromoustakis (1952, 1957a); Tkalců (1979).

**Mavromoustakis localities.** Pyrga (Larnaca).

Mavromoustakis specimens in the Snow Entomological Museum Collection, Kansas, USA: Asomatos, 34.64°N, 32.96°E, 26.VI.1951, G.A. Mavromoustakis leg./det., (1♀) and 3 unspecified specimens.

**Distribution.** Cyprus, Southern Europe (Greece), Western Asia (Turkey, Iraq), Southern Asia (Iran).

**Eucera (Tetralonia) inulae (Tkalců, 1979)**

**References.**Tkalců (1979).

**Mavromoustakis localities.** Mavromoustakis specimens in the Snow Entomological Museum Collection, Kansas, USA: Limassol, Kilani, 14.VII.1937, G.A. Mavromoustakis leg., D.B. Baker det., 3 specimens.

**Material examined.** Limassol District: Akrotiri; 34.600657°N, 32.971419°E, 29.X.2016, S.P.M. Roberts leg./det., foraging on *Dittrichia viscosa* (Asteraceae), (1♂, 1♀).

**Distribution.** Cyprus, Southern Europe, Eastern Europe (Ukraine, Bulgaria, Russian Federation), Southern Asia (Iran).

**Eucera (Synhalonia) mavromoustakisi (Tkalců, 1984)**

**Type locality–country.** Cyprus, Lania: 34.82444°N, 32.92083°E, 2.VI.1964, G.A. Mavromoustakis leg., B. Tkalců det. ♂ (FSAG).

**References.**Tkalců (1984).

**Distribution. Cyprus. ENDEMIC.**

**Eucera (Tetralonia) malvae (Rossi, 1790)**

**References.** Mavromoustakis (1949 [“1948”]).

**Mavromoustakis localities.** Polemedia Hills, Mesayitonia, Akrotiri Forest, Symboulas Chiflik (near Limassol), Near Platania Forest Station, Lania, Ayia Varvara (Stavrovouni), Near Ayios Athanasios, hills near Trimiklini.

**Distribution.** Cyprus, Widespread across Europe, Western Asia (Turkey).

**Eucera (Eucera) palaestinae Friese, 1922**

**Material examined.** Limassol District: Akrotiri, 34.601506°N, 32.986197°E, 21.IV.2017, (1♂); Akrotiri, 34.601506°N, 32.986197°E, 1.III.2018, (3♀); Akrotiri, 34.600657°N, 32.971419°E, 1.III.2018, (2♀), all Limassol district records A. Varnava leg., A. Dorchin det.

**Distribution.** Cyprus, Western Asia, Southern Asia (Iran).

**Eucera (Eucera) proxima Morawitz, 1875**

**References.** Mavromoustakis (1949 [“1948”], 1951, 1952).

**Material examined.** Limassol District: Troodos, Chionistra, 34.9317°N, 32.8664°E, 31.V.2012, 2.VI.2012, (1♂), pan trap, (UAEG); Akrotiri, 34.628817°N, 32.940667°E, 25.III.2017, (1♀); Akrotiri, 34.601506°N, 32.986197°E, 7.IV.2017, (1♀); Akrotiri, 34.601506°N, 32.986197°E, 21.IV.2017, (1♀); Akrotiri, 34.628817°N, 32.940667°E, 21.IV.2017, (1♀); Akrotiri, 34.600657°N, 32.971419°E, 1.III.2018, (1♂), all Troodos, Chionistra records S. Dimitriou leg., S. Risch det., all Akrotiri records A. Varnava leg., A. Dorchin det.

**Distribution.** Cyprus, Western Europe (France), Southern Europe, Eastern Europe, Western Asia (Turkey, Syria), Central Asia (Turkmenistan, Uzbekistan, Tajikistan), Southern Asia (Iran).

**Notes.** Also recorded as *E. graeca* Radoszkowski, 1876 e.g., Rasmont et al. 2017. Precedence is given to *proxima* following Nieto et al. (2014; see methodology).

**Eucera (Eucera) seminuda Brullé, 1832**

**References.**Scheuchl and Willner (2016).

**Distribution.** Cyprus, Western Europe (France, Austria), Southern Europe (North Macedonia, Italy, Greece), Eastern Europe, Northern Africa (Tunisia), Western Asia (Turkey, Armenia).

**Eucera (Eucera) sinufascia Dorchin, 2018**

**References.**Tkalců (1979).

**Material examined.** Limassol District: Cherkes, 34.641933°N, 32.963433°E, 10.III.2017, A. Varnava leg., A. Dorchin det. (1♂).

**Distribution.** Cyprus, Southern Europe (Greece (Aegean islands)), Western Asia.

**Notes.** Synonymising *Tetraloniella* under *Eucera*, the name became a junior homonym of Eucera (Tetralonia) penicillata (Friese, 1905) and *Eucera sinufascia* Dorchin 2018 was proposed as a replacement Dorchin et al. (2018).

**Eucera (Eucera) sulamita Vachal, 1907**

**Material examined.** Northern Cyprus, 10 km N of Famagusta, Salamis, 35.187°N, 33.899°E, 4.X.2007, C. Schmid-Egger leg., S. Risch det. (1♂) (AMNH).

**Distribution.** Cyprus, Western Asia (Israel).

**Eucera (Hetereucera) syriaca Dalla Torre, 1896**

**References.** Mavromoustakis (1949 [“1948”], 1951, 1957a); Georghiou (1977).

**Mavromoustakis localities.** Limassol, Polemedia Hills, Mesayitonia, Ayia Phyla, Nicosia, Sphalangiotissa Monastery, Fassouri, Famagusta, Amathus, Near Enkomi of Famagusta.

**Material examined.** Famagusta District: 5 km E of Rizokarpaso, 35.63°N, 34.50°E, 10.IV.2007,; Limassol District: Akrotiri, 34.583676°N, 32.949306°E, 14.IV.2018, (1♀); Akrotiri, 34.583676°N, 32.949306°E, 28.IV.2018, (1♀), all Famagusta district records C. Schmid-Egger leg., S. Risch det., all Limassol district records A. Varnava leg., A. Dorchin det.

**Distribution.** Cyprus, Western Asia, Southern Asia (Iran).

**Eucera (Synhalonia) tricincta Erichson, 1835**

**References.**Tkalců (1984).

**Distribution.** Cyprus, Western Europe (Austria), Southern Europe (Spain, Italy, Greece), Eastern Europe, Northern Africa, Western Asia.

**Eucera (Synhalonia) zeta Dalla Torre, 1896**

**References.**Mavromoustakis (1952).

**Mavromoustakis localities.** Pera Pedi, Potamitissa, Moni, Kellaki, Saettas, Kitromili, Mt. Kornos.

**Distribution.** Cyprus, Southern Europe (Greece), Western Asia, Central Asia (Turkmenistan), Southern Asia (Iran).

**Notes.** Described from Cyprus as *Eucera vernalis sintenisi* Mavromoustakis, 1952 from, Potamitissa: 34.9075°N, 32.989444°E, 16.III.1947, G.A. Mavromoustakis leg., G.A. Mavromoustakis det. ♀, (DAAN).

**MELECTINI**

**Genus *Melecta* LATREILLE, 1802**

7 species.

**Melecta (Melecta) albifrons (Forster, 1771)**

**References.** Mavromoustakis (1949 [“1948”], 1951); Lieftinck (1980).

**Mavromoustakis localities.** Nicosia, Mt. Troodos, Yermasoyia River, Amathus, Potamitissa, Yerasa, Near Amathus, Prodromos, Dragontospillios cave.

**Distribution.** Cyprus, Widespread in Europe, Northern Africa, Western Asia, Southern Asia (Iran).

**Melecta (Melecta) duodecimmaculata (Rossi, 1790)**

**References.** Mavromoustakis (1949 [“1948”]); Georghiou (1977); Lieftinck (1980).

**Mavromoustakis localities.** Polemedia Hills, Mesayitonia, Limassol, Ayia Phyla, Fassouri, Yermasoyia River, Amathus, Yerasa.

**Distribution.** Cyprus, Western Europe (France), Southern Europe, Northern Africa, Western Asia, Central Asia, Southern Asia (Iran).

**Melecta (Melecta) italica Radoszkowski, 1876**

**References.**Lieftinck (1980).

**Distribution.** Cyprus, Western Europe (France), Southern Europe, Northern Africa, Western Asia, Eastern Asia.

**Melecta (Melecta) leucorhyncha Gribodo, 1894**

**References.**Lieftinck (1980).

**Distribution.** Cyprus, Western Europe (France), Southern Europe, Eastern Europe (Ukraine), Northern Africa, Western Asia, Eastern Asia.

**Melecta (Melecta) luctuosa (Scopoli, 1770)**

**References.**Scheuchl and Willner (2016).

**Distribution.** Cyprus, Widespread in Europe, Northern Africa, Western Asia, Central Asia, Southern Asia (Iran, Pakistan).

**Melecta (Melecta) mundula Lieftinck, 1983**

**References.**Lieftinck (1983); Schwarz (1999).

**Distribution.** Cyprus, Southern Europe (Greece), Eastern Europe (Russian Federation), Western Asia (Turkey, Israel, Jordan), Central Asia, Southern Asia (Iran).

**Notes.** Described from Cyprus as *Melecta megaera* Lieftinck, 1980, from Polemedia: 34.699444°N, 32.996944°E, 9.III.1950, G.A. Mavromoustakis leg., M.A. Lieftinck det. ♂, (MFNB).

**Melecta (Melecta) tuberculata Lieftinck, 1980**

**References.**Lieftinck (1980).

**Distribution.** Cyprus, Western Europe (France), Southern Europe (Portugal, Spain, Greece), Eastern Europe (Bulgaria, Russian Federation), Western Asia (Turkey, Israel, Lebanon), Eastern Asia.

**Genus *Thyreus* PANZER, 1806**

5 species.

***Thyreus affinis* (Morawitz, 1874)**

**References.**Pittioni (1950); Lieftinck (1968); Mavromoustakis (1951, 1952).

**Mavromoustakis localities.** Limassol, Polemedia Hills, Akrotiri Bay, Near Akrotiri, Younaros of Zakaki.

**Distribution.** Cyprus, Western Europe (France), Southern Europe, Eastern Europe, Northern Africa, Western Asia, Central Asia, Southern Asia (Iran, Pakistan).

***Thyreus elegans* (Morawitz, 1877)**

**References.**Lieftinck (1968).

**Distribution.** Cyprus, Southern Europe, Northern Africa, Western Asia, Central Asia (Turkmenistan, Kyrgyzstan), Southern Asia (Iran, Pakistan).

***Thyreus histrionicus* (Illiger, 1806)**

**References.** Mavromoustakis (1949 [“1948”], 1951, 1952, 1957a); Lieftinck (1968); Georghiou (1977).

**Mavromoustakis localities.** Limassol, Polemedia Hills, Mesayitonia, Cherkes, Akrotiri Bay, Near Akrotiri, Near Enkomi of Famagusta, Yermasoyia River, Akrounda, Moni River, Mt. Troodos Chionistra, Pernera coast of Paralimni, Younarka (near Zakaki), Krios River near Kilani, near Fassouri.

**Material examined.** Paphos District: 20 km NNW Paphos, Lara Beach, 34.94°N, 32.31°E, 20.VI.2013, C. Schmid-Egger leg., M. Schwarz det. (1♀).

**Distribution.** Cyprus, Western Europe (France, Austria), Southern Europe, Eastern Europe, Northern Africa, Western Asia, Central Asia, Southern Asia (Iran), Eastern Asia (China).

***Thyreus picaron* Lieftinck, 1968**

**Material examined.** Paphos District: 20 km NNW Paphos, Lara Beach, 34.94°N, 32.31°E, 20.VI.2013, C. Schmid-Egger leg., M. Schwarz det. (1♂).

**Distribution.** Cyprus, Western Europe (France), Southern Europe, Eastern Europe (Bulgaria, Romania).

***Thyreus ramosus* (Lepeletier, 1841)**

**References.** Mavromoustakis (1949 [“1948”], 1951, 1952, 1954); Lieftinck (1968); Georghiou (1977).

**Mavromoustakis localities.** Limassol, Cherkes, Episkopi, Ayios Athanasios, Pera Pedi, Akrotiri Bay, Moni, Near Akrotiri, Yermasoyia River, Eftagonia, Xerokolimbi Stream near Trooditissa, Platres, Pera Pedi.

**Material examined.** Paphos District: 20 km NNW Paphos, Lara Beach, 34.94°N, 32.31°E, 20.VI.2013, C. Schmid-Egger leg., M. Schwarz det. (1♂).

**Distribution.** Cyprus, Western Europe (France, Switzerland, Austria), Southern Europe, Eastern Europe, Northern Africa, Western Asia, Central Asia, Southern Asia, Eastern Asia (China).

**NOMADINI**

**Genus *Nomada* SCOPOLI, 1770**

39 species.

***Nomada babiyi* Schwarz & Standfuss, 2007**

**References.**Smit (2018).

**Distribution.** Cyprus, Southern Europe (Croatia, Greece), Eastern Europe (Ukraine, Bulgaria), Western Asia (Turkey, Syria).

***Nomada bifasciata* Olivier, 1812**

**References.**Smit (2018).

**Distribution.** Cyprus, Widespread in Europe, Northern Africa, Western Asia (Turkey).

***Nomada caspia* Morawitz, 1894**

**References.**Smit (2018).

**Distribution.** Cyprus, Southern Europe (Croatia, Greece), Western Asia (Turkey, Israel, Lebanon), Central Asia (Turkmenistan).

***Nomada cherkesiana* Mavromoustakis, 1955**

**Type locality–country.** Cyprus, Cherkes: 34.65°N, 32.975°E, 30.III.1950, G.A. Mavromoustakis leg., G.A. Mavromoustakis det. ♀, (DAAN).

**References.**Mavromoustakis (1955, 1957a); Smit (2018).

**Mavromoustakis localities.** Cherkes, Yermasoyia River, Yerasa.

**Distribution.** Cyprus, Southern Europe (Greece), Western Asia (Turkey, Israel).

***Nomada confinis* Schmiedeknecht, 1882**

**References.**Smit (2018).

**Distribution.** Cyprus, Western Europe (Switzerland), Southern Europe, Eastern Europe (Bulgaria), Western Asia, Southern Asia (Iran).

***Nomada cypria* Mavromoustakis, 1952**

**Type locality–country.** Cyprus, Cherkes: 34.65°N, 32.975°E, 15.III.1950, G.A. Mavromoustakis leg., G.A. Mavromoustakis det. ♀, (DAAN).

**References.**Schwarz (1999); Mavromoustakis (1952); Smit (2018).

**Distribution.** Cyprus, Southern Europe (Greece), Western Asia (Israel).

***Nomada cypricola* Mavromoustakis, 1955**

**Type locality–country.** Cyprus, Zakaki: 34.6563°N 33.0029°E, 17.VI.1949, G.A. Mavromoustakis leg., G.A. Mavromoustakis det. ♀, (DAAN).

**References.**Mavromoustakis (1957a); Smit (2018).

**Mavromoustakis localities.** Limassol, Polemedia Hills, Ayia Irini (near Palodkia), Sphalangiotissa Monastery (near Limassol).

**Distribution. Cyprus. ENDEMIC.**

***Nomada erythrocephala* Morawitz, 1871**

**References.** Mavromoustakis (1949 [“1948”]); Smit (2018).

**Mavromoustakis localities.** Limassol, Polemedia Hills, Episkopi Forest, Ayia Irini Station (near Limassol).

**Distribution.** Cyprus, Western Europe (France), Southern Europe (Spain, Greece), Eastern Europe, Western Asia (Turkey).

***Nomada filicornis* Schwarz & Smit, 2018**

**References.**Smit (2018).

**Remarks.** Smit paratype localities: Limassol, Vavla, Akrotiri, Kapedhes.

**Distribution.** Cyprus, Southern Europe (Italy, Greece), Western Asia (Turkey, Jordan, Syria).

***Nomada flavinervis* Brullé, 1832**

**References.**Mavromoustakis (1955, 1957a); Smit (2018).

**Mavromoustakis localities.** Limassol, Pera Pedi, Lania, Kellaki, Fasoula, Trimiklini, Episkopi, Yermasoyia River.

**Distribution.** Cyprus, Southern Europe (Greece), Eastern Europe (Bulgaria), Western Asia (Turkey, Israel).

***Nomada flavoguttata* (Kirby, 1802)**

**References.** Mavromoustakis (1949 [“1948”]); Smit (2018).

**Mavromoustakis localities.** Limassol.

**Material examined.** Limassol District: Yermasoyia Dam, 34.755799°N, 33.096194°E, 7.III.2017, Bee Course students leg., M. Schwarz det. (4♀).

**Distribution.** Cyprus, Widespread in Europe, Northern Africa, Western Asia, Central Asia, Southern Asia (Iran), Eastern Asia (China).

***Nomada fucata* Panzer, 1798**

**References.** Mavromoustakis (1949 [“1948”]); Georghiou (1977); Smit (2018).

**Mavromoustakis localities.** Limassol, Yermasoyia River, Amathus, Potamitissa.

**Material examined.** Limassol District: Yermasoyia Dam, 34.755799°N, 33.096194°E, 7.III.2017, Bee Course students leg., M. Schwarz det. (1♀).

**Distribution.** Cyprus, Widespread in Europe, Northern Africa, Western Asia, Central Asia (Kazakhstan, Kyrgyzstan), Southern Asia (Pakistan).

***Nomada fulvicornis* Fabricius, 1793**

**References.**Pittioni (1950); Smit (2018).

**Distribution.** Cyprus, Widespread in Europe, Northern Africa, Western Asia, Central Asia, Southern Asia (Iran, Pakistan, India), Eastern Asia (China).

***Nomada furva* Panzer, 1798**

**References.**Smit (2018).

**Distribution.** Cyprus, Western Europe, Southern Europe, Eastern Europe, Northern Africa (Algeria), Western Asia (Turkey, Georgia), Central Asia.

***Nomada gageae* Schwarz & Smit, 2018**

**Type locality–country.** Cyprus, Limassol: 34.66839°N, 33.03252°E, 25.I.1949 [“1948”], G.A. Mavromoustakis leg., J. Smit det. ♀, (MSAA). Paratypes: Cyprus, Limassol, 28.I.1949 [“1948”], G.A. Mavromoustakis leg., J. Smit/M. Schwarz det. 2♂.

**References.**Smit (2018).

**Distribution. Cyprus. ENDEMIC.**

***Nomada goodeniana* (Kirby, 1802)**

**References.**Smit (2018).

**Distribution.** Cyprus, Widespread in Europe, Western Asia, Central Asia, Southern Asia (Iran).

***Nomada immaculata* Morawitz, 1874**

**References.** Mavromoustakis (1949 [“1948”]); Smit (2018).

**Mavromoustakis localities.** Polemedia Hills, Episkopi, Yerasa.

**Distribution.** Cyprus, Southern Europe (Greece, North Macedonia), Eastern Europe (Hungary, Russia), Northern Africa, Western Asia (Armenia), Southern Asia (Afghanistan, Pakistan).

***Nomada incisa* Schmiedeknecht, 1882**

**References.**Smit (2018).

**Distribution.** Cyprus, Southern Europe, Eastern Europe, Western Asia (Turkey).

***Nomada integra* Brullé, 1832**

**References.**Smit (2018).

**Distribution.** Cyprus, Widespread in Europe, Northern Africa, Western Asia, Central Asia.

***Nomada kohli* Schmiedeknecht, 1882**

**References.**Smit (2018).

**Distribution.** Cyprus, Western Europe (France, Germany, Austria), Southern Europe, Eastern Europe, Northern Africa, Western Asia (Turkey, Israel).

***Nomada kornosica* Mavromoustakis, 1958**

**Type locality–country.** Cyprus, Mt. Kornos (Northern Mountains): 35.1379°N, 33.1379°E, 23.III.1953, G.A. Mavromoustakis leg., G.A. Mavromoustakis det. ♀.

**References.**Mavromoustakis (1958); Smit (2018).

**Distribution.** Cyprus, Southern Europe (Greece).

***Nomada limassolica* Mavromoustakis, 1955**

**Type locality–country.** Cyprus, Limassol: 34.66839°N, 33.03252°E, 13.III.1953, G.A. Mavromoustakis leg., G.A. Mavromoustakis det. ♂, (DAAN).

**References.** Mavomoustakis (1955, 1957); Smit (2018).

**Distribution.** Cyprus, Southern Europe (Greece), Eastern Europe (Bulgaria), Western Asia (Turkey, Israel).

***Nomada lucidula* Schwarz, 1967**

**References.**Smit (2018).

**Distribution.** Cyprus, Southern Europe (Albania, Greece), Eastern Europe (Bulgaria), Western Asia (Turkey, Israel).

***Nomada mutica* Morawitz, 1872**

**References.**Smit (2018).

**Distribution.** Cyprus, Western Europe, Southern Europe, Eastern Europe (Hungary, Romania), Western Asia.

***Nomada nesiotica* Mavromoustakis, 1958**

**Type locality–country.** Cyprus, Fasoula [8 km N of Limassol]: 34.761667°N, 33.026944°E, 9.III.1951, G.A. Mavromoustakis leg., G.A. Mavromoustakis det. ♀, (DAAN).

**References.**Mavromoustakis (1958 ["1957"]); Smit (2018).

**Mavromoustakis localities.** Limassol, Fasoula, Apsiou, Yerasa, Amathus, Yermasoyia River, Pera Pedi, Lania, Trimiklini, Kellaki.

**Material examined.** Limassol District: Yermasoyia Dam, 34.755799°N, 33.096194°E, 7.III.2017, Bee Course students leg., M. Schwarz det. (8♀).

**Distribution.** Cyprus, Southern Europe (Greece).

***Nomada numida* Lepeletier, 1841**

**References.** Mavromoustakis (1949 [“1948”]); Smit (2018).

**Mavromoustakis localities.** Ayios Athanasios, Yermasoyia River, Near Paramytha, Ayia Varvara (Stavrovouni), Pernera coast of Paralimni.

**Distribution.** Cyprus, Western Europe (France), Southern Europe, Eastern Europe, Northern Africa, Western Asia (Turkey, Israel, Iraq).

***Nomada pallispinosa* Schwarz, 1967**

**References.**Smit (2018).

**Material examined.** Limassol District: Polemidia, 34.71178°N, 33.004775°E, 8.III.2017, Bee Course students leg., M. Schwarz det. (2♀).

**Distribution.** Cyprus, Southern Europe, Eastern Europe (Bulgaria, Russian Federation), Western Asia (Turkey, Israel).

***Nomada pleurosticta*** Herrich-**Schäffer, 1839**

**References.**Smit (2018).

**Distribution.** Cyprus, Western Europe, Southern Europe (Spain, Greece), Eastern Europe (Czech Republic, Hungary, Slovakia), Northern Africa (Tunisia), Western Asia (Turkey), Southern Asia (Iran).

***Nomada polemediana* Mavromoustakis, 1957**

**Type locality–country.** Cyprus, Limassol: 34.66839°N, 33.03252°E, 17.IV.1954, G.A. Mavromoustakis leg., G.A. Mavromoustakis det. ♀, (DAAN).

**References.**Mavromoustakis (1957b; Warncke (1967); Smit (2018).

**Distribution. Cyprus. ENDEMIC.**

***Nomada propinqua* Schmiedeknecht, 1882**

**References.**Mavromoustakis (1952); Smit (2018).

**Mavromoustakis localities.** Apsiou, hills near Paramytha, Mt. Kornos.

**Distribution.** Cyprus, Southern Europe, Western Asia (Israel).

***Nomada pyrgosica* Schwarz & Smit, 2018**

**Type locality–country.** Cyprus, Famagusta, Rizokarpaso: 35.59881°N, 34.2772°E, 29.III.2012, Schwenninger leg., J. Smit det. ♀, (MSAA).

**References.**Smit (2018).

**Distribution.** Cyprus, Western Asia (Turkey).

***Nomada stigma* Fabricius, 1804**

**References.**Mavromoustakis (1955, 1957b); Smit (2018).

**Distribution.** Cyprus, Widespread in Europe, Northern Africa (Algeria), Western Asia (Turkey), Central Asia (Kazakhstan).

**Notes.** The subspecies described from Cyprus is *Nomada stigma cypricola* Mavromoustakis, 1955, from Zakaki: 34.6563°N, 33.0029°E, 17.VI.1949, G.A. Mavromoustakis leg. G.A. Mavromoustakis det. ♀, (DAAN).

***Nomada striata* Fabricius, 1793**

**References.**Mavromoustakis (1951, 1957a); Smit (2018).

**Mavromoustakis localities.** Chiflicoudia marshes (near Limassol), Pera Pedi, Amathus.

**Distribution.** Cyprus, Widespread in Europe, Northern Africa (Morocco), Western Asia, Central Asia (Kazakhstan).

***Nomada succincta* Panzer, 1798**

**References.**Scheuchl and Willner (2016); Smit (2018).

**Distribution.** Widespread in Europe, Northern Africa, Western Asia, Central Asia (Turkmenistan, Kazakhstan).

***Nomada teunisseni* Schwarz & Smit, 2018**

**Type locality–country.** Cyprus, Akanthou: 35.3741°N, 33.7558°E, 12.III.1981, H. Teunissen leg., J. Smit det. ♂ (MSAA). Paratypes: Cyprus, Mt Kornos, 9.III.1981, H. Teunissen leg., Smit/ Schwarz det. 1 ♂; Cyprus, Kantara, 2000m, 19.III.1971, K.M. Guichard leg., Smit/ Schwarz det. 1 ♀.

**References.**Smit (2018).

**Distribution. Cyprus. ENDEMIC.**

***Nomada thersites* Schmiedeknecht, 1882**

**References.**Smit (2018).

**Distribution.** Cyprus, Southern Europe (Spain, Italy, Greece), Eastern Europe (Hungary, Ukraine, Russian Federation), Western Asia (Turkey, Israel), Central Asia (Kazakhstan, Kyrgyzstan), Southern Asia (Iran).

***Nomada tridentirostris* Dours, 1873**

**References.**Smit (2018).

**Distribution.** Cyprus, Western Europe (France, Switzerland, Austria), Southern Europe, Eastern Europe (Poland, Hungary, Bulgaria), Northern Africa, Western Asia (Turkey, Israel), Southern Asia (Iran).

***Nomada trispinosa* Schmiedeknecht, 1882**

**References.**Mavromoustakis (1952, 1953); Smit (2018).

**Mavromoustakis localities.** Limassol, Cherkes, Apsiou, Trimiklini.

**Material examined.** Limassol District: Yermasoyia Dam, 34.755799°N, 33.096194°E, 7.III.2017, (6♀); Polemidia, 34.71178°N, 33.004775°E, 8.III.2017, (3♀), all records Bee Course students leg., M. Schwarz det.

**Distribution.** Cyprus, Western Europe (Austria), Southern Europe (Greece, Slovenia), Eastern Europe, Western Asia (Israel, Jordan, Azerbaijan), Eastern Asia (China).

***Nomada yermasoyiae* Schwarz, Smit & Gusenleitner, 2018**

**References.** Schwarz, Smit and Gusenleitner (2018).

**Type localities.** Cyprus, Cherkes: 23.III.1950, ♀, G.A. Mavromoustakis leg., in coll. M. Schwarz. Allotype: Cyprus, Cherkes, 23.III.1950, ♂, G.A. Mavromoustakis leg., in coll. M. Schwarz. Paratypes: Cyprus: Limassol, 28.III.1959, G.A. Mavromoustakis leg., 1 ♀, 2 ♂; Cyprus, Amathus, 7.III.1966, 1 ♂, G.A. Mavromoustakis leg.

**Distribution.** Cyprus, Western Asia (Israel).

**XYLOCOPINI**

**Genus *Xylocopa* LATREILLE, 1802**

4 species.

**Xylocopa (Copoxyla) iris (Christ, 1791)**

**References.**Pittioni (1950); Mavromoustakis (1949 [“1948”], 1952).

**Mavromoustakis localities.** Moni River, Livadin of Cedars (Paphos Forest).

**Material examined.** Paphos District: 15 km SE Paphos, Kouklia, 34.72°N, 32.55°E, 20.VI.2013, (1♂); 20 km N Paphos, Kathikas, 34.90°N, 32.42°E, 20.VI.2013, (1♀, 1♂); 20 km NNW Paphos, Lara Beach, 34.94°N, 32.31°E, 20.VI.2013, (1♀, 1♂); 6 km NE Polis, beach, 35.06°N, 32.46°E, 20.VI.2013, (1♂); N of Elia Bridge, 34.900977°N, 32.776759°E, 29.IV.2015, (1♂); Limassol District: 8 km E Limassol, on road, 34.67°N, 32.85°E, 20.VI.2013, (1♀); Anogyra to Avdimou Road km 2, 34.723986°N, 32.736892°E, 3.V.2015, (1♀); 0.7 km N of Anogyra, 34.745537°N, 32.73385°E, 3.V.2015, (1♀), all 15 km SE Paphos Kouklia, 20 km N Paphos Kathikas, 20 km NNW Paphos Lara Beach, 6 km NE Polis, beach and 8 km E Limassol, on road records C. Schmid-Egger leg./det., all N of Elia Bridge, Anogyra to Avdimou Road km 2 and 0.7 km N of Anogyra records S.P.M. Roberts leg./ det.

**Distribution.** Cyprus, Western Europe (France, Switzerland, Austria), Southern Europe, Eastern Europe, Northern Africa, Western Asia, Central Asia, Southern Asia (Iran, Afghanistan).

**Xylocopa (Proxylocopa) olivieri Lepeletier, 1841**

**References.**Pittioni (1950); Mavromoustakis (1949 [“1948”], 1951, 1952).

**Mavromoustakis localities.** Limassol, Akrotiri Forest, Evdhimou River, Pera Pedi, Platres, Near Kilani, Near Cape Akamas, Near Trimiklini.

**Material examined.** Nicosia District: Kakopetria, 34.992°N, 32.9082°E, 15-17.IX.2011, (1♀), pan trap (UAEG); Kakopetria, 34.992°N, 32.9082°E, 14-16.V.2012, (1♂, 1♀), pan trap (UAEG), all records S. Dimitriou leg., J. Devalez det.

**Distribution.** Cyprus, Southern Europe (Albania, North Macedonia, Greece), Eastern Europe (Russian Federation), Northern Africa (Egypt), Western Asia (Israel), Central Asia (Turkmenistan, Kyrgyzstan), Southern Asia (Iran).

**Xylocopa (Koptortosoma) pubescens Spinola, 1838**

**References.**Rasmont et al. (2017).

**Material examined.** Nicosia District: city centre, 35.1688°N, 33.367°E, 13.III.2006, (1♂), insect net (UAEG); Agios Sozomenos, 35.06687°N, 33.43580°E, 4.III.2019, (1♀) (from photograph); Paphos District: 15 km SE Paphos, Kouklia, 34.72°N, 32.55°E, 20.VI.2013, (1♀); Paphos, Venus Beach Hotel, 34.78°N, 32.40°E, 7.VIII.2016, (1♀) (from photograph); Larnaca District: Prosfygikos Synoikismos EAC, 34.983633°N, 33.745922°E, 2.IV.2016, (1♀); Prosfygikos Synoikismos EAC, 34.983633°N, 33.745922°E, VII.2017, (1♀); Prosfygikos Synoikismos EAC, 34.983633°N 33.745922°E, 2.III.2018, (1♀); Limassol District: Akrotiri, 34.583602°N 32.949536°E, 17.IV.2016, (1♀); Akrotiri, 34.583602°N, 32.949536°E, 8.V.2016 (1♀); Polemidia, 34.71178°N, 33.004775°E, 8.III.2017, (1♀); Akrotiri, 34.600427°N 32.971111°E, 14.IV.2018, (1♀), Nicosia city centre records A. Grace leg., J. Devalez and A. Pauly det., Agios Sozomenos records E. Tzirkalli leg., S.P.M. Roberts det., 15 km SE Paphos, Kouklia records C. Schmid-Egger leg., C. Schmid-Egger det., Paphos, Venus Beach Hotel records S. Bagshaw leg., S.P.M. Roberts det., Prosfygikos Synoikismos EAC (2.IV.2016) A. Varnava leg., S.P.M. Roberts det., Prosfygikos Synoikismos EAC (VII.2017, 2.III.2018) records A. Varnava leg., A. Varnava det., Akrotiri (records 17.IV.2016, 8.V.2016) A. Varnava leg., S.P.M. Roberts det., all Polemidia records S.P.M. Roberts leg./det., Akrotiri records (14.IV.2018) A. Varnava leg./det.

**Distribution.** Cyprus, Southern Europe (Greece), Widespread in Africa, Western Asia, South-eastern Asia (Myanmar), Central Asia, Southern Asia.

**Xylocopa (Xylocopa) violacea (Linnaeus, 1758)**

**References.** Mavromoustakis (1949 [“1948”]); Pittioni (1950); Mavromoustakis (1951, 1952).

**Mavromoustakis localities.** Limassol, Polemedia Hills, Akrotiri Bay, Yerasa, Kitromili near Polemedia, Chiflicoudia marshes, Bogazi shore.

**Material examined.** Nicosia District: Kakopetria, 34.992°N, 32.9082°E, 25-27.IV.2012, (1♀), pan trap (UAEG); Limassol District: Amiantos, 34.918°N, 32.9472°E, 31.V.2012, 2.VI.2012, (1♀), pan trap (UAEG); Paphos District: 20 km NNW Paphos, Lara Beach, 34.94°N, 32.31°E, 20.VI.2013, (4♀), all Nicosia district and Amiantos records S. Dimitriou leg., J. Devalez det., all Paphos district records C. Schmid-Egger leg., C. Schmid-Egger det.

**Distribution.** Cyprus, Widespread in Europe.

## Discussion

The first modern checklist of the wild bees of Cyprus provides information on the species reported on the island based on previous publications and new collections. The list contains 369 species of wild bees that were accepted/verified by the authors to occur on the island. The Mediterranean is well known for its high bee diversity, a result of the high floral diversity and optimal weather conditions (Michener 2007, Nieto et al. 2014).

**Figure 3.**
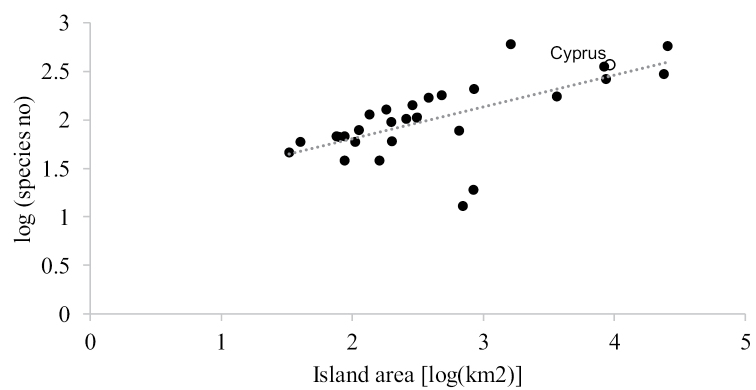
Diversity of bee fauna in 30 Mediterranean islands as a function of island area (logarithm transformed data). See text for the islands and their untransformed bee species numbers. Cyprus represented by the open circle.

Cyprus has greater species richness of bees than larger islands, such as New Guinea, Honshu, Great Britain, and Borneo (Ascher and Pickering 2018). The bee species richness of the island compares well to that of other Mediterranean islands (Fig. 3), with only Lesvos (at least 600 species: Nielsen et al. 2011, Petanidou et al. unpublished data) and Sicily (562 species: [cf. 575 species according to J. S. Ascher, unpublished, based in part on Scheuchl and Willner 2016]) having greater richness than Cyprus. Crete, Sardinia, and Corsica are reported to host 351, 296, and 263 bee species, respectively (Ascher unpublished, cf. Scheuchl and Willner 2016; Ascher and Pickering 2018), while Mallorca 175 (Baldock 2014), Malta 108 (Balzan et al. 2016, 2017), Ibiza and Formentera 77 (Baldock 2014), Madeira and Porto Santo 19 (Fellendorf et al. 1999) and Menorca 13 species (Baldock 2014). A recent study by Kaloveloni et al. (2018) reported the following bee species diversity for islands in the Aegean Archipelago: Anafi (59), Chios (208), Folegandros (46), Ikaria (102), Ios (78), Karpathos (105), Kea (114), Kos (142), Kythnos (59), Limnos (178), Milos (38), Mykonos (38), Paros (60), Samothraki (128), Santorini (67), Serifos (68), Syros (68), Thassos (169), and Tinos (95). However, differences in the sampling effort between islands may bias bee diversity estimates. Sampling for wild bees on the island of Cyprus has focused traditionally on the southern part of the island (Fig. 1). Collections of samples reported for the first time in the current study also concentrated on the southern part of the island (Fig. 1), where the Republic of Cyprus exercises full control, because of difficulties in reaching and collecting samples from the northern part of Cyprus. The under-sampling in the north of the island, both historical and present, probably leads to an underestimation of the bee species richness on the island.

The bee fauna of Cyprus shows similarity to that of southern Europe and Middle East (Levant), while the similarity with North Africa appears weaker. Cyprus meets the definition of an oceanic island as it emerged from the sea, but the Messinian Salinity Crisis that led to the virtual drying up of the Mediterranean around 6 mya (Nicolaou et al. 2016) probably facilitated the arrival of many species of bees, as has been proposed for other species, including mammals (Nicolaou et al. 2016). The more recent drop of the sea level during the last glacial maxima of the Pleistocene (25,000 to 18,000 years ago) that exposed underwater seamounts could also have aided bee dispersal from Asia Minor (Anatolia) in a stepping stone model, even though bees would need to fly over 40 km over water to reach the different islets (Nicolaou et al. 2016). A detailed analysis of the biogeographical affinities of the species present on the island will improve our understanding of historical dispersal events.

There are 2,051 bee species in Europe (Rasmont et al. 2017), as listed in the European Red List of Bees, with 400 species endemic to Europe (20.4 %) (Nieto et al. 2014). Most endemic species occur in southern Europe with a higher diversity in the Mediterranean, predominantly in the Iberian, Italian, and Balkan Peninsulas. Cyprus with 21 endemic species ranks second after Crete with 52 endemics followed by Sicily (10), Corsica (5) and Sardinia (1). The wild bee endemism rate for Cyprus (5.7 %) is similar to that for butterflies (6.1 %), with three endemics out of 49 species (John 2016), but much lower than that for Orthoptera (16.9 %, Siedle et al. 2016). The endemic species are mainly medium- to small-sized bees and mostly ground nesters. They have been reported to forage on many plant species, some of which are endemic to the island, including *Onobrychis venosa* (Fig. 2D), *Teucrium cyprium*, and *Nepeta troodi* (Edwards et al. 2016).

Approximately 9 % of assessed European bee species are classified as threatened [0.4 % – Critically Endangered, 2.4 % – Endangered, 1.2 % – Vulnerable, 5.2 % – Near Threatened, (Nieto et al. 2014)]. In Cyprus, the endemic *Megachile cypricola* is listed as Critically Endangered. Field work conducted in support of the current study has shown that the species is still present in Cyprus, but the population size and trend still need to be evaluated to review its status. As the specimens were collected only on the endemic plant *Onobrychis venosa*, a first mitigation measure could be the conservation of strong populations of the host plant. Moreover, *Ammobatoides abdominalis*, *Bombus niveatus* and *Parammobatodes minutus*, listed as Endangered in Europe (Nieto et al. 2014), require re-evaluation of their conservation status, as they have not been collected from the island in recent years.

Climate change, degradation and fragmentation of natural habitats, urbanisation, monoculture farming and frequent use of pesticides affect the diversity of bees throughout the world (Potts et al. 2010). Cyprus is no exception to the rule, as natural areas are converted into development projects to meet the increasing needs of the tourism and housing industry. The intensive use of pesticides in farm fields is another factor that potentially impacts wild bee populations (Michener 2007), and anecdotal evidence suggests that honeybee poisonings are frequent on the island. The value of the 32 insect-pollinated crops grown on Cyprus exceeds €37 million euros annually (Agricultural Statistics 2015), underlining the importance of taking measures to conserve both honeybee and wild bee populations. While the role of wild bees in pollinating agricultural crops has never been studied before on Cyprus, it is very likely that they support and enhance agricultural production, especially in areas where honeybee populations are low.

Conserving wild bees requires the establishment of a monitoring program to assess the most significant pressures on their populations and to identify effective conservation practices. Climate change and/or other anthropogenic pressures including agricultural intensification and conversion of natural areas to urban fabric are prominent factors that need to be studied, but without more recent data on population trends it is almost impossible to design effective conservation practices. Many of the original locations where Mavromoustakis sampled for bees have been or are being converted into urban areas. Future studies need to document the impact of urban development on bee conservation and the potential value of anthropogenic habitats for wild bee conservation. The current work provides a baseline for future studies of wild bee diversity on the island of Cyprus and elsewhere.

## Conclusions

Mediterranean islands are well known for their bee diversity, a result of their isolation, high floral diversity and optimal nesting conditions (Nieto et al. 2014, Kaloveloni et al. 2018). Cyprus hosts 369 bee species, with 5.7 % endemic to the island. The island hosts species of conservation concern in Europe, such as the Critically Endangered *Megachile cypricola* and the Endangered *Ammobatoides abdominalis*, *Bombus niveatus*, and *Parammobatodes minutus*. Conserving wild bees requires the establishment of a monitoring program to assess their population trends, the most significant threats on their populations and to identify effective conservation practices. The current study is the first step towards conserving wild bees on the island of Cyprus.

## Supplementary materials

Supplementary material 1 Species list Data type species data Explanation note The list below is of species reported from Cyprus, but which are of uncertain occurrence on the island or have been excluded recently. https://binary.pensoft.net/file/397320This dataset is made available under the Open Database License (http://opendatacommons.org/licenses/odbl/1.0/). The Open Database License (ODbL) is a license agreement intended to allow users to freely share, modify, and use this Dataset while maintaining this same freedom for others, provided that the original source and author(s) are credited.Androulla I. Varnava, Stuart P.M. Roberts, Denis Michez, John S. Ascher, Theodora Petanidou, Stavroula Dimitriou, Jelle Devalez, Marilena Pittara, Menelaos C. Stavrinides

## References

[B1] Agricultural Statistics (2015) Cyprus Statistical Service. Nicosia, Cyprus, 111 pp.

[B2] Alfken JD (1912) Die Bienenfauna von Westpreussen. Bericht des Westpreussischen Botanisch-Zoologischen Vereins 34: 1–96.

[B3] Alfken JD (1928) Zur Kenntnis der *Prosopis*-Arten von Cypern. Konowia (Vienna) 7: 56–61. 10.1002/mmnd.48119280106

[B4] Alfken JD (1933) Beiträge zur kenntnis paläarktischer Bienen. (Hym. Apid.) 2. Beitrag Deutsche Entomologische Zeitschrift 1933: 64–71. 10.1002/mmnd.193319330105

[B5] Alfken JD (1940) Neue *Coelioxys*-Arten von Cyp (Hym. Apid.). Mitteilungen der Münchner Entomologischen Gesellschaft 30: 1058–1059.

[B6] Ascher JS (2016) Collaborative databasing of North American bee collections within a global informatics network project. iDigBio Darwin Core Archive Recordset. https://www.idigbio.org/portal/recordsets/8919571f-205a-4aed-b9f2-96ccd0108e4c

[B7] Ascher JS Pickering J (2018) Discover Life bee species guide and world checklist (Hymenoptera: Apoidea: Anthophila). http://www.discoverlife.org/mp/20q?guide=Apoidea_species&flags=HAS [Draft 51, 1 November 2018]

[B8] Baldock D (2014) A provisional list of the wasps and bees of Mallorca, Balearic Islands, Spain (Hymenoptera, Aculeata: Chrysidoidea, Scolioidea, Vespoidea, Apoidea). Entomofauna Zeitschrift Fur Entomologie 35: 333–404.

[B9] Balzan MV Rasmont P Kuhlmann M Dathe HH Pauly A Patiny S Terzo M Michez D. (2016) The bees (Hymenoptera: Apoidea) of the Maltese Islands. Zootaxa 4162: 225–244. 10.11646/zootaxa.4162.2.2 27615971

[B10] Balzan M Genoud D Rasmont P Schwarz M Michez D (2017) New records of bees (Hymenoptera: Apoidea) from the Maltese Islands. Journal of Melittology 72: 1–9. 10.17161/jom.v0i72.6626

[B11] Blüthgen P (1923) Beiträge zur systematik der Bienen-gattung *Halictus* Latr. (Hym.). I. Die Binden-*Halictus* (Gruppe des *sexcinctus* F.). Konowia: Zeitschrift für Systematische Insektenkunde 2: 123–142.

[B12] Blüthgen P (1937) Neue Halictini aus Cypern (Hym., Apidae, Halictinae). Konowia: Zeitschrift für Systematische Insektenkunde 16: 41–54.

[B13] Bogusch P Hadrava J (2018) European bees of the genera *Epeolus* Latreille, 1802 and *Triepeolus* Robertson, 1901 (Hymenoptera: Apidae: Nomadinae: Epeolini): taxonomy, identification key, distribution, and ecology. Zootaxa 4437: 1–60. 10.11646/zootaxa.4437.1.1 30313168

[B14] Charalambidou I Sparrow D Stapley J Richardson C (2016) Aves. In: Sparrow JD John E (Eds) An Introduction to the Wildlife of Cyprus.Terra Cypria, Limassol, 695–771.

[B15] Christofides Y (2017) Illustrated Flora of Cyprus. Multiprint Ltd. Kostinbrod, 383 pp.

[B16] Cockerell TDA (1910) New and little-known bees. Transactions of the American Entomological Society 36: 199–249.

[B17] Cockerell TDA (1914) Descriptions and records of bees. Annals and Magazine of Natural History 8: 136–146. 10.1080/00222931408693461

[B18] Cockerell TDA (1931) Descriptions and records of bees. Annals and Magazine of Natural History 10: 273–280. 10.1080/00222933108673310

[B19] Delipetrou P Makhzoumi J Dimopoulos P Georghiou K (2008) Cyprus. In: Vogiatzakis IN Pugnetti G Mannion AM (Eds) Mediterranean Island Landscapes.Springer, Netherlands, 170–203. 10.1007/978-1-4020-5064-0_9

[B20] Dorchin A Praz CJ (2018) Taxonomic revision of the Western Palaearctic bees of the subgenus Pseudomegachile (Hymenoptera, Apiformes, Megachilidae, *Megachile*). Zootaxa 4524: 251–307. 10.11646/zootaxa.4524.3.1 30486110

[B21] Dorchin A Lopez-Uribe MM Praz CJ Griswold T Danforth BN (2018) Phylogeny, new generic-level classification, and historical biogeography of the *Eucera* complex (Hymenoptera: Apidae). Molecular Phylogenetics and Evolution 119: 81–92. 10.1016/j.ympev.2017.10.007 29122650

[B22] Dours JA (1870) Monographie iconographique du genre *Anthophora*, Lat. Mémoires de la Société Linnéenne du Nord de la France, 322 pp. 10.5962/bhl.title.9337

[B23] Ebmer AW (1975) Neue Westpaläarktische Halictidae (Halictinae, Apoidea). Teil III. Linzer Biologische Beiträge 7: 41–118.

[B24] Ebmer AW (1981) *Halictus* und *Lasioglossum* aus Kreta (Halictidae, Apoidea). Linzer Biologische Beiträge 13: 101–127.

[B25] Ebmer AW (1988) Kritische Liste der nicht-parasitischen Halictidae Österreichs mit Berucksichtigung aller mitteleuropaïschen Arten (Insecta: Hymenoptera: Apoidea: Halictidae). Linzer Biologische Beiträge 20: 527–711.

[B26] Ebmer AW (2014) Die nicht-parasitischen Halictidae der Insel Zypern in Vergleich zu Kreta mit einer Monographie der *Lasioglossum bimaculatum*-Artengruppe und einer Ubersicht der *Halictus nicosiae*-Untergruppe (Insecta: Hymenoptera: Apoidea: Halictidae). Linzer Biologische Beiträge 46: 291–413.

[B27] Edwards M Varnava A Stavrinides M John E (2016) Hymenoptera. Bees, Wasps and Ants. In: Sparrow D John E (Eds) An Introduction to the Wildlife of Cyprus.Terra Cypria, Limassol, 411–441.

[B28] Erlandsson S (1979) HymenopteraAculeata from the European part of the Mediterranean countries, II. Acta Entomologica Jugoslavica 15: 111–130.

[B29] Erlandsson S (1986) HymenopteraAculeata from the European parts of the Mediterranean countries, III. Bollettino del Museo Civico di Storia Naturale di Venezia 35: 53–66.

[B30] Evenhuis NL (2003) Publication and dating of the journals forming the Annals and Magazine of Natural History and the Journal of Natural History. Zootaxa 385: 1–68. 10.11646/zootaxa.385.1.1

[B31] Fauna Europaea (2012) Fauna Europaea. https://fauna-eu.org/ [Accessed on 2012-1-6]

[B32] Fellendorf F Mohra C Roberts S Wirtz Van Der Zanden G (1999) The bees of Madeira (Hymenoptera, Apoidea). Bocagiana 197: 1–17.

[B33] Georghiou GP (1977) The Insects and Mites of Cyprus. Benaki Phytopathological Institute, Athens, 347 pp.

[B34] Grace A (2010) Introductory Biogeography to Bees of the East Mediterranean and Near East. Bexhill Museum, Sussex, 284 pp.

[B35] Gusenleitner F Schwarz M (2002) Weltweite Checkliste der Bienengattung *Andrena* mit Bemerkungen und Ergänzungen zu paläarktischen Arten (Hymenoptera, Apidae, Andreninae, Andrena). Entomofauna, Supplement 12: 1–280.

[B36] IUCN (2019) The IUCN Red List of Threatened Species. Version 2019-2. http://www.iucnredlist.org [Downloaded on 18 July 2019]

[B37] John E Skule B (2016) Butterflies and Moths. In: Sparrow DJ John E (Eds) An Introduction to the Wildlife of Cyprus.Terra Cypria, Limassol, 269–385.

[B38] Kaloveloni A Tscheulin T Petanidou T (2018) Geography, climate, ecology: What is more important in determining bee diversity in the Aegean Archipelago? Journal of Biogeography 45: 2690–2700. 10.1111/jbi.13436

[B39] Kasparek M (2015) The cuckoo bees of the genus *Stelis* Panzer, 1806 in Europe, North Africa and the Middle East. A review and identification guide. Entomofauna 18: 1–144.

[B40] Kuhlmann M Ascher JS Dathe HH Ebmer AW Hartmann P Michez D Müller A Patiny S Pauly A Praz C Rasmont P Risch S Scheuchl E Schwarz M Terzo M Williams PH Amiet F Baldock D Berg Ø Bogusch P Calabuig I Cederberg B Gogala A Gusenleitner F Josan Z Madsen H.B Nilsson A Ødegaard F Ortiz-Sanchez J Paukkunen J Pawlikowski T Quaranta M Roberts SPM Sáropataki M Schwenninger HR Smit J Söderman G Tomozei B (2015) Checklist of the West Palaearctic Bees (Hymenoptera: Apoidea: Anthophila). http://westpalbees.myspecies.info [Accessed on: 2018-4-6]

[B41] Lieftinck MA (1968) A review of Old World Species of *Thyreus* Panzer (=*Crocisa* Jurine) (Hym., Apoidea, Anthophoridae) Part 4. Palearctic Species. Zoologische Verhandelingen 98: 1–139.

[B42] Lieftinck MA (1980) Prodrome to a monograph of the Palaearctic species of the genus *Melecta* Latreille 1802 (Hymenoptera, Anthophoridae). Tijdschriftvoor Entomologie 123: 129–349.

[B43] Lieftinck MA (1983) Notes on the nomenclature and synonymy of Old World melectine and anthophorine bees (Hymenoptera, Anthophoridae). Tijdschrift voor Entomologie 126: 269–284.

[B44] Mavromoustakis GA (1937a) Some new Asiatic bees of the subfamily Anthidiinae (Apoidea). Annals and Magazine of Natural History 10(19): 151–157. 10.1080/00222933708655248

[B45] Mavromoustakis GA (1937b) Three new species of bees of the genus *Osmia* (Apoidea) from Cyprus. Annals and Magazine of Natural History 10(20): 520–525. 10.1080/00222933708655375

[B46] Mavromoustakis GA (1938) New bees of the genera *Osmia* and *Megachile* from Cyprus. Annals and Magazine of Natural History 11(2): 464–473. 10.1080/00222933808526875

[B47] Mavromoustakis GA (1939a) New and little-known bees of the subfamily Anthidiinae (Apoidea). Part I. Annals and Magazine of Natural History (11)3: 88–97. 10.1080/00222933908526902

[B48] Mavromoustakis GA (1939b) On the bees of the genera *Osmia* and *Megachile* from Cyprus (Apoidea). I. Annals and Magazine of Natural History (11)3: 154–160. 10.1080/00222933908526979

[B49] Mavromoustakis GA (1949 [“1948”]) On the bees (Hymenoptera: Apoidea) of Cyprus. Part I. Annals and Magazine of Natural History (12)1: 541–587. 10.1080/00222934808653931

[B50] Mavromoustakis GA (1951) On the bees (Hymenoptera: Apoidea) of Cyprus. Part II. Annals and Magazine of Natural History (12)4: 334–354. 10.1080/00222935108654159

[B51] Mavromoustakis GA (1952) On the bees (Hymenoptera: Apoidea) of Cyprus. Part III. Annals and Magazine of Natural History (12)5: 814–843. 10.1080/00222935208654357

[B52] Mavromoustakis GA (1953) On the bees (Hymenoptera: Apoidea) of Cyprus. Part IV. Annals and Magazine of Natural History (12)6: 769–781. 10.1080/00222935308654482

[B53] Mavromoustakis GA (1954) On the bees (Hymenoptera, Apoidea) of Cyprus. Part V. Annals and Magazine of Natural History (12)7: 578–588. 10.1080/00222935408651756

[B54] Mavromoustakis GA (1955) On the bees (Hymenoptera: Apoidea) of Cyprus. Part VI. Annals and Magazine of Natural History (12)8: 97–105. 10.1080/00222935508651833

[B55] Mavromoustakis GA (1956) On some bees of the genus *Andrena* from the Islands Crete and Cyprus (Hymenoptera: Apoidea). Beiträge zur Entomologie 6: 580–589. 10.21248/contrib.entomol.6.5-6.580-589

[B56] Mavromoustakis GA (1957a) The bees (Hymenoptera: Apoidea) of Cyprus. Part VII. Annals and Magazine of Natural History (12)10: 321–337. 10.1080/00222935708655964

[B57] Mavromoustakis GA (1957b) New bees of the genera *Andrena* and *Nomada* from the island Cyprus (Hymenoptera: Apoidea). Beiträge zur Entomologie 7: 42–49.

[B58] Mavromoustakis GA (1958 [“1957”]) On the bees (Hymenoptera: Apoidea) of Cyprus. Part VIII. Annals and Magazine of Natural History (12)10: 843–850. 10.1080/00222935708656085

[B59] Mavromoustakis GA (1958) New bees of the genera *Andrena* and *Nomada* from the island Cyprus (Hymenoptera: Apoidea) Part II. Beiträge zur Entomologie 8: 212–219.

[B60] Michener CD (2007) The Bees of the World (2^nd^ edn). The Johns Hopkins University Press, Baltimore, Maryland, 992 pp.

[B61] Michez D Terzo M Rasmont P (2004) Révision des espèces ouest-paléarctiques du genre *Dasypoda* Latreille 1802 (Hymenoptera, Apoidea, Melittidae). Linzer Biologische Beiträge 36: 847–900.

[B62] Müller A (2012) New European bee species of the tribe Osmiini (Hymenoptera: Apoidea: Megachilidae). Zootaxa 3355: 29–50. 10.11646/zootaxa.3355.1.2 37044781

[B63] Müller A (2018) Palaearctic Osmiine Bees, ETH Zürich. http://blogs.ethz.ch/osmiini [Accessed on 2019-22-10]

[B64] Myers N Mittermeier RA Mittermeier CG Fonseca GAB. da Kent J (2000) Biodiversity hotspots for conservation priorities. Nature 43: 853–858. 10.1038/35002501 10706275

[B65] Nieto A Roberts SPM Kemp J Rasmont P Kuhlmann M García Criado M Biesmeijer JC Bogusch P Dathe HH De la Rúa P De Meulemeester T Dehon M Dewulf A Ortiz Sánchez FJ Lhomme P Pauly A Potts SG Praz C Quaranta M Radchenko VG Scheuchl E Smit J Straka J Terzo M Tomozii B Window J Michez D (2014) European Red List of Bees. Publication Office of the European Union, Luxembourg, 84 pp.

[B66] Nicolaou H Hadjisterkotis E Sparrow JD Sparrow R (2016) Mammalia. In: Sparrow, J.D. and John, E. (Eds), An Introduction to the Wildlife of Cyprus. Terra Cypria, Limassol, 773–834.

[B67] Nielsen A Steffan-Dewenter I Westphal C Messinger O Potts S Roberts SM Settele J Szentgyörgyi H Vaissière B Vaitis M Woyciechowski M Bazos I Biesmeijer J Bommarco R Kunin W Tscheulin T Lamborn E Petanidou T (2011) Assessing bee species richness in two Mediterranean communities: importance of habitat type and sampling techniques. Ecological Research, 26: 969–983. 10.1007/s11284-011-0852-1

[B68] Noskiewicz J (1936) Die palaearktischen *Colletes*-Arten. Prace Naukowe Wydawnictwo Towarzystwa Naukowego we Lwowie 3: 1–532.

[B69] Pauly A Devalez J Sonet G Nagy TZ Boeve JL (2015) DNA barcoding and male genital morphology reveal five new cryptic species in the West Palearctic bee *Seladonia smaragdula* (Vachal, 1895) (Hymenoptera: Apoidea: Halictidae). Zootaxa 4034: 257–290. 10.11646/zootaxa.4034.2.2 26624441

[B70] Pauly A Noël G Sonet G Notton DG Boevé J-L (2019) Integrative taxonomy resuscitates two species in the *Lasioglossum villosulum* complex (Kirby, 1802) (Hymenoptera: Apoidea: Halictidae). European Journal of Taxonomy 541: 1–43. 10.5852/ejt.2019.541

[B71] Pesenko YA (2005) New data on the taxonomy and distribution of the Palaearctic halictids: genus *Halictus* Latreille (Hymenoptera: Halictidae). Entomofauna 26: 313–348.

[B72] Pittioni B (1950) HymenopteraAculeata I. On the insect fauna of Cyprus. Results of the Expedition of 1939 by Harald, Hakan and P. H. Lindberg. Commentationes Biologicae, Societas Scientarum Fennica 10: 1–94.

[B73] Popov VB (1944) Some parasitic bees from Cyprus (Hymenoptera, Apoidea). Proceedings of the Royal Entomological Society of London 13: 120–124. 10.1111/j.1365-3113.1944.tb00801.x

[B74] Potts SG Biesmeijer JC Kremen C Neumann P Schweiger O Kunin WE (2010) Global pollinator declines: trends, impacts and drivers. Trends in Ecology & Evolution 25: 345–353. 10.1016/j.tree.2010.01.007 20188434

[B75] Praz CJ (2017) Subgeneric classification and biology of the leafcutter and dauber bees (genus *Megachile* Latreille) of the western Palearctic (Hymenoptera, Apoidea, Megachilidae). Journal of Hymenoptera Research 55: 1–54. 10.3897/jhr.55.11255

[B76] Rasmont P Iserbyt I (2010-2014) Atlas of the European Bees: genus *Bombus* (3^rd^ edn). STEP Project, Atlas Hymenoptera, Mons, Gembloux. http://www.atlashymenoptera.net/page.asp?ID=169

[B77] Rasmont P Franzén M Lecocq T Harpke A Roberts SPM Biesmeijer K Castro L Cederberg B Dvorák L Fitzpatrick Ú Gonseth Y Haubruge E Mahé G Manino A Michez D Neumayer J Ødegaard F Paukkunen J Pawlikowski T Potts S.G Reemer M Settele J Straka J Schweiger O (2015) Climatic risk and distribution atlas of European bumblebees. Biorisk 10: 1–236. 10.3897/biorisk.10.4749

[B78] Rasmont P Devalez J Pauly A Michez D Radchenko VG (2017) Addition to the checklist of IUCN European wild bees (Hymenoptera: Apoidea). Annales de la Société Entomologique de France 53: 17–32. 10.1080/00379271.2017.1307696

[B79] Scheuchl E Willner W (2016) Taschenlexikon der Wildbienen Mitteleuropas – Alle Arten im Porträt. Quelle and Meyer Verlag, Wiebelsheim, 917 pp.

[B80] Schönitzer K Grünwaldt W Gusenleitner F Osytshnjuk AZ Schuberth J (1995) Klärung von *Andrena forsterella*, mit Hinweisen zu den anderen Arten der *Andrena labialis*-Gruppe (Hymenoptera, Apoidea, Andrenidae). Linzer Biologische Beiträge 27: 823–850.

[B81] Schuberth J (1995) Eine als neu erkannte Sandbienenart aus Südosteuropa: *Andrena wilhelmi* n. sp. (Hymenoptera, Apoidea, Andrenidae). Linzer Biologische Beiträge 27: 807–821.

[B82] Schwarz M (1999) Beiträge zur Kenntnis parasitärer Bienen (Hymenoptera: Apoidea). Entomofauna 20: 257–261.

[B83] Schwarz M Smit J Gusenleitner F (2018) Zur Kenntnis paläarktischer Bienen der Gattung *Nomada* Scopoli, 1770 (Hymenoptera, Apidae). Linzer Biologische Beiträge 50: 1403–1445.

[B84] Schwenninger HR (2015) Revision of the West Palaearctic species of the *Andrena taraxaci*-group with description of four new species (Hymenoptera: Andrenidae). Stuttgarter Beiträge zur Naturkunde A 8: 251–270.

[B85] Siedle K Tumbrinck J Tzirkalli E (2016) Grasshoppers, locusts and crickets. In: Sparrow DJ John E (Eds) An Introduction to the Wildlife of Cyprus.Terra Cypria, Limassol, 133–173.

[B86] Smit J (2018) Identification key to the European species of the bee genus *Nomada* Scopoli, 1770 (Hymenoptera: Apidae), including 23 new species. Entomofauna, M 3: 1–253.

[B87] Soltani G Bénon D Alvarez N Praz C (2017) When different contact zones tell different stories: putative ring species in the *Megachile concinna* complex (Hymenoptera, Megachilidae). Biological Journal of the Linnean Society 4: 815–832. 10.1093/biolinnean/blx023

[B88] Sparrow DJ Baier F (2016a) Squamata and Testudines, snakes, lizards, terrapins and turtles. In: Sparrow DJ John E (Eds) An Introduction to the Wildlife of Cyprus.Terra Cypria, Limassol, 645–693.

[B89] Sparrow DJ Baier F (2016b) Amphibians. In: Sparrow DJ John E (Eds) An Introduction to the Wildlife of Cyprus.Terra Cypria, Limassol, 633–643.

[B90] Sparrow DJ Sparrow R De Knijf G (2016) Dragonflies. In: Sparrow DJ John E (Eds) An Introduction to the Wildlife of Cyprus.Terra Cypria, Limassol, 65–131.

[B91] Terzo M (1998) Annotated list of the species of the genus *Ceratina* (Latreille) occurring in the Near East, with description of new species (Hymenoptera: Apoidea: Xylocopinae). Linzer Biologische Beitrage 2: 719–743.

[B92] Tkalců B (1979) Revision der EuropaischenVertreter der Artengruppe von *Tetralonia ruficornis* (Fabricius) (Hymenoptera, Apoidea). Acta Musei Moraviae, Scientiae Naturales, 64: 127–152. [2 pls]

[B93] Tkalců B (1984) Systematisches Verzeichnis der westpaläarktischen *Tetralonia* und *Eucera* Arten, deren Männchen als Blütenbesucher ver. schiedener *Ophrys*-Arten festgestellt wurden. Mit Beschreibung neuer Taxa (Hymenoptera: Apoidea). Nova Acta Regiae Societatis Scientiarum Upsaliensis 3: 57–77.

[B94] UN, Department of Economic and Social Affairs, Population Division (2017) World Population Ageing 2017 (ST/ESA/SER.A/408). https://www.un.org/en/development/desa/population/theme/ageing/WPA2017.asp

[B95] Ungricht S Müller A Dorn S (2008) A taxonomic catalogue of the Palaearctic bees of the tribe Osmiini (Hymenoptera: Apoidea: Megachilidae). Zootaxa 1865: 1–253. 10.11646/zootaxa.1865.1.1

[B96] Varnava AI Stavrinides MC (2015) Wild bees of Cyprus. http://www.wildbeesofcyprus.org/index.html [Accessed on: 2018-6-13]

[B97] Vogiatzakis I Manolaki P Zomi M Zotos S (2016) Habitats of Cyprus. In: Sparrow JD John E (Eds) An Introduction to the Wildlife of Cyprus.Terra Cypria, Limassol, 25–39.

[B98] Wagstaffe A (2016) Geology of Cyprus. In: Sparrow JD John E (Eds) An Introduction to the Wildlife of Cyprus.Terra Cypria, Limassol, 13–23.

[B99] Warncke K (1965) Beitrag zur kenntnis der Bienengattung *Andrena* Fabricius in Griechenland. Beitrage zur Entomologie 15: 27–76. 10.21248/contrib.entomol.15.1-2.27-76

[B100] Warncke K (1967) Beitrag zur Klärung paläarktischer *Andrena*-Arten (Hym., Apidae). Revista Española de Entomología 43(1–2): 171–318.

[B101] Warncke K (1969) A contribution to the knowledge of the genus *Andrena* (Apoidea) in Israel. Israel Journal of Entomology 4: 377–408.

[B102] Warncke K (1972a) Beitrag zur Systematik und Verbreitung der Bienengattung *Prosopis* F. in der Westpaläarktis (Hymenoptera, Apoidea, Colletidae). Bulletin des Recherches Agronomiques de Gembloux 5: 746–768.

[B103] Warncke K (1972) Westpaläarktische Bienen der Unterfamilie Panurginae (Hym., Apidae) [Pszczoly z podrodziny Panurginae (Hym., Apidae) w zachodniej Palearktyce]. Polskie Pismo Entomologiczne 62: 53–108.

[B104] Warncke K (1974) Die Sandbienen der Türkei (Hymenoptera, Apoidea, *Andrena*), Teil A. Mitteilungen der Münchner Entomologischen Gesellschaft 64: 81–116.

[B105] Warncke K (1975) Die Sandbienen der Türkei (Hymenoptera, Apoidea, *Andrena*), Teil B. Mitteilungen der Münchner Entomologischen Gesellschaft 65: 29–102.

[B106] Warncke K (1982) Beitrag zur Bienenfauna des Iran. - 14. Die Gattung *Halictus* Latr., mit Bemerkungen über bekannte und neue *Halictus*-Arten in der Westpaläarktis und Zentralasien.– Bollettino del Museo Civico di Storia Naturale di Venezia 32: 67–166.

[B107] Warncke K (1983) Zur kenntnis der bienengattung *Pasites* Jurine, 1807, in der Westpaläarktis (Hymenoptera, Apidae, Nomadinae). Entomofauna 4: 261–347.

[B108] Warncke K (1992) Die westpaläarktischen Arten der Bienengattung *Sphecodes* Latr. (Hymenoptera, Apidea, Halictinae). Bericht der Naturforschenden Gesellschaft Augsburg 52: 9–64.

[B109] Zanden E (1992) Neue oder unvollständig bekannte arten Paläarktischer Bauchsammler HymenopteraAculeata, Apoidea, Megachilidae). Linzer Biologische Beitrage 24: 65–74.

