# Supplementary material for: The wild bees (Hymenoptera, Apoidea) of the island of Cyprus
Source: Zookeys. 2020 Apr 6;924:1–114. doi: 10.3897/zookeys.924.38328 (PMC7154044; doi:10.3897/zookeys.924.38328)
Supplement: Supplementary material 1 — Species list [file zookeys-924-001-s001.docx]

**Supplementary Material**

The list below is of species reported from Cyprus, but which are of uncertain occurrence on the island or have been excluded recently. The list:

• includes species whose taxonomic status is unclear, often due to incomplete understanding of species boundaries, identification criteria, and distributions of cryptic species (e.g., Pauly et al., 2015; 2019).

• includes species whose presence is based on records not corroborated by further evidence from collections or the most current and reliable literature.

• reports species reports of taxa that are, as currently delimited, not expected to occur in the eastern Mediterranean region.

• reports a morphospecies that may be new to science.

The intent of this section is to document nominal taxa that might potentially occur on Cyprus but cannot be included on the main list without further verification and those that have been plausibly reported by recent authors but are now regarded as pertaining to different, related forms. We do not list all trivial nomenclatural or taxonomic updates nor all clearly erroneous citations.

Georghiou’s checklist Georghiou (1977) recorded a number of nominal taxa from Cyprus, in addition to those currently accepted for the island. Several names cited are mis-cited and cannot be reliably interpreted (e.g., “*Eucera simillima* Sm.” and “*Megachile konowi* Fr.”) and several species that can be interpreted unambiguously are not known in the vicinity of Cyprus (cf. Nieto et al., 2014; Scheuchl and Willner, 2016; Ascher and Pickering, 2018). Taxa cited by Georghiou that we reject as plausibly occurring on Cyprus including the following: *Anthophora garrula* Rossi [= *Amegilla garrula* (Rossi, 1790)], *Andrena florea* Fabricius, 1793, *Anthophora* *pubescens* (Fabricius, 1781), *Tetralonia radozkowsii* Morawitz [=*Eucera alborufa* (Radoszkowski, 1871)], *Halictus* *rubicundus* (Christ, 1791), *Halictus* *sexcinctus* (Fabricius, 1775), and *Halictus tumulorum* (Linnaeus, 1758).

***Andrena* (*Ulandrena*) *abbreviata*** Dours, 1873

*Andrena abbreviata* *sensu stricto* does not occur on Cyprus, whereas the related taxon *polemediana*, now considered a separate species, occurs on Cyprus.

References: Pittioni (1950).

Distribution: Southern Europe (Greece), Eastern Europe.

***Andrena* (*Parandrenella*) *bicarinata*** Morawitz, 1876

Gusenleitner and Schwarz (2002) revised the species and documented its history of misinterpretations. As presently delimited, this is a species of Central Asia and does not occur on Cyprus.

References: Mavromoustakis (1957).

Mavromoustakis localities: Limassol, Cherkes, Pissouri.

Distribution: Central Asia (Tajikistan, Turkmenistan).

***Andrena* (*Chlorandrena*) *taraxaci*** Giraud, 1861

Cited for Cyprus by Scheuchl and Willner (2016) but Schwenninger (2015) recorded only the related species *A. orientana* and *A. crepidis* from Cyprus not *A. taraxaci sensu stricto*.

***Bombus* (*Megabombus*) *argillaceus*** (Scopoli, 1763)

Atlas Hymenoptera <http://www.atlashymenoptera.net/page.asp?id=169> maps do not include Cyprus records for this species, but the text of the species page states, ‘‘*B. argillaceus* is found throughout the Balkanic [sic] Peninsula, in Creta [sic], Cyprus, Turkey, Caucasus, Transcaucasia and N. Iran, including the Kopet-Dag.’’

***Ceratina* (*Euceratina*) *nigrolabiata*** Friese, 1896

M. Terzo (in litt.) comments that all records attributable to “*C. nigrolabiata*” in Cyprus correctly refer instead to an undescribed species of *Ceratina* (*Euceratina*).

References: Mavromoustakis (1949 [“1948”]); Pittioni (1950).

Mavromoustakis localities: Prodromos, Mandria, Evdhimou River.

Distribution: Western Europe (France, Switzerland, Austria), Southern Europe, Eastern Europe, Western Asia (Turkey).

***Colletes hederae*** Schmidt and Westrich, 1993

Recorded from Cyprus on an online species page, but this apparently pertains to *Colletes brevigena* (<https://www.bwars.com/bee/colletidae/colletes-hederae>). Species in the complex are difficult to distinguish, even with reference to DNA barcodes, so further research is required (M. Kuhlmann, in litt.).

***Eucera* (*Eucera*) *nigrescens*** Pérez,1879

Reports of this species pertain to *E. cypria.*

References: Scheuchl and Willner (2016).

Distribution: Cyprus, Western Europe, Southern Europe, Eastern Europe, Western Asia (Turkey, Jordan), Southern Asia (Iran).

***Halictus* (*Halictus*) *fulvipes*** (Klug, 1817)

No firm evidence beyond this single report of Pittioni to suggest this West Mediterranean species is present in Cyprus, and the presence there was explicitly rejected by Scheuchl and Willner (2016) and Ebmer (2014).

References: Pittioni (1950).

Distribution: Western Europe (France), Southern Europe, Eastern Europe (Hungary).

***Halictus* (*Vestitohalictus*) *pici*** Perez, 1895

Reported from Cyprus by Pittioni but not subsequently confirmed (Ebmer, 2014).

References: Pittioni (1950).

Distribution: Northern Africa, Western Asia (Israel).

***Halictus* (*Halictus*) *senilis*** (Eversmann, 1852)

The record pertains to *H. subsenilis* (Ebmer 2014; Kuhlmann et al. 2015).

References: Mavromoustakis (1949 [“1948”]); Pittioni (1950); Mavromoustakis (1957a).

Mavromoustakis localities: Ayia Phyla, Famagusta, Amathus, Episkopi Forest, Ayia Varvara (Stavrovouni), Pernera coast (Paralimni), Near Nicosia.

Distribution: Cyprus, Southern Europe (Spain (Canary Islands)), Eastern Europe (Russian Federation), Northern Africa (Tunisia, Egypt), Western Asia (Israel, Iraq), Central Asia, Southern Asia (Pakistan), Eastern Asia (China, Mongolia).

***Halictus* (*Vestitohalictus*) *vestitus*** Lepeletier, 1841

*Halictus vestitus* has been applied historically to several species of subgenus *Vestitohalictus sensu lato*, with material from Cyprus likely referable to misidentified *H. pollinosus*, *H. cypricus* or *H. pulvereus*.

References: Mavromoustakis (1949 [“1948”]); Pittioni (1950); Georghiou (1977).

Mavromoustakis localities: Limassol.

Distribution: Iberian Peninsula and the Balearic Islands.

***Hylaeus* (*Dentigera*) *brevicornis*** Nylander, 1852

Unverified from Cyprus. The *brevicornis*-group within the subgenus *Dentigera* contains many near cryptic species, current knowledge suggests that historic records are misinterpretations of species like *Hylaeus imparilis*, *Hylaeus kahri* and *Hylaeus intermedius*.

References: Alfken (1928); Mavromoustakis (1949 [“1948”]).

Mavromoustakis localities: Limassol, Mesayitonia, Cherkes, Zakaki, Asomatos, Pernera coast of Paralimni.

Distribution: Cyprus, Widespread in Europe, Northern Africa (Morocco), Southern Asia (Iran).

***Hylaeus* (*Paraprosopis*) *pictipes*** Nylander, 1852

References: Warncke (1972a).

Distribution: Cyprus, Southern Europe, Western Asia (Turkey, Israel).

***Hylaeus* (*Spatulariella*) *punctatus*** (Brullé, 1832)

A very abundant and variable species distributed widely across the Mediterranean Region. In the Eastern Mediterranean its distribution overlaps with later-described sibling species. The historic records very likely refer to other species within the subgenus *Spatulariella* that have been described during the 20^th^ century, like *Hylaeus cypricola* or *Hylaeus longimaculus*.

References: Alfken (1928); Mavromoustakis (1949 [“1948”]).

Mavromoustakis localities: Cherkes, Yermasoyia, Finikaria River, Mesapotamos.

Distribution: Cyprus, Widespread in Europe. Introduced to North and South America.

***Hylaeus* (*Prosopis*) *purpurissatus*** (Vachal, 1895)

There is no reliable evidence to support the presence of this west-Mediterranean taxon in Cyprus. Confirming its presence as a Cyprus species would require DNA work and a major revision of the *Hylaeus gibbus* group.

References: Pittioni (1950).

Distribution: North Africa (Algeria), with a recent report from New York City in the United States where adventive (Ascher and Pickering, 2018, based on an identification by H. Dathe).

***Hylaeus* (*Paraprosopis*) *sinuatus*** (Schenck, 1853)

The species delimitation needs further study.

References: Scheuchl and Willner (2016).

Distribution: Widespread in Europe, Western Asia, Central Asia (Iran).

***Lasioglossum* (*Lasioglossum*) *bischoffi*** (Blüthgen, 1931)

Ebmer (2014) does not report this species for Cyprus. The record refers to the later described *L. aphrodite.*

References: Ebmer (1988).

Distribution: Cyprus, Southern Europe (Croatia, Serbia, Greece), Eastern Europe (Hungary), Western Asia (Turkey, Israel).

***Lasioglossum* (*Hemihalictus*) *crassepunctatum*** (Blüthgen, 1923)

References: Ebmer (2000); Scheuchl and Willner (2016). However, not recorded in Ebmer (2014) and thus regarded as uncertain here.

Distribution: Western Europe (France, Austria), Southern Europe, Eastern Europe.

***Lasioglossum* (*Dialictus*) *nitidulum*** (Fabricius, 1804)

Not recorded by Ebmer (2014) so regarded here as uncertain.

References: Scheuchl and Willner (2016).

Distribution: Widespread in Europe.

***Lasioglossum* (*Hemihalictus*) *villosulum*** (Kirby, 1802)

Integrative taxonomy resuscitated two cryptic species in this complex including *L. medinai*, the form verified to occur on Cyprus. *Lasioglossum villosulum villosulum sensu stricto* has been confirmed from Greece including Crete and from Israel, so should be looked for on Cyprus, but there are no verified records (Pauly et al., 2019).

References: Mavromoustakis (1949 [“1948”], 1954); Ebmer (2014).

Distribution: Widespread in Europe, Northern Africa, Central Asia, Eastern Asia.

***Megachile* (*Eutricharaea*) *fertoni*** Pérez, 1895

Part of a poorly known complex where no author has correctly identified the species, so this name is applied provisionally to material cited below. Males have been confused with M. *apicalis* and females with *semicircularis* Zanden and other species.

References: none.

Material examined: Paphos District: 15 km SE Paphos Kouklia, 34.72°N, 32.55°E, 20.VI.2013, (2♂); 20 km NNW Paphos, Lara Beach, 34.94°N, 32.31°E, 20.VI.2013, (1♀); 6 km NE Polis, beach, 35.06°N, 32.46°E, 20.VI.2013, (1♀), all records C. Schmid-Egger leg., C. Praz det.

Distribution: Cyprus, Western Europe (France), Southern Europe (Spain, Italy), Northern Africa.

***Megachile*** (***Chalicodoma***) ***hungarica*** Mocsáry, 1877

This taxon is probably not present in Cyprus. The taxon was described as *Chalicodoma fallax* from Cyprus. It is very likely a synonym of *Megachile roeweri* or *Megachile roeweri akrotirica* if accepting a narrow species concept. A broader species concept of the lefebvrei group (Praz, 2017) would result in other senior synonyms to be the valid species.

Type Locality-Country: Cyprus, Keryneia: J.D. Alfken det.

References: Alfken (1933).

Distribution: Cyprus, Southern Europe (Greece), Eastern Europe (Hungary, Bulgaria, Slovakia).

***Nomada arrogans*** Schmiedeknecht, 1882

Not included in Smit 2018. Excluded from the main list pending re-evaluation of material in the Mavromoustakis collection.

References: Mavromoustakis (1957).

Mavromoustakis localities: Pera Pedi.

Distribution: Southern Europe (Greece), Eastern Europe (Romania, Ukraine), Western Asia (from Turkey to Israel).

***Nomada mauritanica*** Lepeletier, 1841

The only record is from Mavromoustakis (1949 [“1948”]) as ‘‘*N. mauretanica* var. *manni* Friese’’ [the correct author of this taxon is Morawitz]. This taxon is now considered to be a synonym of *Nomada numida* (Smit, 2018), or a subspecies of this.

References: Mavromoustakis (1949 [“1948”]).

Distribution: Cyprus, Western Europe (Switzerland, Germany), Southern Europe, Eastern Europe (Czech Republic), Northern Africa, Western Asia, Central Asia (Turkmenistan).

***Osmia* (*Helicosmia*) *aurulenta*** Panzer, 1799

Not reported for Cyprus in the Palaearctic Osmiine bee checklist [Müller A (2018)].

References: Scheuchl and Willner (2016), where reported as a doubtful record.

Distribution: Widespread in Europe and Western Asia.

***Pseudapis* (*Nomiapis*) *fugax*** (Morawitz, 1877)

This species has been erroneously recorded from Cyprus because of a mis-identification (Ebmer, 2014).

References: Pittioni (1950).

Distribution: Cyprus, Northern Africa (Egypt), Western Asia (Turkey), Central Asia, Southern Asia (Iran).

***Pseudapis* (*Nomiapis*) *monstrosa*** (Costa, 1861)

Reports of this species pertain to *P. valga* (Ebmer, 2014).

References: Mavromoustakis (1949 [“1948”]).

Mavromoustakis localities: Polemedia Hills.

Distribution: Cyprus, Southern Europe, Eastern Europe (Bulgaria, Ukraine), Western Asia (Turkey, Israel, Azerbaijan).

***Stelis* (*Stelis*) *phaeoptera*** (Kirby, 1802)

Warncke (1992) recorded only subspecies *murina* (treated here as a species following Kasparek, 2015). Kaparek (2015) noted regarding *S. murina*, “It is not clear whether, and if so, its range overlaps with that of *S. phaeoptera*” and for this reason we treat the latter’s occurrence as Cyprus as uncertain (despite being mapped for Cyprus in that revision).

References: Popov (1944); Mavromoustakis (1949 [“1948”], 1957a).

Distribution: Cyprus, Widespread in Europe.

***Thyreus scutellaris*** (Fabricius, 1781)

Reported by Pittioni (1950) under its junior synonym *Thyreus crassicornis*. No further records or literature reports of this species for Cyprus are known and its status there is doubtful. Lieftinck (1968) did not record it from Cyprus but noted a potentially wide range in Western Asia and the eastern Mediterranean.

References: Pittioni (1950).

Distribution: Southern Europe (Croatia), Western and Central Asia, Eastern Asia (China).

Table S1. Acronyms of museum / personal collections where specimens are located.

| AMNH: American Museum of Natural History, United States |
| --- |
| AWE: Andreas Werner Ebmer private collection, Austria |
| DAAN: Department of Agriculture, Nicosia, Cyprus |
| ETHZ: Eidgenössische Technische Hochschule, Zürich, Switzerland |
| FSAG: Zoologie Generale et Appliquée, Belgium |
| KW: Klaus Warncke private collection formerly in Germany now in Austria |
| MFNB: Museo Friulano di Storia Naturale, Italy |
| MNHN: Muséum National d'Histoire Naturelle, Paris, France |
| MSAA: Maximilian Schwarz private collection in Ansfelden (near Linz) in Austria |
| MZHF: University of Helsinki, Zoological Museum, Finland |
| NHMUK: Natural History Museum, UK |
| OLML: Oberösterreichisches Landesmuseum, Linz, Austria |
| SMNS: Staatliches Museum für Naturkunde, Germany |
| UAEG: University of the Aegean, Lesvos, Greece |
| ZISP: Zoological Institute, Russian Academy of Sciences, Russia |

Table S2. Grouping of countries into geographic regions based on United Nations, Department of Economic and Social Affairs, Population Division (2017) (ST/ESA/SER.A/408).

| Western Europe: Austria, Belgium, France, Germany, Liechtenstein, Luxembourg, Monaco, Netherlands, Switzerland. |
| --- |
| Southern Europe: Albania, Andorra, Bosnia and Herzegovina, Croatia, Gibraltar, Greece, Holy See, Italy, Malta, Portugal, San Marino, Slovenia, Spain, The former Yugoslav Republic of Macedonia, Yugoslavia. |
| Northern Europe: Channel Islands, Denmark, Estonia, Faeroe Islands, Finland, Iceland, Ireland, Isle of Man, Latvia, Lithuania, Norway, Sweden, United Kingdom of Great Britain and Northern Ireland. |
| Eastern Europe: Belarus, Bulgaria, Czech Republic, Hungary, Poland, Republic of Moldova, Romania, Russian Federation, Slovakia, Ukraine. |
| Western Africa: Benin, Burkina Faso, Cape Verde, Cote d’Ivoire, Gambia, Ghana, Guinea, Guinea-Bissau, Liberia, Mali, Mauritania, Niger, Nigeria, St. Helena, Senegal, Sierra Leone, Togo. |
| Southern Africa: Botswana, Lesotho, Namibia, South Africa, Swaziland. |
| Northern Africa: Algeria, Egypt, Libya, Morocco, Sudan, Tunisia, Western Sahara. |
| Eastern Africa: Burundi, Comoros, Djibouti, Eritrea, Ethiopia, Kenya, Madagascar, Malawi, Mauritius, Mozambique, Reunion, Rwanda, Seychelles, Somalia, Uganda, United Republic of Tanzania, Zambia, Zimbabwe. |
| Western Asia: Armenia, Azerbaijan, Bahrain, Cyprus, Georgia, Iraq, Israel, Jordan, Kuwait, Lebanon, Occupied Palestinian Territory, Oman, Qatar, Saudi Arabia, Syrian Arab Republic, Turkey, United Arab Emirates, Yemen. |
| South-eastern Asia: Brunei Darussalam, Cambodia, East Timor, Indonesia, Lao People’s Republic, Malaysia, Myanmar, Philippines, Singapore, Thailand, Viet Nam. |
| Central Asia: Kazakhstan, Kyrgyzstan, Tajikistan, Turkmenistan, Uzbekistan. |
| Southern Asia: Afghanistan, Bangladesh, Bhutan, India, Iran, Maldives, Nepal, Pakistan, Sri Lanka. |
| Eastern Asia: China, Hong Kong SAR, Democratic People’s Republic of Korea, Japan, Mongolia, Republic of Korea. |

Table S3. Geographic coordinates for type specimens from Cyprus (see Fig. 1 for the map). The first column indicates whether the nomenclature is currently valid (V), or whether the taxon has been synonymised (S). Taxa described from Cyprus as subspecies are reported as such to indicate the potentially interesting variation within the taxon. See main text for the species name under which synonymised specimens were described. Coordinates for type specimens do not represent exact locations, but the general area of the location from which the species was described, as no geographic coordinates were recorded at the time of description.

| Species | V/S | Geographic coordinates | |
| --- | --- | --- | --- |
| *Ammobates* (*Ammobates*) *biastoides globosus* | V | 34.65°N | 32.975°E |
| *Ammobates* (*Ammobates*) *mavromoustakisi*  *mavromoustakisi* | V | 34.65635°N | 32.98748°E |
| *Ammobates* (*Ammobates*) *mavromoustakisi*  *mavromoustakisi* | V | 34.72075°N | 33.05327°E |
| *Andrena* (*Aciandrena*) *tenuiformis* | V | 34.9364°N | 32.8636°E |
| *Andrena* (*Aenandrena*) *chaetogastra* | V | 34.9364°N | 32.8636°E |
| *Andrena* (*Chlorandrena*) *crepidis* | V | 34.904°N | 32.761°E |
| *Andrena* (*Chlorandrena*) *panurgimorpha* | V | 34.66839°N | 33.03252°E |
| *Andrena* (*Chrysandrena*) *merula* | V | 34.9816°N | 32.9712°E |
| *Andrena* (*Cordandrena*) *cypria* | V | 34.9364°N | 32.8636°E |
| *Andrena* (*Margandrena*) *hyacinthina* | V | 34.7182°N | 33.08788°E |
| *Andrena* (*Margandrena*) *sibthorpi* | V | 34.7134°N | 32.9812°E |
| *Andrena* (*Micrandrena*) *cervina* | V | 34.66839°N | 33.03252°E |
| *Andrena* (*Micrandrena*) *lindbergella* | V | 34.9364°N | 32.8636°E |
| *Andrena* (*Notandrena*) *ungeri* | V | 34.66395°N | 32.99979°E |
| *Andrena* (*Plastandrena*) *cypricola* | V | 34.9059°N | 32.9431°E |
| *Andrena* (*Plastandrena*) *oligotricha* | V | 34.859444°N | 32.876111°E |
| *Andrena* (*Poecilandrena*) *limassolica* | V | 34.66839°N | 33.03252°E |
| *Andrena* (*Ptilandrena*) *kornosica* | V | 35.33253°N | 33.11675°E |
| *Andrena* (*Troandrena*) *saettana* | V | 34.8708333°N | 32.9166667°E |
| *Andrena* (*Truncandrena*) *pareklisiae* | V | 34.82444°N | 32.92083°E |
| *Andrena* (*Ulandrena*) *neocypriaca* | V | 34.66839°N | 33.03252°E |
| *Andrena* (*Ulandrena*) *polemediana* | V | 34.9816°N | 32.9712°E |
| *Andrena* (*Truncandrena*) *rufomaculata*  *paramythensis* | V | 34.757°N | 32.972°E |
| *Anthidiellum* (*Anthidiellum*) *breviusculum*  *troodicum* | V | 34.908°N | 32.866°E |
| *Anthidium* (*Proanthidium*) *undulatum*  *holozonium* | V | 34.66839°N | 33.03252°E |
| *Anthophora* (*Lophanthophora*) *rutilans* | V | 35.166667°N | 33.366667°E |
| *Ceratina* (*Euceratina*) *cypriaca* | V | 34.858°N | 32.873°E |
| *Chelostoma* (*Chelostoma*) *comosum* | V | 34.8775°N | 32.8386°E |
| *Chelostoma* (*Chelostoma*) *diodon cypriacum* | V | 34.8025°N | 32.998056°E |
| *Colletes cyprius* | V | 34.984°N | 32.741°E |
| *Dufourea* (*Cyprirorophites*) *cypria* | V | 35.125°N | 33.941667°E |
| *Eucera* (*Eucera*) *cypria* | V | 34.66839°N | 33.03252°E |
| *Eucera* (*Synhalonia*) *mavromoustakisi* | V | 34.82444°N | 32.92083°E |
| *Halictus* (*Halictus*) *nicosiae* | V | 35.166667°N | 33.366667°E |
| *Halictus* (*Seladonia*) *cypricus* | V | 34.66839°N | 33.03252°E |
| *Halictus* (*Seladonia*) *pollinosus limissicus* | V | 34.66839°N | 33.03252°E |
| *Heriades* (*Michenerella*) *punctulifera troodica* | V | 34.9234°N | 32.8833°E |
| *Hoplitis* (*Alcidamea*) *limassolica* | V | 34.66839°N | 33.03252°E |
| *Hoplitis* (*Anthocopa*) *cypriaca* | V | 34.66839°N | 33.03252°E |
| *Hoplitis* (*Hoplitis*) *holmboei* | V | 34.7182°N | 33.08788°E |
| *Hoplitis* (*Anthocopa*) *yermasoyiae yermasoyiae* | V | 34.7182°N | 33.08788°E |
| *Lasioglossum* (*Dialictus*) *akroundicum* | V | 34.768889°N | 33.079444°E |
| *Lasioglossum* (*Lasioglossum*) *aphrodite* | V | 34.9045°N | 32.86849°E |
| *Lasioglossum* (*Hemihalictus*) *laevidorsum*  *troodicum* | V | 34.9234°N | 32.8833°E |
| *Lasioglossum* (*Lasioglossum*) *pallens kantarae* | V | 35.4064°N | 33.9233°E |
| *Lasioglossum* (*Sphecodogastra***)** *tricinctum*  *lonicerae* | V | 34.9234°N | 32.8833°E |
| *Megachile* (*Chalicodoma*) *cypricola* | V | 34.7198°N | 33.0195°E |
| *Megachile* (*Eutricharaea*) *posti* | V | 34.69944°N | 32.996944°E |
| *Megachile* (*Creightonella*) *albisecta cyprica* | V | 34.66839°N | 33.03252°E |
| *Megachile* (*Chalicodoma*) *roeweri akrotirica* | V | 34.5843°N | 32.9676°E |
| *Nomada cherkesiana* | V | 34.65°N | 32.975°E |
| *Nomada cypria* | V | 34.65°N | 32.975°E |
| *Nomada cypricola* | V | 34.6563°N | 33.0029°E |
| *Nomada cypricola* | V | 34.66839°N | 33.03252°E |
| *Nomada gageae* | V | 34.66839°N | 33.03252°E |
| *Nomada kornosica* | V | 35.33253°N | 33.11675°E |
| *Nomada limassolica* | V | 34.66839°N | 33.03252°E |
| *Nomada nesiotica* | V | 34.761667°N | 33.026944°E |
| *Nomada polemediana* | V | 34.66839°N | 33.03252°E |
| *Nomada pyrgosica* | V | 35.61877°N | 34.38956°E |
| *Nomada teunisseni* | V | 35.3741°N | 33.7558°E |
| *Nomada stigma cypricola* | V | 34.6563°N | 33.0029°E |
| *Osmia* (*Pyrosmia*) *amathusica* | V | 34.7125°N | 33.1419°E |
| *Osmia* (*Osmia*) *cornuta neoregaena* | V | 34.8708333°N | 32.9166667°E |
| *Osmia* (*Pyrosmia*) *viridana nicosiana* | V | 34.71947°N | 33.01962°E |
| *Sphecodes croaticus cypricus* | V | 34.66839°N | 33.03252°E |
| *Aglaoapis tridentata* | S | 34.7134°N | 32.9812°E |
| *Andrena* (*Brachyandrena*) *colletiformis* | S | 34.6978°N | 32.592°E |
| *Andrena* (*Cryptandrena*) *ventricosa* | S | 34.65°N | 32.975°E |
| *Andrena* (*Euandrena*) *bicolor* | S | 34.66839°N | 33.03252°E |
| *Andrena* (*Melanapis*) *fuscosa* | S | 35.166667°N | 33.366667°E |
| *Andrena* (*Melandrena*) *limata* | S | 35.166667°N | 33.366667°E |
| *Andrena* (*Melandrena*) *morio* | S | 34.66839°N | 33.03252°E |
| *Andrena* (*Melandrena*) *thoracica* | S | 34.66839°N | 33.03252°E |
| *Andrena* (*Poliandrena*) *pyrozonata* | S | 34.859444°N | 32.876111°E |
| *Andrena* (*Suandrena*) *aegypticola* | S | 35.125°N | 33.941667°E |
| *Andrena* (*Taeniandrena*) *similis* | S | 34.66839°N | 33.03252°E |
| *Andrena* (*Truncandrena*) *caneae* | S | 34.76666°N | 32.46666°E |
| *Anthidium* (*Anthidium*) *florentinum* | S | 34.86777°N | 32.69027°E |
| *Anthophora* (*Anthophora*) *plumipes* | S | 34.7125°N | 33.1419°E |
| *Chiasmognathus orientanus* | S | 34.7182°N | 33.08788°E |
| *Coelioxys* (*Allocoelioxys*) *acanthopyga* | S | 34.65°N | 32.975°E |
| *Dioxys pumilus* | S | 34.66839°N | 33.03252°E |
| *Eoanthidium* (*Eoanthidium*) *insulare* | S | 34.66839°N | 33.03252°E |
| *Eucera* (*Synhalonia*) *zeta* | S | 34.9075°N | 32.989444°E |
| *Hoplitis* (*Anthocopa*) *fasciculata* | S | 34.7182°N | 33.08788°E |
| *Hylaeus* (*Dentigera*) *imparilis* | S | 34.67513°N | 32.8826°E |
| *Hylaeus* (*Dentigera*) *imparilis* | S | *34.72231°N* | 33.08497°E |
| *Hylaeus* (*Dentigera*) *rubicola* | S | 34.7182°N | 33.08788°E |
| *Hylaeus* (*Paraprosopis*) *lineolatus* | S | 34.984°N | 32.741°E |
| *Hylaeus* (*Prosopis*) *meridionalis* | S | 34.65°N | 32.975°E |
| *Hylaeus* (*Spatulariella*) *cypricola* | S | 34.66839°N | 33.03252°E |
| *Lasioglossum* (*Lasioglossum*) *kotschyi* | S | 34.9045°N | 32.86849°E |
| *Megachile* (*Anodonteutricharaea*) *troodica* | S | 34.9234°N | 32.8833°E |
| *Megachile* (*Megachile*) *centuncularis* | S | 34.66839°N | 33.03252°E |
| *Megachile* (*Megachile*) *melanopyga* | S | 34.9473°N | 32.9284°E |
| *Melecta* (*Melecta*) *mundula* | S | 34.699444°N | 32.996944°E |
| *Osmia* (*Pyrosmia*) *saxicola* | S | 34.859444°N | 32.876111°E |
| *Sphecodes* (*Sphecodes*) *alternatus* | S | 34.76666°N | 32.46666°E |
| *Sphecodes* (*Sphecodes*) *gibbus* | S | 34.67513°N | 32.8826°E |
| *Sphecodes* (*Sphecodes*) *monilicornis* | S | 34.9364°N | 32.8636°E |
| *Tarsalia ancyliformis* | S | 34.7559°N | 32.4516°E |
| *Tarsalia hirtipes* | S | 34.65°N | 32.975°E |

**Table S4:** Geographic coordinates for unpublished records of bee species (see Fig. 1 for the map).

| **Species** | **Geographic coordinates** | |
| --- | --- | --- |
| *Eucera (Hetereucera) bidentata* | 34.963264°N | 34.066211°E |
| *Amegilla* (*Amegilla*) *quadrifasciata* | 34.9333°N | 32.9004°E |
| *Amegilla* (*Zebramegilla*) *albigena* | 34.9333°N | 32.9004°E |
| *Amegilla* (*Zebramegilla*) *albigena* | 34.992°N | 32.9082°E |
| *Andrena* (*Aciandrena*) *aciculata* | 34.597305°N | 32.984521°E |
| *Andrena* (*Aciandrena*) *aciculata* | 34.600657°N | 32.971419°E |
| *Andrena* (*Aciandrena*) *aciculata* | 34.71178°N | 33.004775°E |
| *Andrena* (*Aciandrena*) *aciculata* | 34.764269°N | 32.757736°E |
| *Andrena* (*Aciandrena*) *lamiana* | 34.741952°N | 32.734845°E |
| *Andrena* (*Chlorandrena*) *crepidis* | 34.93°N | 32.78°E |
| *Andrena* (*Chlorandrena*) *crepidis* | 34.918°N | 32.9472°E |
| *Andrena* (*Chlorandrena*) *crepidis* | 34.992°N | 32.9082°E |
| *Andrena* (*Chlorandrena*) *gordia* | 34.883435°N | 32.750988°E |
| *Andrena* (*Chlorandrena*) *orientana* | 34.600657°N | 32.971419°E |
| *Andrena* (*Chlorandrena*) *orientana* | 34.748126°N | 32.732248°E |
| *Andrena* (*Chlorandrena*) *panurgimorpha* | 34.764269°N | 32.757736°E |
| *Andrena* (*Chrysandrena*) *hesperia* | 34.600657°N | 32.971419°E |
| *Andrena* (*Chrysandrena*) *hesperia* | 34.918°N | 32.9472°E |
| *Andrena* (*Chrysandrena*) *hesperia* | 34.656698°N | 32.773339°E |
| *Andrena* (*Cordandrena*) *torda* | 34.963264°N | 34.066211°E |
| *Andrena* (*Cryptandrena*) *brumanensis* | 34.755799°N | 33.096194°E |
| *Andrena* (*Cryptandrena*) *brumanensis* | 34.745537°N | 32.73385°E |
| *Andrena* (*Cryptandrena*) *monacha* | 34.755799°N | 33.096194°E |
| *Andrena* (*Euandrena*) *bicolor* | 34.9317°N | 32.8664°E |
| *Andrena* (*Holandrena*) *variabilis* | 34.764269°N | 32.757736°E |
| *Andrena* (*Holandrena*) *variabilis* | 34.9333°N | 32.9004°E |
| *Andrena* (*Margandrena*) *hyacinthina* | 34.755799°N | 33.096194°E |
| *Andrena* (*Melanapis*) *fuscosa* | 34.963264°N | 34.066211°E |
| *Andrena* (*Melandrena*) *elmaria* | 34.963264°N | 34.066211°E |
| *Andrena* (*Melandrena*) *elmaria* | 34.755799°N | 33.096194°E |
| *Andrena* (*Melandrena*) *limata* | 34.600657°N | 32.971419°E |
| *Andrena* (*Melandrena*) *morio* | 34.963264°N | 34.066211°E |
| *Andrena* (*Melandrena*) *morio* | 34.71178°N | 33.004775°E |
| *Andrena* (*Melandrena*) *morio* | 34.992°N | 32.9082°E |
| *Andrena* (*Melandrena*) *nigroaenea* | 34.9317°N | 32.8664°E |
| *Andrena* (*Melandrena*) *nigroaenea* | 34.9095°N | 32.8971°E |
| *Andrena* (*Melandrena*) *nigroaenea* | 34.918°N | 32.9472°E |
| *Andrena* (*Melandrena*) *nigroaenea* | 34.9333°N | 32.9004°E |
| *Andrena* (*Melandrena*) *nigroaenea* | 34.992°N | 32.9082°E |
| *Andrena* (*Micrandrena*) *alfkenelloides* | 34.764269°N | 32.757736°E |
| *Andrena* (*Micrandrena*) *alfkenelloides* | 34.745537°N | 32.73385°E |
| *Andrena* (*Micrandrena*) *alfkenelloides* | 34.677579°N | 32.722066°E |
| *Andrena* (*Micrandrena*) *cervina* | 34.963264°N | 34.066211°E |
| *Andrena* (*Micrandrena*) *cervina* | 34.748126°N | 32.732248°E |
| *Andrena* (*Parandrenella*) *nisoria* | 34.755799°N | 33.096194°E |
| *Andrena* (*Plastandrena*) *cypricola* | 34.992°N | 32.9082°E |
| *Andrena* (*Plastandrena*) *cypricola* | 35.0755°N | 32.9164°E |
| *Andrena* (*Plastandrena*) *oligotricha* | 34.748126°N | 32.732248°E |
| *Andrena* (*Plastandrena*) *oligotricha* | 34.883435°N | 32.750988°E |
| *Andrena* (*Ptilandrena*) *glidia* | 34.9333°N | 32.9004°E |
| *Andrena* (*Ptilandrena*) *glidia* | 34.992°N | 32.9082°E |
| *Andrena* (*Ptilandrena*) *glidiaWarncke* | 34.9317°N | 32.8664°E |
| *Andrena* (*Ptilandrena*) *vetula* | 34.963264°N | 34.066211°E |
| *Andrena* (*Ptilandrena*) *vetula* | 34.764269°N | 32.757736°E |
| *Andrena* (*Ptilandrena*) *vetula* | 34.73663°N | 32.732715°E |
| *Andrena* (*Ptilandrena*) *vetula* | 34.741952°N | 32.734845°E |
| *Andrena* (*Ptilandrena*) *vetula* | 34.723986°N | 32.736892°E |
| *Andrena* (*Ptilandrena*) *vetula* | 34.661805°N | 32.804261°E |
| *Andrena* (*Ptilandrena*) *vetula* | 34.883435°N | 32.750988°E |
| *Andrena* (*Simandrena*) *combinata* | 34.9095°N | 32.8971°E |
| *Andrena* (*Taeniandrena*) *leucopsis* | 34.9333°N | 32.9004°E |
| *Andrena* (*Taeniandrena*) *ovatula* | 34.755799°N | 33.096194°E |
| *Andrena* (*Taeniandrena*) *similis* | 34.755799°N | 33.096194°E |
| *Andrena* (*Taeniandrena) similis* | 34.883435°N | 32.750988°E |
| *Andrena* (*Truncandrena*) *truncatilabris* | 34.71178°N | 33.004775°E |
| *Andrena* (*Truncandrena*) *truncatilabris* | 34.597305°N | 32.984521°E |
| *Andrena* (*Truncandrena*) *truncatilabris* | 34.755799°N | 33.096194°E |
| *Andrena* (*Truncandrena*) *truncatilabris* | 34.883435°N | 32.750988°E |
| *Andrena* (*Ulandrena*) *neocypriaca* | 34.748126°N | 32.732248°E |
| *Andrena* (*Ulandrena*) *polemediana* | 34.741952°N | 32.734845°E |
| *Andrena* (*Zonandrena*) *flavipes* | 34.764269°N | 32.757736°E |
| *Andrena* (*Zonandrena*) *flavipes* | 34.9317°N | 32.8664°E |
| *Andrena* (*Zonandrena*) *flavipes* | 34.9095°N | 32.8971°E |
| *Andrena* (*Zonandrena*) *flavipes* | 34.918°N | 32.9472°E |
| *Andrena* (*Zonandrena*) *flavipes* | 34.9333°N | 32.9004°E |
| *Andrena* (*Zonandrena*) *flavipes* | 34.755799°N | 33.096194°E |
| *Andrena* (*Zonandrena*) *flavipes* | 34.992°N | 32.9082°E |
| *Andrena* (*Zonandrena*) *flavipes* | 34.883435°N | 32.750988°E |
| *Andrena* (*Zonandrena*) *flavipes* | 34.729004°N | 32.457544°E |
| *Anthidium* (*Anthidium*) *cingulatum* | 34.670772°N | 32.846923°E |
| *Anthidium* (*Anthidium*) *diadema* | 34.670772°N | 32.846923°E |
| *Anthophora* (*Anthophora*) *canescens* | 34.7626°N | 32.4108°E |
| *Anthophora* (*Anthophora*) *plumipes* | 34.9095°N | 32.8971°E |
| *Anthophora* (*Anthophora*) *plumipes* | 34.992°N | 32.9082°E |
| *Anthophora* (*Lophanthophora*) *rutilans* | 34.7626°N | 32.4108°E |
| *Anthophora* (*Pyganthophora*) *dalmatica* | 34.9333°N | 32.9004°E |
| *Anthophora* (*Pyganthophora*) *rubricrus* | 34.7626°N | 32.4108°E |
| *Bombus* (*Bombus*) *terrestris* | 34.9095°N | 32.8971°E |
| *Bombus* (*Bombus*) *terrestris* | 34.9317°N | 32.8664°E |
| *Bombus* (*Bombus*) *terrestris* | 34.918°N | 32.9472°E |
| *Bombus* (*Bombus*) *terrestris* | 34.9333°N | 32.9004°E |
| *Bombus* (*Bombus*) *terrestris* | 34.656698°N | 32.773339°E |
| *Bombus* (*Bombus*) *terrestris* | 34.670772°N | 32.846923°E |
| *Bombus* (*Bombus*) *terrestris* | 34.93°N | 32.86°E |
| *Bombus* (*Bombus*) *terrestris* | 34.992°N | 32.9082°E |
| *Bombus* (*Bombus*) *terrestris* | 35.0755°N | 32.9164°E |
| *Ceratina* (*Euceratina*) *mandibularis* | 34.729004°N | 32.457544°E |
| *Ceylalictus* (*Ceylalictus*) *variegatus* | 34.731233°N | 33.343487°E |
| *Ceylalictus* (*Ceylalictus*) *variegatus* | 34.729004°N | 32.457544°E |
| *Ceylalictus* (*Ceylalictus*) *variegatus* | 35.037305°N | 32.391073°E |
| *Chelostoma* (*Chelostoma*) *comosum* | 34.73663°N | 32.732715°E |
| *Chelostoma* (*Chelostoma*) *diodon* | 34.764269°N | 32.757736°E |
| *Coelioxys* (*Allocoelioxys*) *afer* | 34.93°N | 32.86°E |
| *Colletes brevigena* | 35.05519°N | 33.814011°E |
| *Colletes brevigena* | 34.731233°N | 33.343487°E |
| *Colletes brevigena* | 35 00.598°N | 33 35.041°E |
| *Colletes brevigena* | 34.746277°N | 33.384472°E |
| *Colletes brevigena* | 34.77458°N | 33.448023°E |
| *Colletes brevigena* | 34.600657°N | 32.971419°E |
| *Colletes brevigena* | 34.9317°N | 32.8664°E |
| *Colletes brevigena* | 34.9095°N | 32.8971°E |
| *Colletes brevigena* | 34.745849°N | 33.083579°E |
| *Colletes brevigena* | 34.745849°N | 33.083579°E |
| *Colletes brevigena* | 34.676011°N | 32.794947°E |
| *Colletes brevigena* | 34.743911°N | 32.732439°E |
| *Colletes brevigena* | 34.729369°N | 32.738368°E |
| *Colletes brevigena* | 34.742646°N | 32.730431°E |
| *Colletes brevigena* | 34 52.892°N | 32 52.618°E |
| *Colletes* *brevigena* | 34.992°N | 32.9082°E |
| *Colletes brevigena* | 34 57.711°N | 32 49.733°E |
| *Colletes brevigena* | 34 58.308°N | 32 49.775°E |
| *Colletes brevigena* | 35.053539°N | 32.351197°E |
| *Colletes brevigena* | 35.036538°N | 32.373117°E |
| *Colletes brevigena* | 35.037305°N | 32.391073°E |
| *Colletes brevigena* | 35.032203°N | 32.413732°E |
| *Colletes brevigena* | 34.810747°N | 32.450907°E |
| *Colletes creticus* | 34.670772°N | 32.846923°E |
| *Colletes creticus* | 34.654385°N | 32.717924°E |
| *Colletes* *cyprius* | 34.729369°N | 32.738368°E |
| *Colletes cyprius* | 34.72°N | 32.55°E |
| *Colletes cyprius* | 35.053539°N | 32.351197°E |
| *Colletes cyprius* | 35.036538°N | 32.373117°E |
| *Colletes similis* | 34.600657°N | 32.971419°E |
| *Colletes similis* | 35.053539°N | 32.351197°E |
| *Eoanthidium* (*Eoanthidium*) *insulare* | 34.9333°N | 32.9004°E |
| *Eucera (Hetereucera) bidentata* | 35.05519°N | 33.814011°E |
| *Eucera (Hetereucera) bidentata* | 35.334413°N | 33.494187°E |
| *Eucera (Hetereucera) bidentata* | 34.653067°N | 32.974233°E |
| *Eucera (Hetereucera) bidentata* | 34.65096°N | 32.99091°E |
| *Eucera (Hetereucera) bidentata* | 34.628817°N | 32.940667°E |
| *Eucera (Hetereucera) caerulescens* | 35.64°N | 34.55°E |
| *Eucera (Hetereucera) caerulescens* | 35.63°N | 34.5°E |
| *Eucera (Hetereucera) caerulescens* | 35.334413°N | 33.494187°E |
| *Eucera* (*Eucera*) *cypria* | 34.65096°N | 32.99091°E |
| *Eucera* (*Eucera*) *dalmatica* | 35.334413°N | 33.494187°E |
| *Eucera* (*Eucera*) *dalmatica* | 34.628817°N | 32.940667°E |
| *Eucera* (*Eucera*) *dimidiata* | 34.963264°N | 34.066211°E |
| *Eucera* (*Eucera*) *dimidiata* | 35.05519°N | 33.814011°E |
| *Eucera* (*Eucera*) *dimidiata* | 34.777521°N | 33.33622°E |
| *Eucera* (*Eucera*) *dimidiata* | 34.653067°N | 32.974233°E |
| *Eucera* (*Eucera*) *dimidiata* | 34.65096°N | 32.99091°E |
| *Eucera* (*Eucera*) *dimidiata* | 34.628817°N | 32.940667°E |
| *Eucera* (*Eucera*) *dimidiata* | 35.1688°N | 33.367°E |
| *Eucera (Hetereucera) gaullei* | 35.1688°N | 33.367°E |
| *Eucera* (*Eucera*) *proxima* | 35.64°N | 34.55°E |
| *Eucera* (*Eucera*) *proxima* | 35.334413°N | 33.494187°E |
| *Eucera* (*Eucera*) *proxima* | 34.9317°N | 32.8664°E |
| *Eucera* (*Eucera*) *proxima* | 34.628817°N | 32.940667°E |
| *Eucera* (*Eucera*) *sulamita* | 35.64°N | 34.55°E |
| *Eucera* (*Eucera*) *sulamita* | 35.63°N | 34.5°E |
| *Eucera* (*Eucera*) *sulamita* | 35.19°N | 33.9°E |
| *Eucera (Hetereucera) syriaca* | 35.64°N | 34.55°E |
| *Eucera (Hetereucera) syriaca* | 35.63°N | 34.5°E |
| *Halictus* (*Halictus*) *asperulus* | 34.72°N | 32.55°E |
| *Halictus* (*Halictus*) *brunnescens* | 34.918°N | 32.9472°E |
| *Halictus* (*Halictus*) *brunnescens* | 34.9333°N | 32.9004°E |
| *Halictus* (*Halictus*) *brunnescens* | 34.6°N | 32.97°E |
| *Halictus* (*Halictus*) *brunnescens* | 34.992°N | 32.9082°E |
| *Halictus* (*Halictus*) *brunnescens* | 35.0755°N | 32.9164°E |
| *Halictus* (*Halictus*) *brunnescens* | 34.94°N | 32.31°E |
| *Halictus* (*Halictus*) *nicosiae* | 34.764269°N | 32.757736°E |
| *Halictus* (*Halictus*) *nicosiae* | 34.723986°N | 32.736892°E |
| *Halictus* (*Halictus*) *nicosiae* | 34.745537°N | 32.73385°E |
| *Halictus* (*Halictus*) *nicosiae* | 34.748126°N | 32.732248°E |
| *Halictus* (*Halictus*) *nicosiae* | 34.93°N | 32.86°E |
| *Halictus* (*Halictus*) *nicosiae* | 34.72°N | 32.55°E |
| *Halictus* (*Halictus*) *nicosiae* | 34.85°N | 32.4°E |
| *Halictus* (*Halictus*) *quadricinctus* | 34.677579°N | 32.722066°E |
| *Halictus* (*Halictus*) *quadricinctus* | 34.748126°N | 32.732248°E |
| *Halictus* (*Halictus*) *resurgens* | 34.731233°N | 33.343487°E |
| *Halictus* (*Halictus*) *resurgens* | 34.628771°N | 32.941031°E |
| *Halictus* (*Halictus*) *resurgens* | 34.656698°N | 32.773339°E |
| *Halictus* (*Halictus*) *resurgens* | 34.73663°N | 32.732715°E |
| *Halictus* (*Halictus*) *resurgens* | 34.745537°N | 32.73385°E |
| *Halictus* (*Halictus*) *resurgens* | 34.748126°N | 32.732248°E |
| *Halictus* (*Halictus*) *resurgens* | 34.85°N | 32.4°E |
| *Halictus* (*Halictus*) *resurgens* | 34.94°N | 32.31°E |
| *Halictus* (*Halictus*) *tetrazonianellus* | 34.677579°N | 32.722066°E |
| *Halictus* (*Halictus*) *tetrazonianellus* | 34.72°N | 32.55°E |
| *Halictus* (*Halictus*) *tetrazonianellus* | 34.9°N | 32.42°E |
| *Halictus* (*Halictus*) *tetrazonianellus* | 34.94°N | 32.31°E |
| *Halictus* (*Seladonia*) *cephalicus* | 34.628771°N | 32.941031°E |
| *Halictus* (*Seladonia*) *cephalicus* | 34.764269°N | 32.757736°E |
| *Halictus* (*Seladonia*) *cephalicus* | 34.677579°N | 32.722066°E |
| *Halictus* (*Seladonia*) *cephalicus* | 34.93°N | 32.86°E |
| *Halictus* (*Seladonia*) *cephalicus* | 34.72°N | 32.55°E |
| *Halictus* (*Seladonia*) *cephalicus* | 35.03°N | 32.37°E |
| *Halictus* (*Seladonia*) *cephalicus* | 35.053539°N | 32.351197°E |
| *Halictus* (*Seladonia*) *cephalicus* | 34.85°N | 32.4°E |
| *Halictus* (*Seladonia*) *cephalicus* | 34.9°N | 32.42°E |
| *Halictus* (*Seladonia*) *cephalicus* | 35.06°N | 32.46°E |
| *Halictus* (*Seladonia*) *cypricus* | 34.72°N | 32.55°E |
| *Halictus* (*Seladonia*) *phryganicus* | 34.670772°N | 32.846923°E |
| *Halictus* (*Seladonia*) *phryganicus* | 34.6°N | 32.97°E |
| *Halictus* (*Seladonia*) *phryganicus* | 34.72°N | 32.55°E |
| *Halictus* (*Seladonia*) *phryganicus* | 35.03°N | 32.37°E |
| *Halictus* (*Seladonia*) *phryganicus* | 34.85°N | 32.4°E |
| *Halictus* (*Seladonia*) *phryganicus* | 35.06°N | 32.46°E |
| *Halictus* (*Seladonia*) *pollinosus* | 34.764269°N | 32.757736°E |
| *Halictus* (*Seladonia*) *pollinosus* | 34.918°N | 32.9472°E |
| *Halictus* (*Seladonia*) *pollinosus* | 34.729004°N | 32.457544°E |
| *Halictus* (*Seladonia*) *pulvereus* | 34.9°N | 32.42°E |
| *Heriades* (*Heriades*) *rubicola* | 34.628771°N | 32.941031°E |
| *Heriades* (*Heriades*) *rubicola* | 34.653067°N | 32.974233°E |
| *Heriades* (*Heriades*) *rubicola* | 34.6432°N | 32.9952°E |
| *Heriades* (*Heriades*) *rubicola* | 34.65096°N | 32.99091°E |
| *Heriades* (*Heriades*) *rubicola* | 34.628817°N | 32.940667°E |
| *Heriades* (*Heriades*) *rubicola* | 35.053539°N | 32.351197°E |
| *Heriades* (*Heriades*) *rubicola* | 34.94°N | 32.31°E |
| *Heriades* (*Heriades*) *truncorum* | 34.653067°N | 32.974233°E |
| *Heriades* (*Heriades*) *truncorum* | 34.886528°N | 32.862465°E |
| *Heriades* (*Heriades*) *truncorum* | 34.628817°N | 32.940667°E |
| *Heriades* (*Heriades*) *truncorum* | 35.053539°N | 32.351197°E |
| *Heriades (Heriades*) *truncorum* | 34.94°N | 32.31°E |
| *Hoplitis* (*Alcidamea*) *acuticornis* | 34.71178°N | 33.004775°E |
| *Hoplitis* (*Alcidamea*) *acuticornis* | 34.597305°N | 32.984521°E |
| *Hoplitis* (*Alcidamea*) *limassolica* | 34.71178°N | 33.004775°E |
| *Hoplitis* (*Anthocopa*) *fasciculata* | 34.72°N | 32.55°E |
| *Hoplitis* (*Anthocopa*) *yermasoyiae* | 34.764269°N | 32.757736°E |
| *Hoplitis* (*Anthocopa*) *yermasoyiae* | 34.755799°N | 33.096194°E |
| *Hoplitis* (*Anthocopa*) *yermasoyiae* | 34.745537°N | 32.73385°E |
| *Hoplitis* (*Anthocopa*) *yermasoyiae* | 34.677579°N | 32.722066°E |
| *Hoplitis* (*Anthocopa*) *yermasoyiae* | 34.992°N | 32.9082°E |
| *Hoplitis* (*Anthocopa*) *yermasoyiae* | 34.900977°N | 32.776759°E |
| *Hoplitis* (*Hoplitis*) *annulata* | 34.597305°N | 32.984521°E |
| *Hoplitis* (*Hoplitis*) *annulata* | 34.755799°N | 33.096194°E |
| *Hoplitis* (*Hoplitis*) *annulata* | 34.65096°N | 32.99091°E |
| *Hoplitis* (*Hoplitis*) *annulata* | 34.6°N | 32.97°E |
| *Hoplitis* (*Hoplitis*) *annulata* | 34.85°N | 32.4°E |
| *Hoplitis* (*Hoplitis*) *annulata* | 34.720825°N | 32.551994°E |
| *Hoplitis* (*Hoplitis*) *annulata* | 34.94°N | 32.31°E |
| *Hoplitis* (*Hoplitis*) *holmboei* | 34.9333°N | 32.9004°E |
| *Hylaeus* (*Dentigera*) *imparilis* | 34.731233°N | 33.343487°E |
| *Hylaeus* (*Dentigera*) *imparilis* | 34.764269°N | 32.757736°E |
| *Hylaeus* (*Dentigera*) *imparilis* | 34.743911°N | 32.732439°E |
| *Hylaeus* (*Dentigera*) *imparilis* | 34.677579°N | 32.722066°E |
| *Hylaeus* (*Dentigera*) *imparilis* | 34.727647°N | 32.73462°E |
| *Hylaeus* (*Dentigera*) *imparilis* | 34.883435°N | 32.750988°E |
| *Hylaeus* (*Dentigera*) *imparilis* | 34.720825°N | 32.551994°E |
| *Hylaeus* (*Lambdopsis*) *scutellatus* | 34.720825°N | 32.551994°E |
| *Hylaeus* (*Paraprosopis*) *taeniolatus* | 34.731233°N | 33.343487°E |
| *Hylaeus* (*Paraprosopis*) *taeniolatus* | 34.755799°N | 33.096194°E |
| *Hylaeus* (*Paraprosopis*) *taeniolatus* | 34.73663°N | 32.732715°E |
| *Hylaeus* (*Paraprosopis*) *taeniolatus* | 34.741952°N | 32.734845°E |
| *Hylaeus* (*Paraprosopis*) *taeniolatus* | 34.723986°N | 32.736892°E |
| *Hylaeus* (*Paraprosopis*) *taeniolatus* | 34.743911°N | 32.732439°E |
| *Hylaeus* (*Paraprosopis*) *taeniolatus* | 34.745537°N | 32.73385°E |
| *Hylaeus* (*Paraprosopis*) *taeniolatus* | 34.677579°N | 32.722066°E |
| *Hylaeus* (*Paraprosopis*) *taeniolatus* | 34.748126°N | 32.732248°E |
| *Hylaeus* (*Paraprosopis*) *taeniolatus* | 34.883435°N | 32.750988°E |
| *Hylaeus* (*Paraprosopis*) *taeniolatus* | 35.053539°N | 32.351197°E |
| *Hylaeus* (*Paraprosopis*) *taeniolatus* | 34.729004°N | 32.457544°E |
| *Hylaeus* (*Paraprosopis*) *taeniolatus* | 34.720825°N | 32.551994°E |
| *Hylaeus* (*Prosopis*) *gibbus* | 34.628771°N | 32.941031°E |
| *Hylaeus* (*Prosopis*) *gibbus* | 34.729004°N | 32.457544°E |
| *Hylaeus* (*Spatulariella*) *cypricola* | 34.731233°N | 33.343487°E |
| *Hylaeus* (*Spatulariella*) *cypricola* | 34.741952°N | 32.734845°E |
| *Hylaeus* (*Spatulariella*) *cypricola* | 34.670772°N | 32.846923°E |
| *Lasioglossum* (*Dialictus*) *akroundicum* | 34.9317°N | 32.8664°E |
| *Lasioglossum* (*Dialictus*) *akroundicum* | 34.93°N | 32.86°E |
| *Lasioglossum* (*Evylaeus*) *marginatum* | 35.334413°N | 33.494187°E |
| *Lasioglossum* (*Evylaeus*) *marginatum* | 34.73663°N | 32.732715°E |
| *Lasioglossum* (*Evylaeus*) *marginatum* | 34.764269°N | 32.757736°E |
| *Lasioglossum* (*Evylaeus*) *marginatum* | 34.656698°N | 32.773339°E |
| *Lasioglossum* (*Evylaeus*) *marginatum* | 34.745537°N | 32.73385°E |
| *Lasioglossum* (*Evylaeus*) *marginatum* | 34.748126°N | 32.732248°E |
| *Lasioglossum* (*Evylaeus*) *marginatum* | 34.883435°N | 32.750988°E |
| *Lasioglossum* (*Evylaeus*) *marginatum* | 34.729004°N | 32.457544°E |
| *Lasioglossum* (*Hemihalictus*) *convexiusculum* | 34.93°N | 32.86°E |
| *Lasioglossum* (*Hemihalictus*) *griseolum* | 35.06°N | 32.46°E |
| *Lasioglossum* (*Hemihalictus*) *griseolum* | 34.94°N | 32.31°E |
| *Lasioglossum* (*Hemihalictus*) *lucidulum* | 34.72°N | 32.55°E |
| *Lasioglossum* (*Hemihalictus*) *mesosclerum* | 34.72°N | 32.55°E |
| *Lasioglossum* (*Hemihalictus*) *mesosclerum* | 34.85°N | 32.4°E |
| *Lasioglossum* (*Hemihalictus*) *mesosclerum* | 34.94°N | 32.31°E |
| *Lasioglossum* (*Hemihalictus*) *pygmaeum* | 35.334413°N | 33.494187°E |
| *Lasioglossum* (*Hemihalictus*) *pygmaeum* | 34.764269°N | 32.757736°E |
| *Lasioglossum* (*Hemihalictus*) *pygmaeum* | 34.670772°N | 32.846923°E |
| *Lasioglossum* (*Hemihalictus*) *transitorium* | 35.334413°N | 33.494187°E |
| *Lasioglossum* (*Hemihalictus*) *transitorium* | 34.723986°N | 32.736892°E |
| *Lasioglossum* (*Hemihalictus*) *transitorium* | 34.670772°N | 32.846923°E |
| *Lasioglossum* (*Lasioglossum*) *aegyptiellum* | 34.72°N | 32.55°E |
| *Lasioglossum* (*Lasioglossum*) *aphrodite* | 34.9317°N | 32.8664°E |
| *Lasioglossum* (*Lasioglossum*) *kotschyi* | 34.9317°N | 32.8664°E |
| *Lasioglossum* (*Lasioglossum*) *pallens* | 34.748126°N | 32.732248°E |
| *Lasioglossum* (*Lasioglossum*) *pallens* | 34.992°N | 32.9082°E |
| *Lasioglossum* (*Leuchalictus*) *leucozonium* | 34.918°N | 32.9472°E |
| *Lasioglossum* (*Leuchalictus*) *leucozonium* | 34.992°N | 32.9082°E |
| *Lasioglossum* (*Leuchalictus*) *leucozonium* | 35.0755°N | 32.9164°E |
| *Lasioglossum* (*Sphecodogastra*) *anellum* | 34.731233°N | 33.343487°E |
| *Lasioglossum* (*Sphecodogastra*) *anellum* | 34.746277°N | 33.384472°E |
| *Lasioglossum* (*Sphecodogastra*) *anellum* | 34.677579°N | 32.722066°E |
| *Lasioglossum* (*Sphecodogastra*) *anellum* | 34.654385°N | 32.717924°E |
| *Lasioglossum* (*Sphecodogastra*) *anellum* | 34.883435°N | 32.750988°E |
| *Lasioglossum* (*Sphecodogastra*) *anellum* | 34.72°N | 32.55°E |
| *Lasioglossum* (*Sphecodogastra*) *anellum* | 34.85°N | 32.4°E |
| *Lasioglossum* (*Sphecodogastra*) *anellum* | 34.720825°N | 32.551994°E |
| *Lasioglossum* (*Sphecodogastra*) *anellum* | 35.06°N | 32.46°E |
| *Lasioglossum* (*Sphecodogastra*) *anellum* | 34.94°N | 32.31°E |
| *Lasioglossum* (*Sphecodogastra*) *damascenum* | 34.670772°N | 32.846923°E |
| *Lasioglossum* (*Sphecodogastra*) *lineare* | 34.9317°N | 32.8664°E |
| *Lasioglossum* (*Sphecodogastra*) *malachurum* | 34.597305°N | 32.984521°E |
| *Lasioglossum* (*Sphecodogastra*) *malachurum* | 34.72°N | 32.55°E |
| *Lasioglossum* (*Sphecodogastra*) *malachurum* | 34.729004°N | 32.457544°E |
| *Lasioglossum* (*Sphecodogastra*) *malachurum* | 34.94°N | 32.31°E |
| *Lasioglossum* (*Sphecodogastra*) *obscuratum* | 34.9317°N | 32.8664°E |
| *Lasioglossum* (*Sphecodogastra*) *obscuratum* | 34.94°N | 32.31°E |
| *Lasioglossum* (*Sphecodogastra*) *tricinctum* | 35.64°N | 34.55°E |
| *Lasioglossum* (*Sphecodogastra*) *tricinctum* | 34.85°N | 32.4°E |
| *Lasioglossum* (*Sphecodogastra*) *tricinctum* | 34.9°N | 32.42°E |
| *Lasioglossum* (*Sphecodogastra*) *tricinctum* | 34.94°N | 32.31°E |
| *Lithurgus* (*Lithurgus*) *tibialis* | 34.72°N | 32.55°E |
| *Lithurgus* (*Lithurgus*) *tibialis* | 34.9°N | 32.42°E |
| *Megachile* (*Chalicodoma*) *cypricola* | 34.777521°N | 33.33622°E |
| *Megachile* (*Chalicodoma*) *cypricola* | 34.656698°N | 32.773339°E |
| *Megachile* (*Chalicodoma*) *cypricola* | 34.66198333°N | 32.80439444°E |
| *Megachile* (*Chalicodoma*) *cypricola* | 34.706873°N | 33.211916°E |
| *Megachile* (*Chalicodoma*) *cypricola* | 34.706786°N | 33.229364°E |
| *Megachile* (*Chalicodoma*) *ericetorum* | 34.745849°N | 33.083579°E |
| *Megachile* (*Creightonella*) *albisecta* | 34.9°N | 32.42°E |
| *Megachile* (*Eurymella*) *patellimana* | 35.03°N | 32.37°E |
| *Megachile* (*Eurymella*) *patellimana* | 34.85°N | 32.4°E |
| *Megachile* (*Eurymella*) *patellimana* | 34.94°N | 32.31°E |
| *Megachile* (*Eutricharaea*) *apicalis* | 34.6°N | 32.97°E |
| *Megachile* (*Eutricharaea*) *apicalis* | 34.72°N | 32.55°E |
| *Megachile* (*Eutricharaea*) *apicalis* | 35.03°N | 32.37°E |
| *Megachile* (*Eutricharaea*) *apicalis* | 34.94°N | 32.31°E |
| *Megachile* (*Eutricharaea*) *fertoni* | 34.72°N | 32.55°E |
| *Megachile* (*Eutricharaea*) *fertoni* | 35.06°N | 32.46°E |
| *Megachile* (*Eutricharaea*) *fertoni* | 34.94°N | 32.31°E |
| *Megachile* (*Eutricharaea*) *inexspectata* | 35.06°N | 32.46°E |
| *Megachile* (*Eutricharaea*) *marginata* | 34.72°N | 32.55°E |
| *Megachile* (*Eutricharea*) *leachella* | 35.05519°N | 33.814011°E |
| *Megachile* (*Eutricharea*) *leachella* | 34.731233°N | 33.343487°E |
| *Megachile* (*Eutricharea*) *leachella* | 34.600657°N | 32.971419°E |
| *Megachile* (*Eutricharea*) *leachella* | 34.745849°N | 33.083579°E |
| *Megachile* (*Eutricharea*) *leachella* | 34.676011°N | 32.794947°E |
| *Megachile* (*Eutricharea*) *leachella* | 34.93°N | 32.86°E |
| *Megachile* (*Eutricharea*) *leachella* | 34.72°N | 32.55°E |
| *Megachile* (*Eutricharea*) *leachella* | 35.03°N | 32.37°E |
| *Megachile* (*Eutricharea*) *leachella* | 35.053539°N | 32.351197°E |
| *Megachile* (*Eutricharea*) *leachella* | 34.85°N | 32.4°E |
| *Megachile* (*Eutricharea*) *leachella* | 34.9°N | 32.42°E |
| *Megachile* (*Eutricharea*) *leachella* | 34.94°N | 32.31°E |
| *Megachile* (*Eutricharea*) *pilidens* | 34.918°N | 32.9472°E |
| *Megachile* (*Pseudomegachile*) *farinosa* | 34.72°N | 32.55°E |
| *Megachile* (*Pseudomegachile*) *farinosa* | 34.85°N | 32.4°E |
| *Melitturga* (*Melitturga*) *syriaca* | 34.755799°N | 33.096194°E |
| *Melitturga* (*Melitturga*) *syriaca* | 34.900977°N | 32.776759°E |
| *Nomada flavoguttata* | 34.755799°N | 33.096194°E |
| *Nomada fucata* | 34.755799°N | 33.096194°E |
| *Nomada nesiotica* | 34.755799°N | 33.096194°E |
| *Nomada pallispinosa* | 34.71178°N | 33.004775°E |
| *Nomada trispinosa* | 34.71178°N | 33.004775°E |
| *Nomada trispinosa* | 34.755799°N | 33.096194°E |
| *Osmia* (*Allosmia*) *sybarita* | 34.71178°N | 33.004775°E |
| *Osmia* (*Allosmia*) *sybarita* | 34.755799°N | 33.096194°E |
| *Osmia* (*Allosmia*) *sybarita* | 34.800043°N | 33.000918°E |
| *Osmia* (*Erythrosmia*) *erythrogastra* | 34.755799°N | 33.096194°E |
| *Osmia* (*Helicosmia*) *caerulescens* | 34.963264°N | 34.066211°E |
| *Osmia* (*Helicosmia*) *caerulescens* | 34.597305°N | 32.984521°E |
| *Osmia* (*Helicosmia*) *caerulescens* | 34.9317°N | 32.8664°E |
| *Osmia* (*Helicosmia*) *caerulescens* | 34.918°N | 32.9472°E |
| *Osmia* (*Helicosmia*) *caerulescens* | 34.755799°N | 33.096194°E |
| *Osmia* (*Helicosmia*) *caerulescens* | 34.670772°N | 32.846923°E |
| *Osmia* (*Helicosmia*) *caerulescens* | 34.85°N | 32.4°E |
| *Osmia* (*Helicosmia*) *dives* | 34.918°N | 32.9472°E |
| *Osmia* (*Helicosmia*) *dives* | 34.677579°N | 32.722066°E |
| *Osmia* (*Helicosmia*) *dives* | 34.628817°N | 32.940667°E |
| *Osmia* (*Helicosmia*) *dives* | 34.911736°N | 32.327703°E |
| *Osmia* (*Helicosmia*) *latreillei* | 34.71178°N | 33.004775°E |
| *Osmia* (*Helicosmia*) *latreillei* | 34.653067°N | 32.974233°E |
| *Osmia* (*Helicosmia*) *latreillei* | 34.65096°N | 32.99091°E |
| *Osmia* (*Helicosmia*) *latreillei* | 34.73663°N | 32.732715°E |
| *Osmia* (*Helicosmia*) *latreillei* | 34.745537°N | 32.73385°E |
| *Osmia* (*Helicosmia*) *latreillei* | 34.720825°N | 32.551994°E |
| *Osmia* (*Helicosmia*) *niveata* | 34.597305°N | 32.984521°E |
| *Osmia* (*Helicosmia*) *niveata* | 34.918°N | 32.9472°E |
| *Osmia* (*Helicosmia*) *niveata* | 34.65096°N | 32.99091°E |
| *Osmia* (*Helicosmia*) *niveata* | 34.741952°N | 32.734845°E |
| *Osmia* (*Helicosmia*) *signata* | 34.65096°N | 32.99091°E |
| *Osmia* (*Helicosmia*) *signata* | 34.9°N | 32.42°E |
| *Osmia* (*Hoplosmia*) *scutellaris* | 34.65096°N | 32.99091°E |
| *Osmia* (*Osmia*) *bicornis* | 34.992°N | 32.9082°E |
| *Osmia* (*Pyrosmia*) *amathusica* | 34.71178°N | 33.004775°E |
| *Osmia* (*Pyrosmia*) *cephalotes* | 34.73663°N | 32.732715°E |
| *Osmia* (*Pyrosmia*) *ferruginea* | 34.71178°N | 33.004775°E |
| *Osmia* (*Pyrosmia*) *ferruginea* | 34.656698°N | 32.773339°E |
| *Osmia* (*Pyrosmia*) *ferruginea* | 34.755799°N | 33.096194°E |
| *Osmia* (*Pyrosmia*) *ferruginea* | 34.628817°N | 32.940667°E |
| *Osmia* (*Pyrosmia*) *hellados* | 34.71178°N | 33.004775°E |
| *Osmia* (*Pyrosmia*) *hellados* | 34.755799°N | 33.096194°E |
| *Osmia* (*Pyrosmia*) *submicans* | 34.71178°N | 33.004775°E |
| *Osmia* (*Pyrosmia*) *submicans* | 34.597305°N | 32.984521°E |
| *Osmia* (*Pyrosmia*) *submicans* | 34.755799°N | 33.096194°E |
| *Osmia* (*Pyrosmia*) *submicans* | 34.741952°N | 32.734845°E |
| *Osmia* (*Pyrosmia*) *submicans* | 34.940989°N | 32.872738°E |
| *Osmia* (*Pyrosmia*) *viridana* | 34.71178°N | 33.004775°E |
| *Osmia* (*Pyrosmia*) *viridana* | 34.764269°N | 32.757736°E |
| *Osmia* (*Pyrosmia*) *viridana* | 34.741952°N | 32.734845°E |
| *Osmia* (*Pyrosmia*) *viridana* | 34.745537°N | 32.73385°E |
| *Osmia* (*Pyrosmia*) *viridana* | 34.800043°N | 33.000918°E |
| *Protosmia* (*Protosmia*) *paradoxa* | 34.71178°N | 33.004775°E |
| *Protosmia* (*Protosmia*) *paradoxa* | 34.597305°N | 32.984521°E |
| *Protosmia* (*Protosmia*) *paradoxa* | 34.764269°N | 32.757736°E |
| *Protosmia* (*Protosmia*) *paradoxa* | 34.800043°N | 33.000918°E |
| *Protosmia* (*Protosmia*) *paradoxa* | 34.677579°N | 32.722066°E |
| *Protosmia* (*Protosmia*) *paradoxa* | 34.748126°N | 32.732248°E |
| *Pseudapis* (*Nomiapis*) *diversipes* | 35.05519°N | 33.814011°E |
| *Pseudapis* (*Nomiapis*) *diversipes* | 35.053539°N | 32.351197°E |
| *Sphecodes ephippius* | 34.755799°N | 33.096194°E |
| *Sphecodes monilicornis* | 34.677579°N | 32.722066°E |
| *Sphecodes pseudofasciatus* | 34.755799°N | 33.096194°E |
| *Thyreus histrionicus* | 34.94°N | 32.31°E |
| *Thyreus picaron* | 34.94°N | 32.31°E |
| *Thyreus ramosus* | 34.94°N | 32.31°E |
| *Xylocopa* (*Copoxyla*) *iris* | 34.723986°N | 32.736892°E |
| *Xylocopa* (*Copoxyla*) *iris* | 34.745537°N | 32.73385°E |
| *Xylocopa* (*Copoxyla*) *iris* | 34.67°N | 32.85°E |
| *Xylocopa* (*Copoxyla*) *iris* | 34.72°N | 32.55°E |
| *Xylocopa* (*Copoxyla*) *iris* | 34.9°N | 32.42°E |
| *Xylocopa* (*Copoxyla*) *iris* | 35.06°N | 32.46°E |
| *Xylocopa* (*Copoxyla*) *iris* | 34.900977°N | 32.776759°E |
| *Xylocopa* (*Copoxyla*) *iris* | 34.94°N | 32.31°E |
| *Xylocopa* (*Koptortosoma*) *pubescens* | 34.71178°N | 33.004775°E |
| *Xylocopa* (*Koptortosoma*) *pubescens* | 35.1688°N | 33.367°E |
| *Xylocopa* (*Koptortosoma*) *pubescens* | 34.72°N | 32.55°E |
| *Xylocopa* (*Koptortosoma*) *pubescens* | 34.78°N | 32.4°E |
| *Xylocopa* (*Proxylocopa*) *olivieri* | 34.992°N | 32.9082°E |
| *Xylocopa* (*Xylocopa*) *violacea* | 34.918°N | 32.9472°E |
| *Xylocopa* (*Xylocopa*) *violacea* | 34.992°N | 32.9082°E |
| *Xylocopa* (*Xylocopa*) *violacea* | 34.94°N | 32.31°E |
